# The AMPK kinase Sak1 activates separase and promotes its nuclear localization during mitosis

**DOI:** 10.1083/jcb.202505056

**Published:** 2026-10-20

**Authors:** David Vaquero-Pelaz, José-Antonio Rodriguez-Rodriguez, Malina Carmina Ilinca, Esther Casas-Fernández, Amparo Picard, Francisco Del Caño-Ochoa, Isabel Vernia, Toni Gabaldón, Maria Angeles Juanes, Ethel Queralt

**Affiliations:** 1 Instituto de Biomedicina de Valencia (IBV-CSIC), Valencia, Spain; 2 Molecular Biology Program, Sloan Kettering Institute, Memorial Sloan Kettering Cancer Center, New York, NY, USA; 3 Structure of Macromolecular Targets Unit, Instituto de Biomedicina de Valencia (IBV), CSIC, Valencia, Spain; 4 Valencia Biomedical Research Foundation, Centro de Investigación Príncipe Felipe (CIPF), Associated Unit to the Instituto de Biomedicina de Valencia (IBV), Valencia, Spain; 5 Life Sciences Department, Barcelona Supercomputing Center (BSC), Barcelona, Spain; 6 Mechanisms of Disease Program, Institute for Research in Biomedicine (IRB), The Barcelona Institute of Science and Technology, Barcelona, Spain; 7 ICREA, Pg. Lluis Companys 23, Barcelona, Spain; 8 Centro de Investigación Biomédica En Red de Enfermedades Infecciosas, Barcelona, Spain; 9 National Horizons Centre, Teesside University, Darlington, UK; 10 Centro de Investigacion Principe Felipe, Valencia, Spain

## Abstract

Accurate chromosome segregation during mitosis requires the precise regulation of separase, a protease that cleaves cohesin to trigger the separation of sister chromatids. Here, we identify the AMPK-activating kinase Sak1 as a novel regulator of separase in *Saccharomyces cerevisiae*. Using a suppressor screen, we discovered a hypermorphic *SAK1-E50K* allele that restored separase activity and nuclear localization in *esp1-2* and *pds1Δ* mutants. We demonstrated that Sak1 phosphorylates separase independently of Snf1 activation, enhancing its enzymatic function and promoting nuclear accumulation. Loss of Sak1 delays Cdc14 activation and phenocopies FEAR pathway defects, establishing Sak1 as a mitotic regulator. Extending these findings to humans, we demonstrated that human separase and the tumor suppressor kinase LKB1, which is homologous to Sak1, physically associate. This finding reveals an evolutionarily conserved regulatory link between LKB1 and separase, highlighting the kinase-protease interplay as a critical mechanism for ensuring faithful mitotic progression.

## Teaser

Vaquero-Pelaz D et al. show that the kinase Sak1 activates separase, promoting its nuclear accumulation and mitotic function. A hypermorphic *SAK1-E50K* allele boosts separase activity, while human LKB1 associates with separase, revealing a conserved kinase-protease regulatory axis.

## Graphical Abstract

**Figure:**
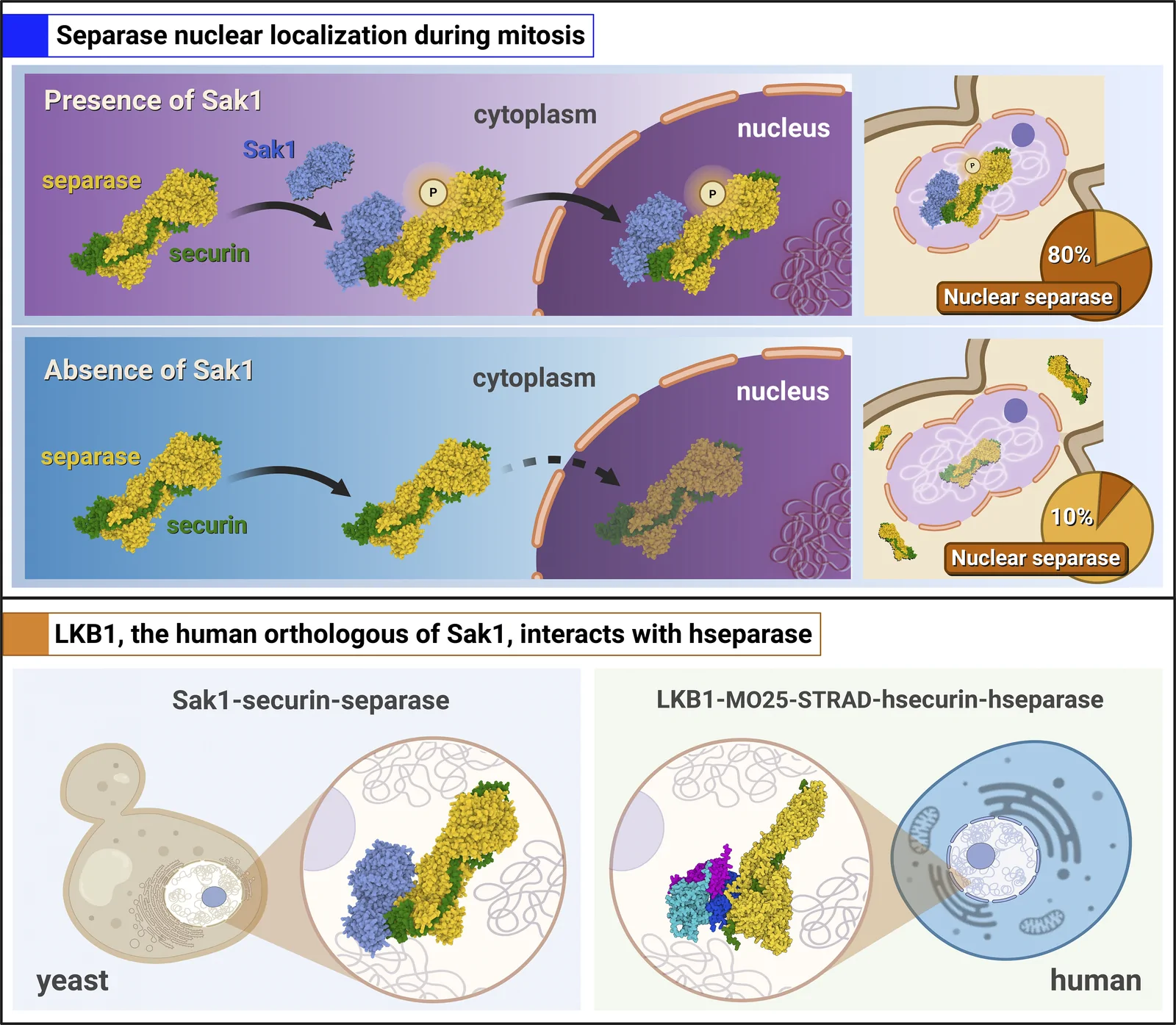

## Introduction

Sister chromatids are held together before mitosis by two key mechanisms: DNA catenation, which involves the intertwining of their DNA strands, and cohesin complexes, specialized protein structures that physically link the chromatids. At anaphase onset, these cohesin links are abruptly dissolved by separase (Esp1 in budding yeast), which cleaves the kleisin subunit Scc1/RAD21 (Uhlmann et al., 2000), allowing chromatid separation. Recent structural work has detailed how human separase recognizes its substrates through multiple docking interfaces and phosphorylation-dependent interactions (Yu et al., 2025). The precise timing and regulation of this cleavage are crucial, as errors can lead to improper chromosome segregation, resulting in genomic instability—a hallmark of many diseases, including cancer. Thus, separase plays a central role in ensuring the accurate and timely segregation of genetic material during mitosis (reviewed by Kamenz and Hauf [2017]; Kumar [2016]).

Beyond cohesin cleavage, separase contributes to diverse cellular processes across species. In budding yeast, these include Cdc14 activation (Stegmeier et al., 2002; Sullivan and Uhlmann, 2003; Queralt et al., 2006; Queralt and Uhlmann, 2008; Calabria et al., 2012; Játiva et al., 2019), spindle elongation and stabilization (Nagao and Yanagida, 2006; Higuchi and Uhlmann, 2005), spindle pole body duplication (Baum et al., 1988), DNA repair (Nagao et al., 2004; McAleenan et al., 2013; Sun et al., 2009), rDNA condensation and resolution (Sullivan et al., 2004; D’Amours et al., 2004), Ty1 retrotransposition (Ho et al., 2015), and apoptosis (Yang et al., 2008). In fission yeast, separase also plays a role in spindle pole body morphology and positioning (Uzawa et al., 1990; Funabiki et al., 1996a; Kumada et al., 1998; Nakamura et al., 2002). In mice, separase is essential for embryonic viability and meiotic chromosome segregation (Wirth et al., 2006; Kudo et al., 2006), and in human cells, it ensures proper mitotic entry and spindle assembly (Sak et al., 2008).

Separase has also emerged as a potential oncoprotein: its overexpression induces aneuploidy and tumorigenesis (Pati, 2008; Zhang et al., 2008), whereas its depletion causes genomic instability (Waizenegger et al., 2002). Altered separase activity or expression is linked to numerous cancers (McAleenan et al., 2013; Pati et al., 2004; Zhang et al., 2008; Meyer et al., 2009; Xu et al., 2011; Waizenegger et al., 2002; Finetti et al., 2014; Mukherjee et al., 2014; Haaβ et al., 2012; Prinzhorn et al., 2016).

To safeguard chromosome segregation, separase is tightly controlled by multiple mechanisms: (1) inhibition by securin (Ciosk et al., 1998; Zou et al., 1999; Jallepalli and Lengauer, 2001; Hornig et al., 2002; Cohen-Fix et al., 1996; Funabiki et al., 1996b; Hatano et al., 2016), (2) inhibitory phosphorylation (Yamamoto et al., 1996; Leismann et al., 2000; Stemmann et al., 2001; Boos et al., 2008), (3) autocleavage in humans and flies (Herzig et al., 2002; Waizenegger et al., 2002; Zou et al., 2002; Chestukhin et al., 2003), and (4) inhibitory binding of Cdk1-cyclin B1 in vertebrate cells (Gorr et al., 2005, 2006; Boos et al., 2008).

Securin binds both the conserved C terminus and the divergent N terminus of separase (Lin et al., 2016; Luo and Tong, 2017; Hornig et al., 2002), acting as a pseudo-substrate that blocks access to the active site (Lin et al., 2016; Luo and Tong, 2017; Nagao and Yanagida, 2006). At anaphase onset, securin is degraded via ubiquitin-dependent proteolysis (Cohen-Fix et al., 1996; Funabiki et al., 1996b; Zachariae and Nasmyth, 1999; Hatano et al., 2016), releasing separase to cleave cohesin. Beyond inhibition, securin is essential for the nuclear localization of separase during mitosis and enhances its proteolytic activity (Jensen et al., 2001; Hornig et al., 2002). In addition, securin is required to fold and stabilize separase properly (Funabiki et al., 1996a; Jallepalli et al., 2001), thus serving as both a negative and positive regulator. Nonetheless, separase still localizes to the nucleus in *pds1Δ* cells, albeit inefficiently (Cohen-Fix et al., 1996; Agarwal and Cohen-Fix, 2002; Hornig et al., 2002), suggesting additional regulatory pathways.

Despite these regulatory mechanisms, a major gap remains in understanding how separase’s diverse functions are coordinated, particularly its nuclear localization independent of securin. Here, we identify the kinase Sak1 as a previously unrecognized regulator of separase, essential for its global control and independent of securin. Through a suppressor screen of the temperature-sensitive separase allele *esp1-2,* we uncover a functional link between separase and the Snf1-activating kinase Sak1. Sak1 physically and genetically interacts with separase, independently of its role in Snf1 activation, and the *SAK1-E50K* suppressor mutation rescues key mitotic defects of the *esp1-2* mutant. We show that Sak1 phosphorylates separase and modulates its nuclear accumulation during mitosis. Ultimately, this study not only identifies Sak1 as a novel separase regulator but also provides significant insights into the intricate mechanisms governing separase activity and subcellular localization during mitosis. This discovery underscores the importance of redundant regulatory layers in ensuring faithful chromosome segregation, with potential implications for understanding genomic instability in human disease.

## Results

### Genetic interactions between *SAK1* and *ESP1*

**Figure S1.**
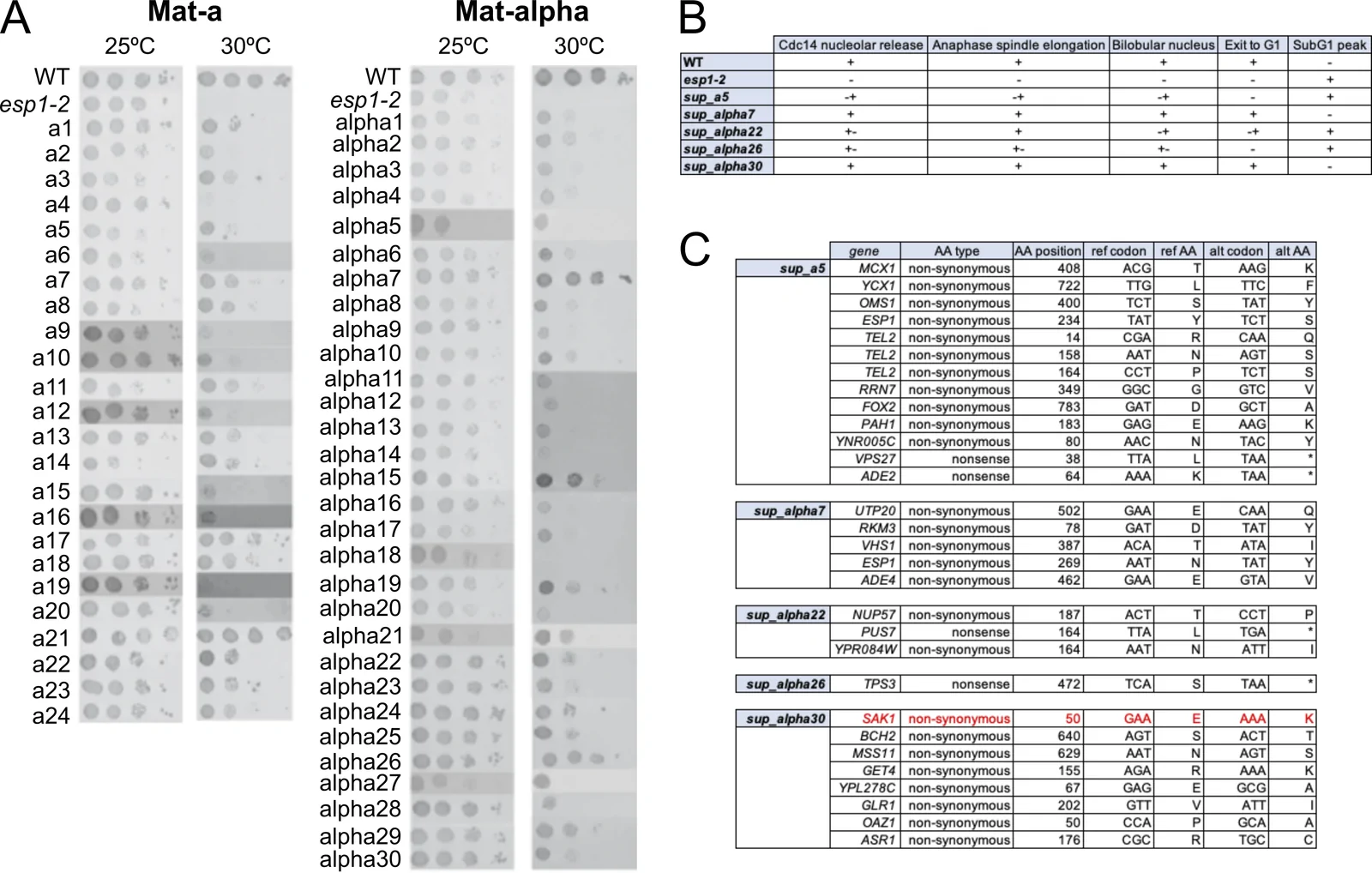
**Suppressor mutants of the *esp1-2* mutant allele. (A)** Growth analysis of *esp1-2* suppressor mutants. Serial dilutions of the indicated cells were spotted onto YPD plates and incubated at 25 and 30°C for 2–3 days. **(B)** Characterization of five selected *esp1-2* suppressor mutants. Five suppressors showing different growth phenotypes were selected for further studies. Cells were synchronized at the metaphase-to-anaphase transition by Cdc20 depletion and re-addition. Cdc14 nucleolar release and anaphase spindle elongation were examined by *in situ* immunofluorescence. Nuclear segregation was assessed by DAPI staining. Mitotic progression and exit into the next G1 phase were determined by FACS analysis of DNA content. **(C)** Summary of the SNP variants (non-synonymous and nonsense) identified by whole-genome sequencing of the five selected suppressor mutants. A complete list is provided in Table S1. Vhs1 associates with Cdc14 and Net1 (Breitkreutz et al., 2010) and is involved in the regulation of the G1/S transition (Muñoz et al., 2003). Nup57 is a nucleoporin that contributes directly to nucleocytoplasmic transport.

To identify new functions and/or regulators of separase, we performed a genetic screen to isolate suppressor mutants of the *esp1-2* mutant allele (Fig. S1 and Table S1). The *esp1-2* mutant was inoculated at 30°C (a semi-restrictive temperature), and the resulting colonies were presumed to contain suppressor mutants that had acquired spontaneous mutations in the *esp1-2* genetic background. One suppressor, designated suppressor-30, was selected based on its ability to rescue multiple separase functions and was subjected to whole-genome sequencing. This analysis identified a mutation in *SAK1-E50K,* which encodes the yeast ortholog of the *S*nf1-*a*ctivating *k*inase, the yeast ortholog of the tumor suppressor LKB1. The best-characterized function of Sak1 is the activation of the Snf1 kinase (the AMP-dependent kinase in *Saccharomyces cerevisiae*, the ortholog of AMPK) under low glucose and stress conditions (reviewed by Hedbacker and Carlson [2008]). Additionally, Sak1 has been implicated in DNA replication and repair, as its overexpression suppresses mutations in DNA polymerase (Hovland et al., 1997) and interacts with Pso2 nuclease in response to DNA damage (Munari et al., 2014).

**Figure 1.**
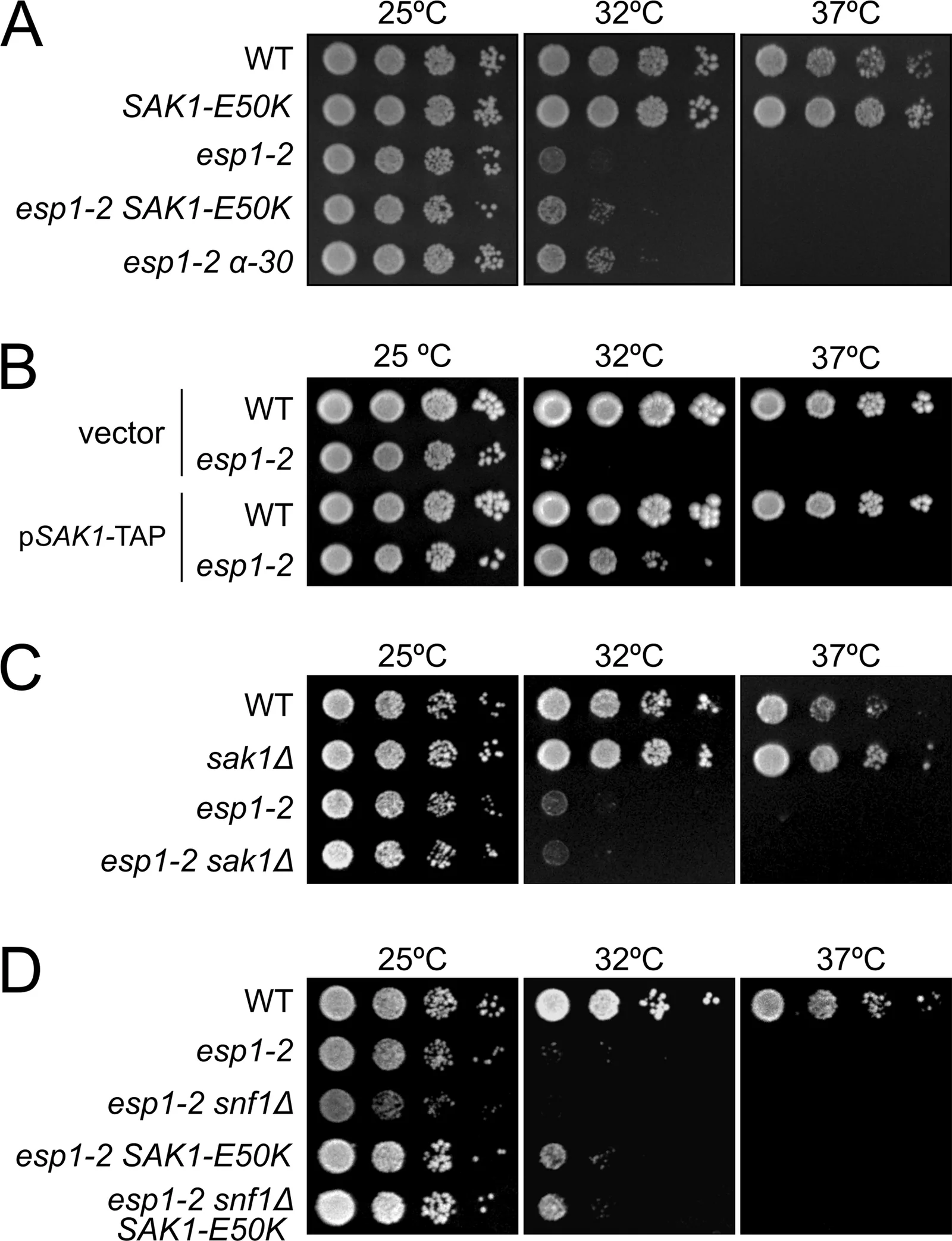
***SAK1-E50K* is a suppressor mutation of the thermosensitive separase allele *esp1-2*.** Serial dilutions of the indicated cells were spotted on YPD plates and grown at 25, 32, and 37°C for 2–3 days. **(A)***SAK1-E50K* mutation suppresses the lethality of the *esp1-2* mutant allele at semi-restrictive temperature. Strains WT (W303), *SAK1-E50K* (Y1698), *esp1-2* (Y500), *esp1-2 SAK1-E50K* (Y1692), and *esp1-2* α30. **(B)***SAK1* overexpression rescues the growth of *esp1-2* cells at the semi-restrictive temperature. Strains WT (W303) and *esp1-2* (Y500) were transformed with an empty vector or the pSAK1-TAP. **(C)** Deletion of *SAK1* does not exacerbate the conditional lethal phenotype of the *esp1-2* mutant. Strains WT (W303), *sak1Δ* (Y1052), *esp1-2* (Y500), and *esp1-2 sak1Δ* (Y1393). **(D)** Suppression of the *esp1-2* allele by *SAK1-E50K* is independent of Snf1. Strains WT (W303), *esp1-2* (Y500), *esp1-2 snf1Δ* (Y2132), *esp1-2 SAK1-E50K* (Y1692), and *esp1-2 SAK1-E50K snf1Δ* (Y2134). All images are representative of at least three independent biological experiments. Source data are available for this figure: SourceData F1.

To validate *SAK1-E50K* as the causative suppressor mutation, the suppressor-30 was backcrossed three times with the WT strain W303. Segregants exhibiting the suppressor phenotype (growth at 32°C but lethality at 37°C) were subjected to DNA extraction and Sanger sequencing, confirming the presence of *esp1-2* (Y234S) and *SAK1-E50K* mutations. Tetrad analysis demonstrated that the suppressor phenotype co-segregated with the *SAK1-E50K* mutation (Fig. 1 A). To further confirm the genetic interaction, the *esp1-2* mutant strain was transformed with a 2-µm multicopy plasmid carrying *SAK1* (p*SAK1*-TAP). As shown in Fig. 1 B, the plasmid rescued the conditional lethality of the *esp1-2* mutant at 32°C, providing additional evidence that suppressor-30 is allelic to *SAK1*. These data also suggest that *SAK1-E50K* is a hypermorphic allele of Sak1.

To further investigate this interaction, we deleted *SAK1* in the *esp1-2* mutant strain. The resulting *esp1-2 sak1Δ* double mutant displayed a growth phenotype similar to the single *esp1-2* mutant at 32°C (Fig. 1 C). Thus, deletion of *SAK1* does not exacerbate the conditional lethality of *esp1-2*, suggesting that Sak1 is not essential for the viability of the *esp1-2* mutant. These results indicate that Sak1 is not required for separase function; however, increased Sak1 activity may enhance separase efficiency.

### The functional link between separase and Sak1 is independent of Snf1

Sak1 is one of the three kinases responsible for phosphorylating and activating Snf1 under nutrient-deprivation conditions, particularly during glucose deprivation (Nath et al., 2003; McCartney and Schmidt, 2001; Elbing et al., 2006; Hong and Carlson, 2007). The Snf1/AMPK pathway regulates cellular adaptation to alternative carbon sources and serves as a key sensor of cellular stress (Nath et al., 2003; Hong and Carlson, 2007; McCartney et al., 2005; Elbing et al., 2006). To determine whether the genetic interaction between *ESP1* and *SAK1* depends on Sak1’s ability to activate Snf1, we studied the *esp1-2 SAK1-E50K snf1Δ* triple mutant. This triple mutant retained the ability to suppress the *esp1-2* conditional lethal phenotype, similar to the *esp1-2 SAK1-E50K* double mutant (Fig. 1 D). These results indicate that the *ESP1*-*SAK1* genetic interaction is not mediated by increased Snf1 activity due to *SAK1-E50K* but is instead related to an alternative function of Sak1.

**Figure S2.**
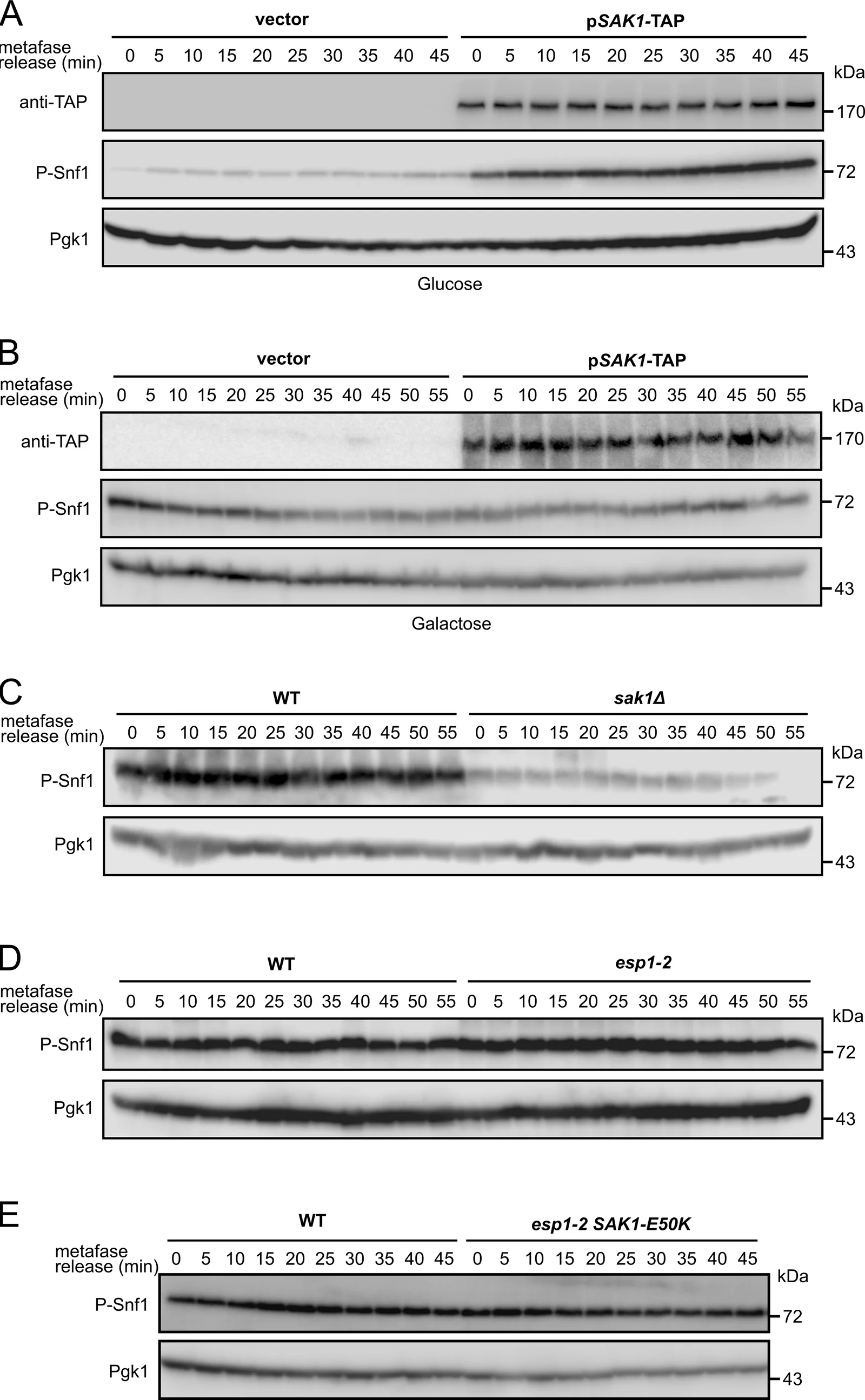
**The *SAK1-E50K* mutation does not promote Snf1 phosphorylation. (A)** Ectopic expression of Sak1 (p*SAK1*-TAP) induces Snf1 phosphorylation in glucose media. Strain *MET3-CDC20* (Y1056) transformed with an empty vector or p*SAK1*-TAP was grown in glucose as a carbon source, arrested in metaphase, and released into anaphase by Cdc20 depletion and re-addition. **(B)** Snf1 is phosphorylated in the presence of galactose as an alternative carbon source in control cells. Strain *GAL1*-*CDC20* (Y1656) containing an empty vector or p*SAK1*-TAP was grown in galactose as a carbon source, arrested in metaphase, and released into anaphase by Cdc20 depletion and re-addition. **(C)** Snf1 phosphorylation is reduced in the absence of Sak1. Strains *GAL1*-*CDC20* (Y1656) and *sak1Δ GAL1*-*CDC20* (Y1655) were grown in galactose and synchronized in the metaphase-to-anaphase transition by Cdc20 depletion. **(D)** Separase activity does not regulate Snf1 phosphorylation. Strains *GAL1*-*CDC20* (Y1656) and *esp1-2 GAL1*-*CDC20* (Y1686) were grown in galactose as a carbon source, arrested in metaphase by Cdc20 depletion, and shifted to 32°C to inactivate *esp1-2* for 3 h before release into anaphase. **(E)** Snf1 phosphorylation is unaffected in *esp1-2 SAK1-E50K* mutant cells. Strains *GAL1*-*CDC20* (Y1656) and *esp1-2 SAK1-E50K GAL1*-*CDC20* (Y1694) were grown in galactose, arrested in metaphase by Cdc20 depletion, shifted to 32°C for 3-h intervals, and released into anaphase. P-Snf1 levels were detected by western blotting, with Pgk1 used as a loading control. Blots are representative of at least three independent biological experiments. Source data are available for this figure: SourceData FS2.

Sak1 primarily phosphorylates Snf1 at T210, a modification required for Snf1 activation when cells are grown on alternative carbon sources, such as galactose (Nath et al., 2003; Amodeo et al., 2010). As previously reported (García-Salcedo et al., 2014), overexpression of Sak1 (p*SAK1*-TAP) induced Snf1 phosphorylation even under glucose-rich conditions, whereas Snf1 phosphorylation was largely undetectable in mitotically synchronized control cells (Fig. S2 A). Therefore, we analyzed Snf1 phosphorylation under galactose conditions to enhance its activation. As expected, Snf1 phosphorylation increased in the WT strain (Fig. S2 B) but was significantly reduced in the absence of Sak1 (Fig. S2 C). However, no differences in Snf1 phosphorylation were observed in *esp1-2* (Fig. S2 D) or *esp1-2 SAK1-E50K* (Fig. S2 E) mutants.

These findings reinforce the conclusion that the genetic interaction between *esp1-2* and *SAK1-E50K* is independent of Snf1 and confirm that Snf1 and Sak1 remain functional in the *esp1-*2 mutant. Moreover, they demonstrate that the suppression phenotype observed in *esp1-2 SAK1-E50K* is not due to Sak1-mediated regulation of Snf1 activity.

### *SAK1-50K* rescues mitotic progression defects of the *esp1-2* mutant allele

The conditional separase mutant *esp1-2* exhibits impaired cohesin cleavage, leading to defects in chromosome segregation (Buonomo et al., 2000; Uhlmann et al., 2000; Queralt et al., 2006), defective mitotic spindle elongation (Baskerville et al., 2008; Jensen et al., 2001), and delayed Cdc14 nucleolar release due to compromised phosphorylation of Net1 and Cdc55 (Stegmeier et al., 2002; Azzam et al., 2004; Queralt et al., 2006). To determine the mechanism by which *SAK1-E50K* rescues the conditional lethality of *esp1-2,* we examined key mitotic events in WT, *esp1-2,* and *esp1-2 SAK1-E50K* cells.

**Figure 2.**
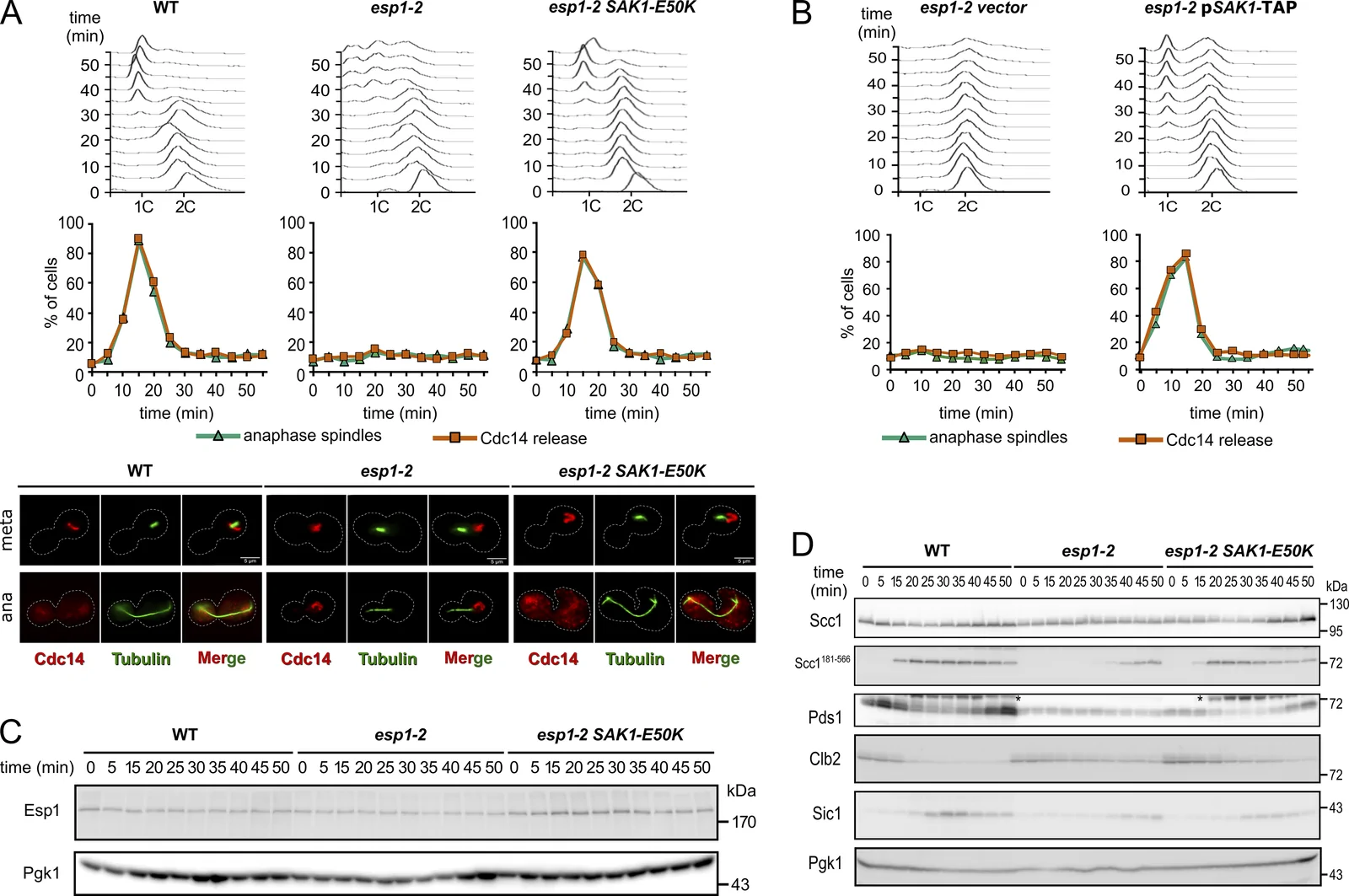
**The suppressor mutation *SAK1-E50K* recovers the mitotic defects of the *esp1-2* allele. (A)** The *esp1-2 SAK1-E50K* double mutant shows chromosome segregation, spindle elongation, and Cdc14 release from the nucleolus. Strains WT (*GAL1*-*CDC20,* Y1656), *esp1-2 GAL1*-*CDC20* (Y1686), and *esp1-2 SAK1-E50K GAL1*-*CDC20* (Y1694) were arrested in metaphase by Cdc20 depletion and shifted to 32°C to inactivate *esp1-2* 3 h before release into anaphase. Mitotic progression was followed by FACS analysis of the DNA content. Cdc14 nucleolar release and anaphase spindle elongation were visualized using *in situ* immunofluorescence. At least 100 cells were scored at each time point. Representative images show cells in metaphase arrest (meta) and 20 min after release (ana). Scale bar, 5 μm. **(B)** Ectopic expression of *SAK1* restores chromosome segregation, spindle elongation, and Cdc14 release in the *esp1-2* mutant allele. Strain *esp1-2 GAL1*-*CDC20* (Y1686) transformed with an empty vector or the pSAK1-TAP was arrested in metaphase and released into anaphase by Cdc20 depletion and re-addition. **(C)** Separase protein is not degraded at the semi-restrictive temperature in *esp1-2* mutants. Strains WT (*ESP1-6HA MET3-CDC20*, Y1955), *esp1-2-6HA MET3-CDC20* (Y1944), and *esp1-2-6HA SAK1-E50K MET3-CDC20* (Y1947) were arrested in metaphase and released into anaphase by Cdc20 depletion and re-addition. Separase protein levels were analyzed by western blotting using Pgk1 as a loading control. **(D)** The *SAK1-E50K* mutation restores Scc1 cleavage, Pds1 and Clb2 degradation, and Sic1 accumulation during anaphase in the *esp1-2* mutant. Strains *SCC1-6HA MET3-*CDC20 (Y2086), Y1927 (as Y2086, but *esp1-2*), Y1930 (as Y1927, but *SAK1-E50K*), *PDS1-6HA MET3-CDC20* (Y1934), Y2123 (as Y1934, but *esp1-2*), Y2121 (as Y2123, but *SAK1-E50K*), *SIC1-6HA MET3-CDC20* (Y2049), Y2113 (as Y2049, but *esp1-2*), and Y2116 (as Y2113, but *SAK1-E50K*) were arrested in metaphase and released into anaphase by Cdc20 depletion and re-addition. Scc1, Pds1, Clb2, and Sic1 proteins were analyzed using Western blotting. The images for full-length Scc1 and cleaved Scc1 represent two different exposures of the same membrane. (*) Marks the Cdc20-HA band detected by the anti-HA antibody. Results are representative of at least three independent biological experiments. Source data are available for this figure: SourceData F2.

Cells were synchronized in metaphase by depleting the APC activator Cdc20, expressed under the methionine-repressible *MET3* promoter, and subsequently shifted to a semi-restrictive temperature (32°C) to inactivate the *esp1-*2 allele 3 h before release into anaphase. Upon Cdc20 re-induction at 32°C, *esp1-*2 cells exhibited the expected mitotic defects, including impaired Cdc14 nucleolar release, defective spindle elongation, and delayed mitotic exit (Fig. 2 A). Furthermore, chromosome missegregation led to the formation of aneuploid daughter cells, as confirmed by cytometry analysis. Strikingly, cohesin cleavage and chromosome segregation were fully rescued in the *esp1-2 SAK1-E50K* double mutant, and Cdc14 release from the nucleolus and spindle elongation were also restored to a greater extent, suggesting that *SAK1-E50K* restores key separase-regulated processes.

**Figure S3.**
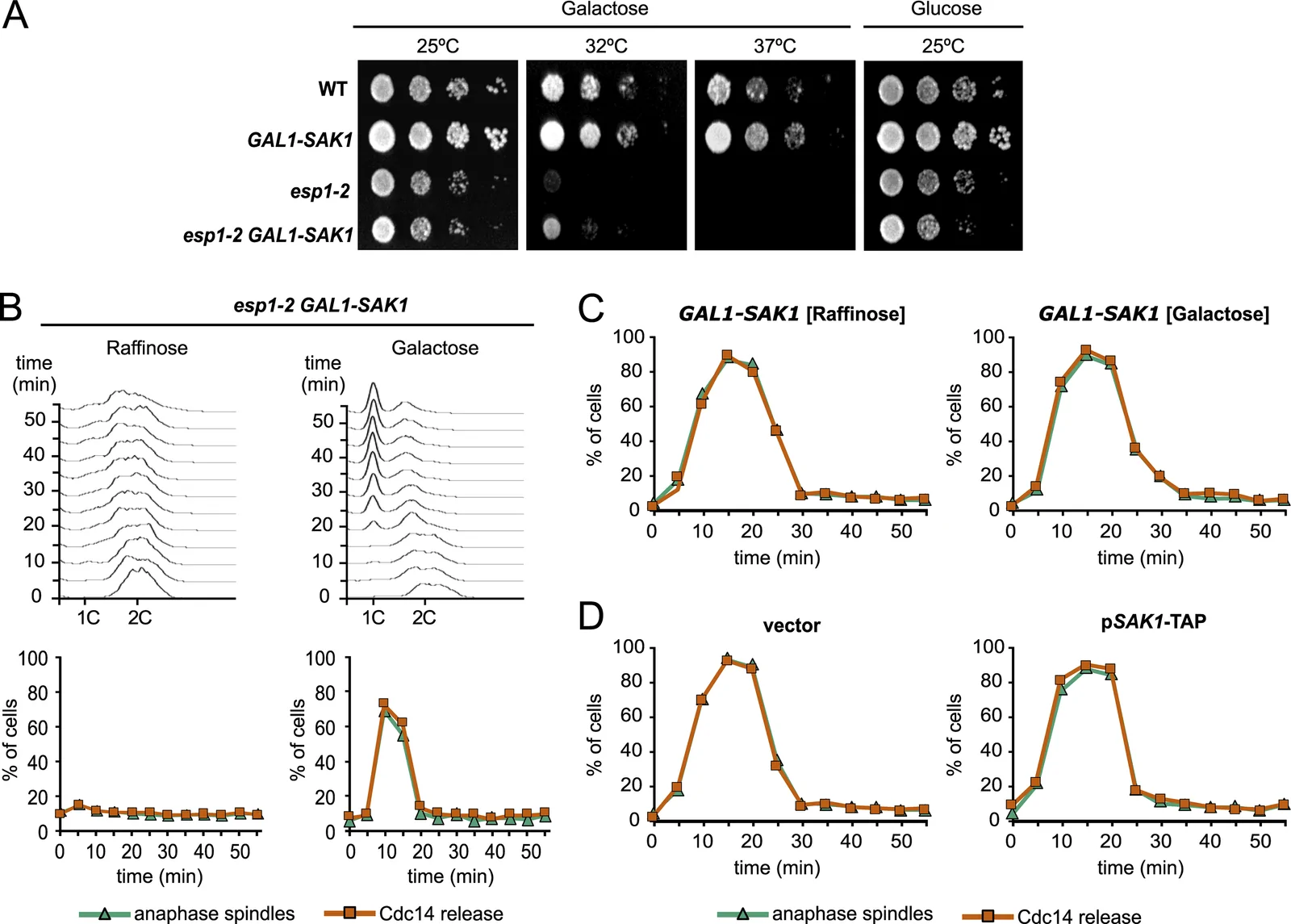
**Ectopic expression of Sak1 rescues the unviability of the thermosensitive separase allele *esp1-2*. (A)** *O*verexpression of *SAK1* rescues the growth of the *esp1-2* mutant allele at semi-restrictive temperature. Serial dilutions of strains WT (W303), *esp1-2* (Y500), *GAL1-SAK1-TAP* (Y1779), and *esp1-2 GAL1-SAK1-TAP* (Y1790) were spotted on YPD or YPGal plates and grown at 25, 32, and 37°C for 2–3 days. **(B)** Ectopic expression of *SAK1* under the galactose-inducible promoter *GAL1* restores chromosome segregation, spindle elongation, and Cdc14 release defects in the *esp1-2* mutant allele. Strain *esp1-2 GAL1-SAK1-TAP MET3-CDC20* (Y1793) was arrested in metaphase in raffinose medium by Cdc20 depletion, shifted to 32°C for 3-h intervals to inactivate *esp1-2*, and released into anaphase in the presence of raffinose (negative control) or galactose to induce Sak1 expression. Mitotic progression was followed by FACS analysis of the DNA content. Cdc14 nucleolar release and anaphase spindle elongation were visualized by *in situ* immunofluorescence. At least 100 cells were scored at each time point. **(C)***SAK1* ectopic expression under the galactose-inducible *GAL1* promoter does not accelerate mitotic progression in the WT background. Strain *GAL1-SAK1-TAP ESP1-6HA MET3-CDC20* (Y2038) was arrested in metaphase in raffinose medium by Cdc20 depletion and released into anaphase in the presence of raffinose (negative control) or galactose to induce Sak1 expression. **(D)***SAK1* overexpression does not alter mitotic progression. Strain *MET3-CDC20* (Y1056) transformed with an empty vector or p*SAK1*-TAP was arrested in metaphase and released into anaphase by Cdc20 depletion and re-addition. Mitotic progression was followed by FACS analysis of the DNA content. Cdc14 nucleolar release and anaphase spindle elongation were visualized by *in situ* immunofluorescence. At least 100 cells were scored at each time point. Images are representative of at least three independent biological experiments. Source data are available for this figure: SourceData FS3.

As the *SAK1-E50K* allele and *SAK1* overexpression similarly suppress the *esp1-2* mutant (Fig. 1, A and B), we repeated the experiment with *SAK1* overexpression (p*SAK1*-TAP). Similar to *SAK1-E50K*, *SAK1* overexpression rescued Cdc14 activation, anaphase spindle elongation, and mitotic exit in *esp1-2* cells (Fig. 2 B). Equivalent results were obtained with *SAK1* ectopic expression under the galactose-inducible *GAL1* promoter (Fig. S3, A and B).

These findings confirm that *SAK1-E50K* is a gain-of-function mutant and suggest that Sak1 either reactivates separase enzymatic activity and/or stabilizes separase protein levels in the *esp1-2* mutant. To distinguish between these possibilities, we introduced an HA epitope at the endogenous *ESP1* locus and monitored separase protein levels during the metaphase-to-anaphase transition. Separase levels generally remain constant throughout the cell cycle, with a slight increase during G2/M (Jensen et al., 2001). Consistent with published data, separase levels in WT cells were stable during metaphase and early anaphase, with a modest reduction in late anaphase (Fig. 2 C). Notably, *esp1-2* and *esp1-2 SAK1-E50K* mutants exhibit comparable separase protein levels, indicating that separase levels are not altered in *esp1-2* at semi-restrictive temperature. These results confirm that separase is present under these conditions and could be functionally reactivated by *SAK1-E50K*.

To directly assess separase proteolytic activity, we monitored Scc1 cleavage by western blotting. In WT cells, Scc1 cleavage coincided with Pds1 degradation, followed shortly by Clb2 degradation and Sic1 accumulation (Fig. 2 D). *esp1-2* cells showed reduced Scc1 cleavage, as expected. In contrast, Scc1 cleavage was restored to WT levels in *esp1-2 SAK1-E50K* cells, suggesting that *SAK1-E50K* rescues the proteolytic function of separase. Moreover, the timing of Sic1 accumulation was comparable between WT and *esp1-2 SAK1-E50K* cells, further supporting the conclusion that Cdc14 activation is restored in the *esp1-2 SAK1-E50K* mutant. Our results demonstrate that the *SAK1-E50K* mutation mainly restores the proteolytic function and partially restores the non-proteolytic function of separase, bypassing the mitotic defects associated with the *esp1-2* mutant allele.

### Delayed nucleolar Cdc14 release in the absence of Sak1

**Figure 3.**
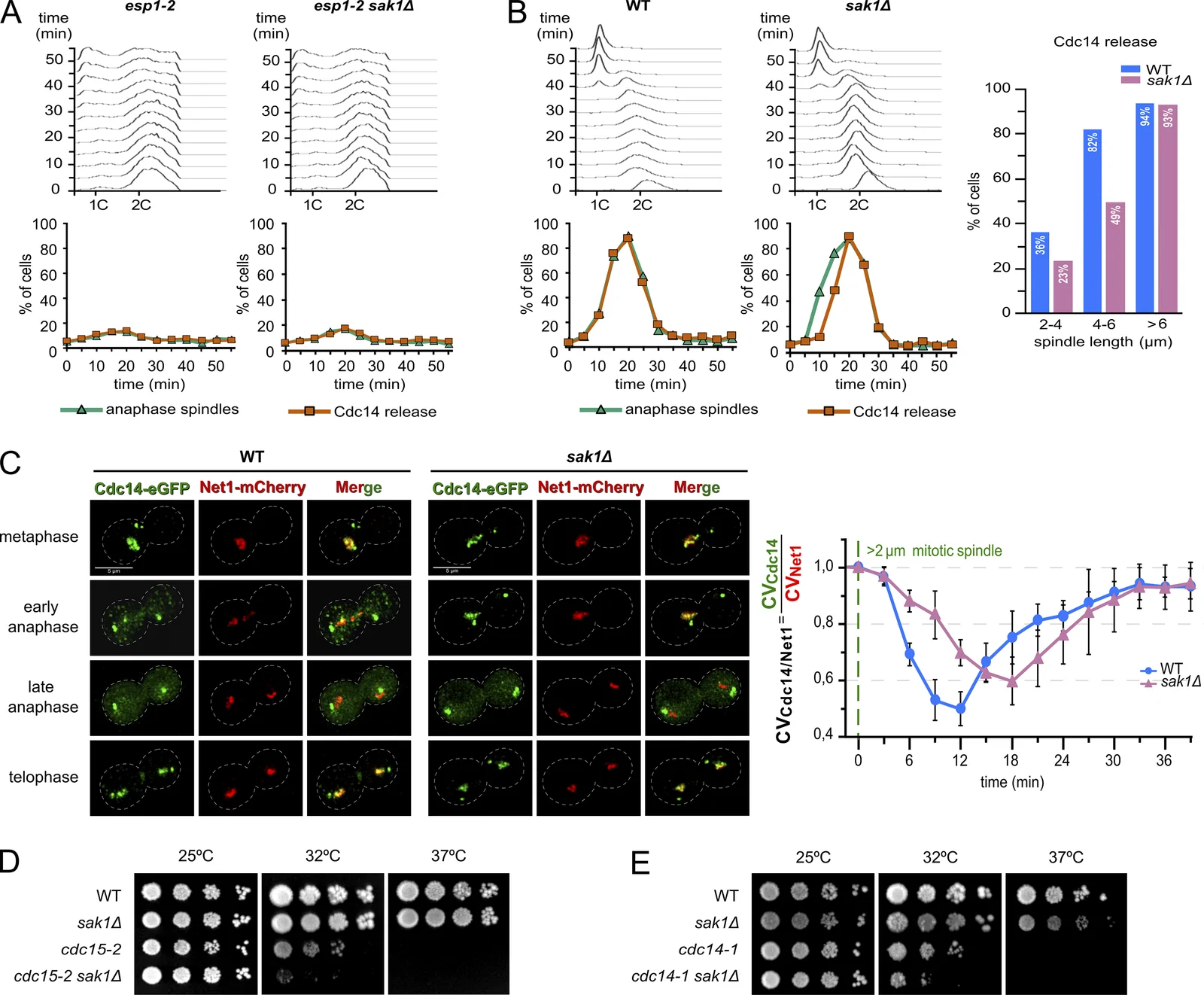
**Sak1 is involved in Cdc14 activation. (A)** Absence of Sak1 does not alter mitotic progression in the *esp1-2* mutant. Cells from strains *esp1-2 MET3*-CDC20 (Y1916) and *esp1-2 SAK1-E50K*, *MET3*-*CDC20* (Y1919) were arrested in metaphase by Cdc20 depletion, shifted to 32°C for 3-h intervals before release into anaphase, and analyzed by FACS and immunofluorescence. At least 100 cells were scored at each time point. Results are representative of at least three independent biological experiments. **(B)** Cdc14 nucleolar release is delayed in early anaphase in the absence of Sak1. Strains WT (*MET3*-*CDC20*, Y1056) and *sak1Δ MET3*-*CDC20* (Y1917) were arrested in metaphase by Cdc20 depletion and released into synchronous anaphase by Cdc20 reintroduction. Quantification of Cdc14 nucleolar release as a function of spindle length is shown (right panel). At least 100 cells were scored for each condition. Data are representative of three independent biological experiments. **(C)** Delayed Cdc14 nucleolar release in Sak1-deficient cells. Strains WT (*NET1-mCherry CDC14-eGFP SPC42-eYFP GAL1-CDC20,* Y1737) and Y1712 (as Y1737, but *sak1Δ*) were arrested in metaphase and released into anaphase by Cdc20 depletion and re-addition, and time-lapse images were captured every 3 min. Representative images show Cdc14-eGFP and Net1-mCherry localization. Cdc14 was quantified at each time point as described in Materials and methods (right panel). At least 30 cells were measured at each time point. The mean and SEM of three independent biological replicates are shown. **(D)***cdc15-2* and *sak1Δ* mutations display a synthetic growth defect. **(E)** Synthetic growth defect observed between *cdc14-1* and *sak1Δ* mutations. Serial dilutions of the WT (W303), *sak1Δ* (Y1052), *cdc15-2* (Y509), *cdc15-2 sak1Δ* (Y1778), *cdc14-1* (Y1262), and *cdc14-1 sak1Δ* (Y1836) cells were spotted on YPD plates and grown at 25, 32, and 37°C for 2–3 days. Images are representative of at least three independent biological experiments. Source data are available for this figure: SourceData F3.

To investigate whether *SAK1* deletion exacerbates the mitotic defects of the *esp1-2* mutant, we arrested *esp1-2* and *esp1-2 sak1Δ* mutant cells in metaphase by depleting Cdc20, followed by a shift to a restrictive temperature (32°C) for 3 h before synchronous release into anaphase. Both mutants exhibited equivalent defects in Cdc14 activation, anaphase spindle elongation, and mitotic exit, indicating that the absence of Sak1 does not further impair mitotic progression in the *esp1-2* background (Fig. 3 A). This observation is consistent with the similar growth defects observed in both strains.

If Sak1 modulates separase activity, then *sak1Δ* cells should exhibit defects in separase-regulated mitotic processes. To test this possibility, we synchronized WT and *sak1Δ* cells at the metaphase-to-anaphase transition. Exit from mitosis and anaphase spindle elongation occur with similar kinetics in WT and *sak1Δ* cells (Fig. 3 B). However, Cdc14 release from the nucleolus was delayed by 5 min relative to the onset of spindle elongation, resembling the delay observed in *spo12Δ* or *zds1Δ* cdcFourteen Early Anaphase Release (FEAR) mutants (Stegmeier et al., 2002; Queralt and Uhlmann, 2008; Calabria et al., 2012). To further analyze this delay, we measured Cdc14 nucleolar release in relation to anaphase spindle length. In *sak1Δ* cells, Cdc14 release was significantly reduced at spindle lengths of 2–6 μm (Fig. 3 C), a pattern reminiscent of FEAR pathway mutants. These findings suggest that Sak1 promotes the timely activation of Cdc14 during early anaphase.

To further characterize the Cdc14 activation delay in the absence of Sak1, we employed quantitative time-lapse microscopy to track the nucleolar localization of Cdc14 (Lu and Cross, 2009; Lu and Cross, 2010; Calabria et al., 2012). We measured the ratio of the coefficients of variation (CVs) of the Cdc14-eGFP and the Net1-mCherry signals—high CV Cdc14:CV Net1 ratios indicate nucleolar sequestration of Cdc14, while a decrease in the ratio marks Cdc14 release. Cells were synchronized at the metaphase-to-anaphase transition by Cdc20 depletion and re-induction, using Spc42-YFP as a control for anaphase progression. In WT cells, Cdc14-eGFP was released from the nucleolus 6 min after anaphase onset and was re-sequestered 15–18 min later (Fig. 3 C). In contrast, *sak1Δ* cells exhibited a delayed release of Cdc14-eGFP (∼12 min after anaphase onset), corroborating the observed Cdc14 nucleolar release delay.

Since FEAR mutants show synthetic lethality with Mitotic Exit Network (MEN) mutants (Azzam et al., 2004; Queralt and Uhlmann, 2008; Stegmeier et al., 2002; Sullivan and Uhlmann, 2003), we investigated whether Sak1 functions in the FEAR pathway by analyzing genetic interactions using a synthetic growth defect assay. The *cdc15-2 sak1Δ* double mutant displayed a synthetic growth defect at 32°C, whereas the *cdc15-2* single mutant remained viable at this temperature (Fig. 3 D). *cdc14* mutants show synthetic lethality with both FEAR and MEN mutants (Jaspersen et al., 1998; Rahal and Amon, 2008; Visintin et al., 1998), as Cdc14 is regulated by both pathways. The *cdc14-1 sak1Δ* double mutant showed a synthetic growth defect at 32°C (Fig. 3 E), consistent with previous observations for FEAR mutants (Visintin et al., 1998; Jaspersen et al., 1998). Our results demonstrate that deletion of Sak1 delays Cdc14 release from the nucleolus and decreases the viability of MEN mutants, strongly supporting a role as a novel component of the FEAR pathway.

### *SAK1-E50K* accelerates separase-induced mitotic exit

**Figure 4.**
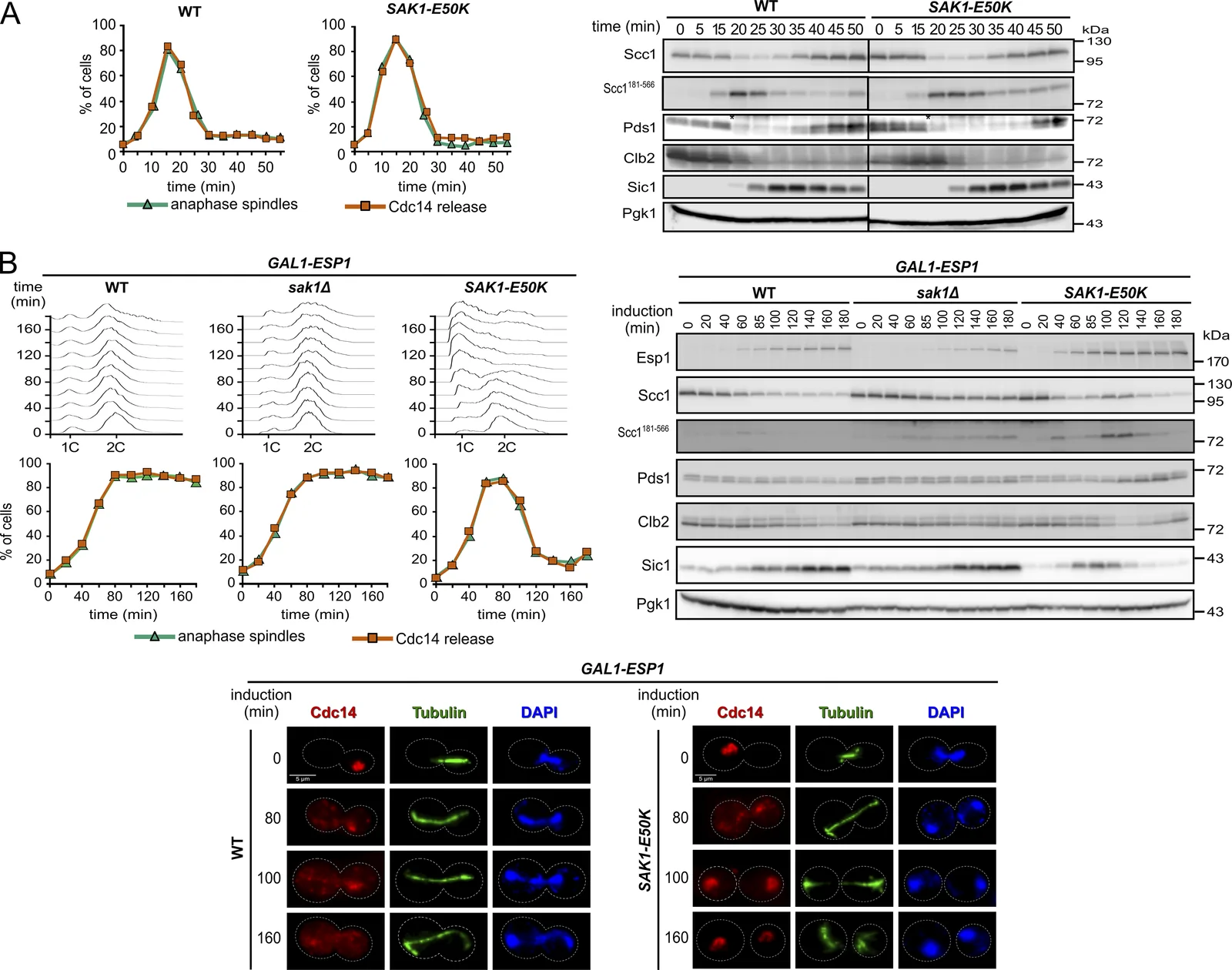
**Ectopic expression of separase in *SAK1-E50K* cells induces exit from mitosis in metaphase-arrested cells. (A)** *SAK1-E50K* mutant does not accelerate mitotic progression in the WT background. Cells from strains *SCC1-6HA MET3-CDC20* (Y1926), Y1929 (as Y1926, but *SAK1-E50K*), *PDS1-6HA MET3-CDC20* (Y1934), Y1937 (as Y1934, but *SAK1-E50K*), *SIC1-6HA MET3-CDC20* (Y2049), and Y2051 (as Y2049, but *SAK1-E50K*) were arrested in metaphase and released into anaphase by Cdc20 depletion and re-addition. Cdc14 nucleolar release and anaphase spindle elongation were visualized by immunofluorescence. At least 100 cells were scored at each time point. Scc1, Pds1, Clb2, and Sic1 proteins were analyzed by western blotting. The images for full-length Scc1 and cleaved Scc1 represent two different exposures of the same membrane. Pgk1 protein levels were used as a loading control. (*) Marks Cdc20 in the western blot. **(B)***SAK1-E50K* promotes exit from mitosis upon separase-induced anaphase. Strains *GAL1-FLAG-ESP1-CBD SCC1-6HA MET3-CDC20* (Y2052), Y2058 (as Y2052, but *sak1Δ*), Y2059 (as Y2052, but *SAK1-E50K*), *GAL1-FLAG-ESP1-CBD PDS1-6HA MET3-CDC20* (Y2037), Y2033 (as Y2037, but *sak1Δ*), Y2032 (as Y2037, but *SAK1-E50K*), *GAL1-FLAG-ESP1-CBD SIC1-6HA MET3-CDC20* (Y2036), Y2034 (as Y2036, but *sak1Δ*), and Y2027 (as Y2036, but *SAK1-E50K*) were arrested in metaphase by Cdc20 depletion, and separase expression was induced by the addition of galactose. Mitotic progression was analyzed by FACS, and Cdc14 release and anaphase spindle elongation were visualized by immunofluorescence. At least 100 cells were scored at each time point. Representative images show cells at metaphase (0 min) and 80, 100, and 160 min after separase induction. Scale bar, 5 μm. Esp1, Scc1, Pds1, Clb2, and Sic1 proteins were analyzed using western blotting. The images for full-length Scc1 and cleaved Scc1 represent two different exposures of the same membrane. Data are representative of at least three independent biological experiments. Source data are available for this figure: SourceData F4.

To determine whether the *SAK1-E50K* mutant exhibits alterations in mitotic progression in an otherwise WT background, we analyzed key mitotic events. As shown in Fig. 4 A, Cdc14 activation, mitotic spindle elongation, and degradation of Clb2 and Pds1 occurred with similar kinetics in WT and *SAK1-E50K* mutant cells. Additionally, neither *SAK1* ectopic expression under the galactose-inducible *GAL1* promoter nor *SAK1* overexpression from a multicopy plasmid affected mitotic progression (Fig. S3, C and D).

Ectopic separase expression during metaphase is sufficient to induce Cdc14 activation, spindle elongation, and chromosome segregation, but only a fraction of cells fully exit mitosis and enter the next cell cycle (Sullivan and Uhlmann, 2003). The ability of separase to release Cdc14 from the nucleolus depends on Slk19 and Spo12, two FEAR pathway components. To determine whether Sak1 is required for separase-induced Cdc14 release, we ectopically expressed separase under the *GAL1* promoter in WT and *sak1Δ* mutant cells, both arrested in metaphase by Cdc20 depletion. Although separase induction was slightly reduced in *sak1Δ* cells, likely due to Sak1’s role in activating Snf1 in galactose medium, resulting in a delay in Scc1 cleavage, Cdc14 release from the nucleolus, and anaphase spindle elongation proceeded with similar kinetics to that observed in control cells (Fig. 4 B).

Strikingly, *the SAK1-E50K* mutation in separase-induced cells accelerated multiple anaphase events, leading to premature mitotic exit, as observed in the FACS profiles (Fig. 4 B). Cdc14 release from the nucleolus, Scc1 cleavage, Clb2 degradation, and Sic1 accumulation occurred significantly earlier in *SAK1-E50K* cells than in WT cells under these conditions. Premature Scc1 cleavage indicates early activation of separase proteolytic activity. As Cdc14 activation in late anaphase promotes APC-Cdh1 activation via Cdh1 dephosphorylation, the premature accumulation of Sic1 and Clb2 degradation suggests that Cdc14 and APC-Cdh1 activation are also accelerated. The absence of Pds1 degradation confirmed that APC-Cdc20 remained inactive, as the cells were maintained in metaphase arrest by Cdc20 depletion. Upon entry into the subsequent S phase, Sic1 degradation was detected, confirming cell cycle progression. Since separase-dependent Cdc14 activation relies on the non-proteolytic function of separase (Sullivan and Uhlmann, 2003), these findings suggest that *SAK1-E50K* enhances both the proteolytic and non-proteolytic activities of separase. This dual activation triggers both early (Scc1 cleavage) and late (APC-Cdh1 activation and mitotic exit) anaphase events, accelerating cell cycle progression.

### Sak1 regulates separase phosphorylation

**Figure 5.**
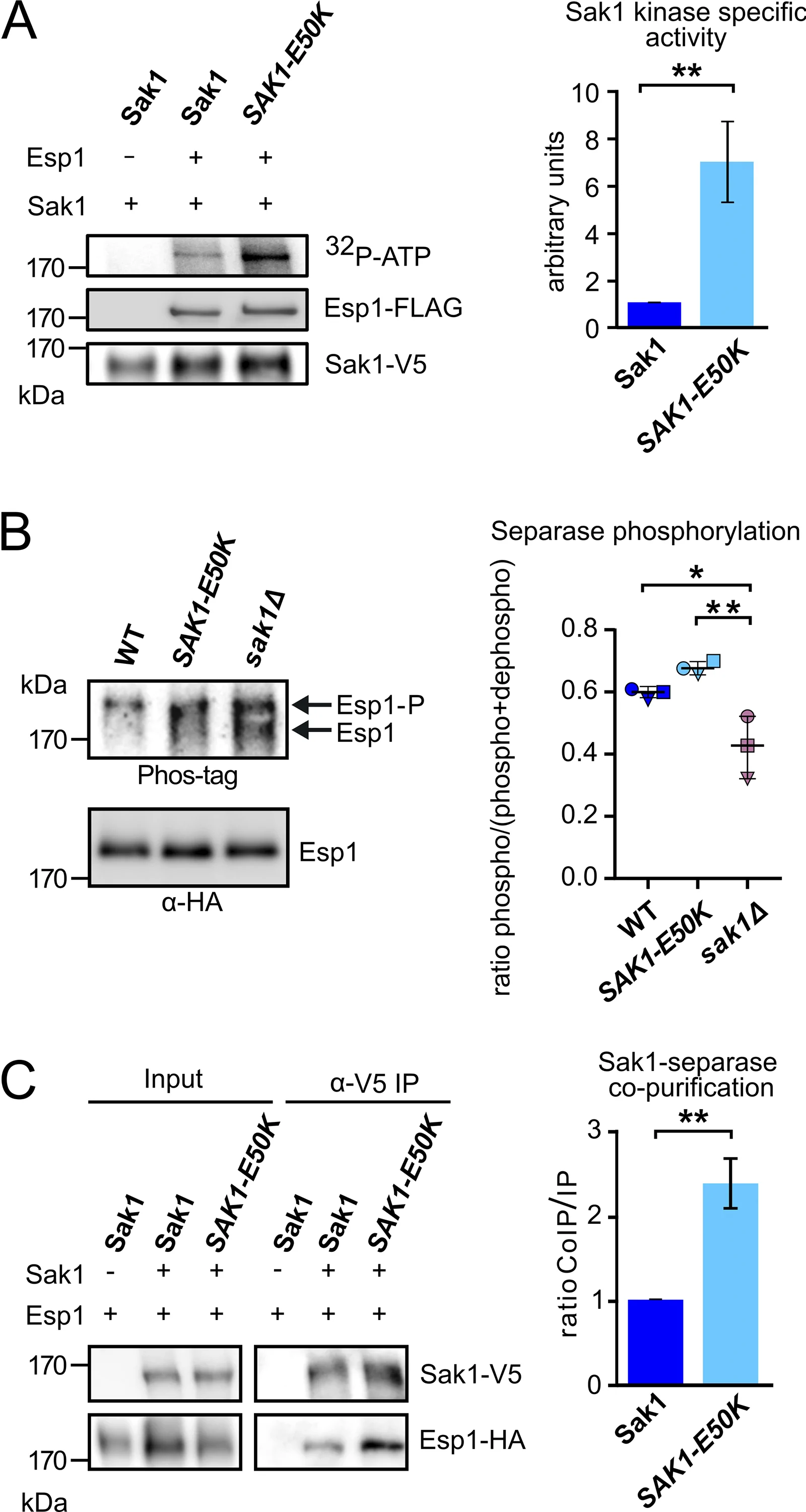
**Sak1 regulates separase phosphorylation. (A)** Sak1 phosphorylates separase *in vitro*. Sak1 and *SAK1-E50K* were immunoprecipitated using a V5 antibody from extracts of the Y1955 (ESP1-6HA SAK1-6V5 MET3-CDC20) and Y1964 (as Y1955, but SAK1-E50K-6V5) strains. Strain Y2086 (*ESP1-6HA MET3-CDC20*) without the V5 epitope was used to prepare Sak1-free purification and used as the negative control. A representative kinase assay is shown. Means and SEMs of four kinase assays are depicted. **(B)** Reduced separase phosphorylation in the absence of Sak1. Separase was immunopurified from protein extracts of strains Y1922 (*ESP1-6HA*), Y1942 (*ESP1-6HA SAK1-E150K*), and Y1941 (*ESP1-6HA sak1Δ*), and separase phosphorylation was analyzed by western blotting using Phos-tag gels. Means and SEMs of the ratio of phosphorylated separase, calculated as the signal of the phosphorylated band (slower-migrating band) divided by the sum of the signals of the phosphorylated and dephosphorylated bands, from three independent biological experiments are shown. Different symbols (circles, triangles and rectangles) represent the three independent biological replicates. **(C)** Sak1 forms a complex with separase. Metaphase-arrested cells of the strains Y1955 (*ESP1-6HA SAK1-6V5 MET3-CDC20*) and Y1964 (as Y1955, but *SAK1-E50K-6V5*) were used to immunopurify Sak1, and the co-immunoprecipitation of separase was analyzed. The strain Y2086 (*ESP1-6HA MET3-CDC20*) without the V5 epitope was used as a negative control. Means and SEMs from three independent biological experiments are represented. Statistical significance: *P < 0.05; ***P < 0.01. Source data are available for this figure: SourceData F5.

Given that Sak1 is a kinase, we hypothesized that Sak1 activates separase via phosphorylation. To test this possibility, we ectopically expressed Esp1-TAP under the galactose-inducible *GAL1* promoter, purified separase, and performed *in vitro* kinase assays. Sak1 and *SAK1-E50K* were immunopurified by pulling down V5-tagged proteins using anti-V5 antibody and Protein A-Dynabeads, and subsequently incubated with TAP-purified separase; phosphorylation levels were assessed by ^32^P incorporation. As shown in Fig. 5 A, ^32^P incorporation was detected when separase was incubated with Sak1, confirming that Sak1 phosphorylates separase *in vitro*. Notably, higher levels of ^32^P incorporation were observed in the *SAK1-E50K* mutant, suggesting that it is a hyperactivating mutation.

**Figure S4.**
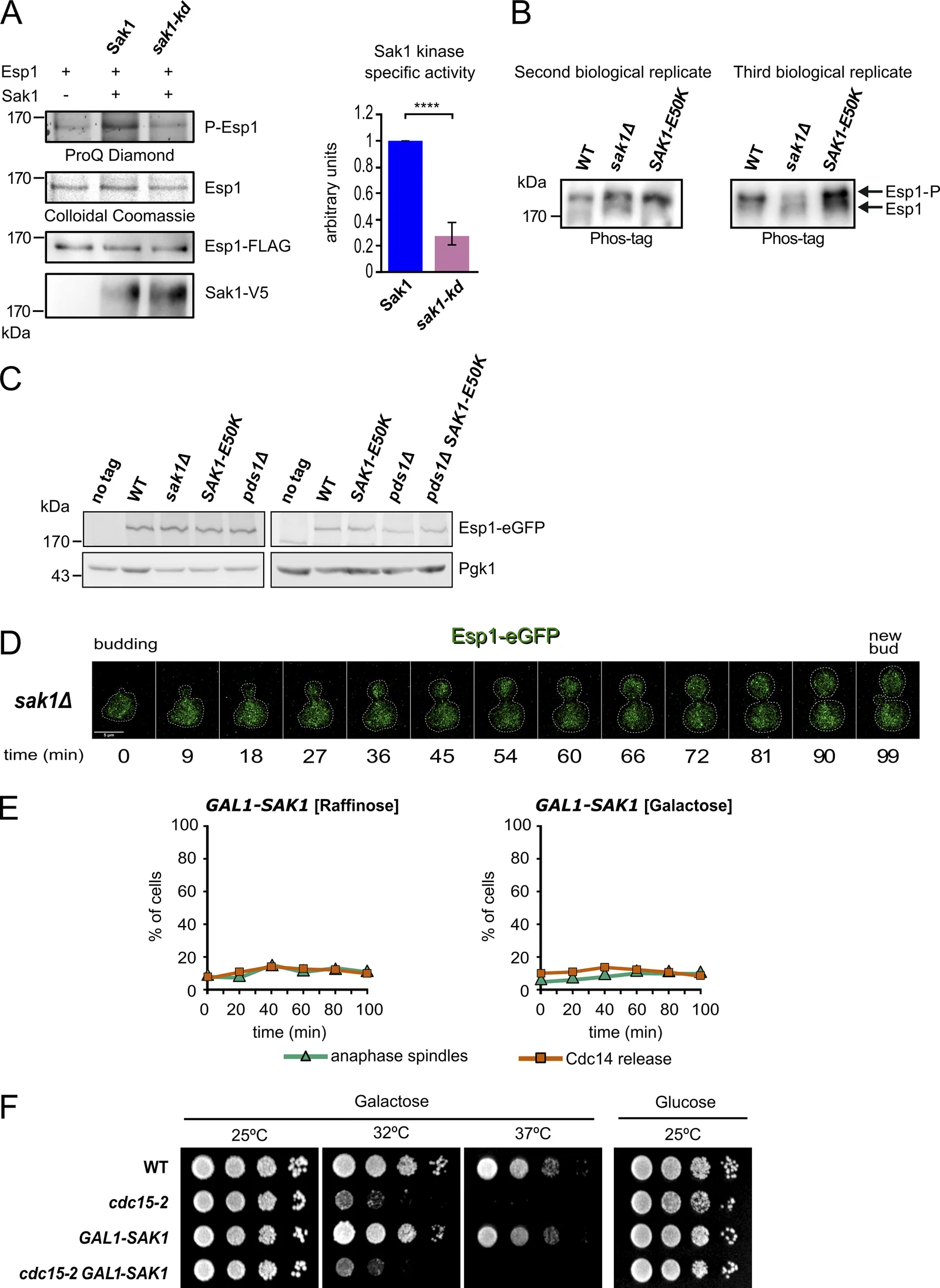
**Sak1 phosphorylates separase and is required for its nuclear accumulation. (A)** Esp1 phosphorylation depends on Sak1 kinase activity. Sak1 and *sak1-D277A* were immunoprecipitated using a V5 antibody from the extracts of strains Y2552 (*sak1Δ SAK1-6V5*) and Y2553 (as Y2552, but *sak1-D277A -6V5*). Strain Y1052 (*sak1Δ*) without the V5 epitope was used to prepare a Sak1-free purification and used as the negative control. A representative kinase assay is shown. Means and SEMs of four kinase assays are depicted. Statistical significance: ****P < 0.0001. **(B)** Biological replicates of separase phosphorylation analyzed by Phos-tag gels. A representative blot is shown in Fig. 5 B, and the two additional replicates are shown here. **(C)** Esp1-eGFP protein levels are not reduced in the absence of securin or Sak1. Protein extracts from strains *HTB2-mCherry ESP1-eGFP MET3-CDC20* (Y2095), *sak1Δ ESP1-eGFP::HIS3 MET3-CDC20* (Y2117), *SAK1-E50K ESP1-eGFP MET3-CDC20* (Y2105), *pds1Δ HTB2-mCherry ESP1-eGFP MET3-CDC20* (Y2162), *HTB2-mCherry ESP1-eGFP* (Y2188), *SAK1-E150K ESP1-eGFP HTB2-mCherry* (Y2161), *pds1Δ HTB2-mCherry ESP1-eGFP* (Y2362), and *pds1Δ SAK1-E50K ESP1-eGFP* (Y2363) were prepared from cycling cells, and Esp1-eGFP visualized by Western blotting. Strains WT (W303) and *MET3-CDC20* (Y1056) were used as a negative control (no tag). Pgk1 was used as the loading control. **(D)** Impaired separase nuclear accumulation in the absence of Sak1. Strain *sak1Δ HTB2-mCherry ESP1-eGFP* (Y2231) was arrested in G1 with α-factor and released into a synchronous cell cycle. Representative images of the Esp1-eGFP signal during one cell cycle are shown. Time zero was defined as the first time point of bud formation. Scale bar, 5 μm. **(E)***SAK1* ectopic expression under the galactose-inducible *GAL1* promoter does not promote Cdc14 release from the nucleolus in metaphase-arrested cells. Strain *MET3-CDC20 GAL1-SAK1-TAP* (Y1785) was arrested in metaphase in raffinose medium by Cdc20 depletion, and *SAK1* expression was induced by galactose addition. Cdc14 nucleolar release and anaphase spindle elongation were visualized using *in situ* immunofluorescence. At least 100 cells were scored at each time point. **(F)***SAK1* overexpression does not rescue the growth of *cdc15-2* cells. Serial dilutions of strains WT (W303), *cdc15-2* (Y509), *GAL1-SAK1-TAP* (Y1792), and *cdc15-2 GAL1-SAK1-TAP* (Y1809) were spotted on YPD or YPGal plates and grown at 25, 32, and 37°C for 2–3 days. Results are representative of at least three independent biological experiments. Source data are available for this figure: SourceData FS4.

To confirm the specificity of Sak1 kinase activity, we repeated the experiment using a Sak1 kinase-dead mutant (*sak1-D277A, sak1-kd*). Esp1 phosphorylation levels, detected by ProQ diamond phosphoprotein staining, were markedly reduced in the *sak1-kd* mutant compared with cells expressing WT Sak1 (Fig. S4 A). These results are consistent with the Sak1-dependent phosphorylation of separase.

To determine whether Sak1 phosphorylates separase *in vivo*, we immunopurified Esp1-HA from cycling cells and analyzed separase isoforms using Phos-tag gels, which distinguish between phosphorylated and dephosphorylated forms of separase. In the absence of Sak1, the ratio of phosphorylated to dephosphorylated separase was reduced compared with WT cells (Fig. 5 B and Fig.S4 B). Conversely, in *SAK1-E50K* cells, this ratio was slightly increased, although the difference did not reach statistical significance, consistent with the elevated Sak1 kinase activity.

Since Sak1 phosphorylates separase, we next investigated whether separase and Sak1 physically interact. To explore this, we immunopurified Sak1-V5 and *SAK1-E50K-V5* from metaphase-arrested cells by Cdc20 depletion and assessed the co-purification of Esp1-HA. As shown in Fig. 5 C, Esp1-HA co-purified with *SAK1-E50K-V5* at higher levels than with WT Sak1, suggesting that the *SAK1-E50K* mutation enhances separase binding affinity.

Collectively, these results demonstrate that Sak1 phosphorylates separase and that *the SAK1-E50K* mutation enhances both separase phosphorylation and its physical interaction with Sak1.

### Sak1 promotes nuclear localization of separase

The localization and activity of separase are tightly regulated by securin to ensure accurate chromosome segregation during mitosis (Agarwal and Cohen-Fix, 2002; Baskerville et al., 2008; Hornig et al., 2002; Jensen et al., 2001). Nuclear localization of separase is greatly reduced, but not abolished, in the absence of securin (Yamamoto et al., 1996; Hornig et al., 2002; Jensen et al., 2001). This suggests an additional mechanism controlling the nuclear localization of separase, and we hypothesized that Sak1-mediated phosphorylation of separase promotes its nuclear localization.

**Figure 6.**
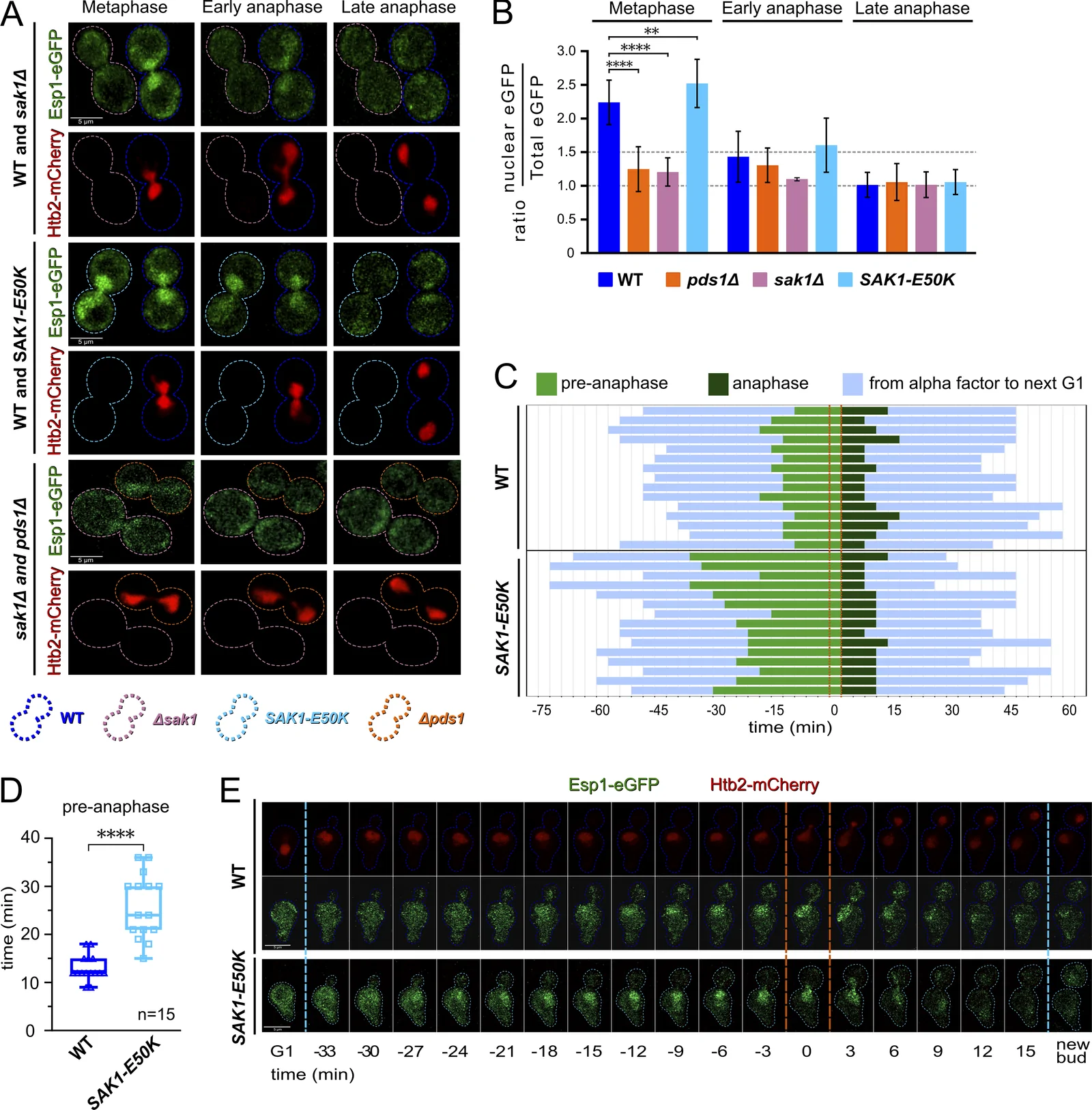
**Sak1 is required for proper nuclear localization of separase. (A)** Impaired nuclear localization of separase in the absence of Sak1. Strains *HTB2-mCherry ESP1-eGFP MET3-CDC20* (Y2095), *SAK1-E50K ESP1-eGFP MET3-CDC20* (Y2105), *sak1Δ ESP1-eGFP MET3-CDC20* (Y2117) and *pds1Δ HTB2-mCherry ESP1-eGFP MET3-CDC20* (Y2162) were arrested in metaphase and released into anaphase by Cdc20 depletion and re-addition, and time-lapse images were captured every 2 min. Representative images of three independent biological experiments are shown. Statistical significance: **P < 0.01, ****P < 0.0001. Scale bar, 5 μm. See also Videos 1, 2, and 3. **(B)** Quantification of the Esp1-eGFP nuclear-to-cytoplasmic ratio from A is depicted. At least 50 cells were scored for each condition. Means and SEMs from three independent biological experiments are shown. **(C–E)** Separase translocates into the nucleus prematurely in *SAK1-E50K* cells. Strains *HTB2-mCherry ESP1-eGFP* (Y2188) and *SAK1-E150K ESP1-eGFP* (Y2161) were arrested in G1 with α-factor and released into cell cycle progression by pheromone removal. Nuclear Esp1-eGFP signal duration for 15 cells during one cell cycle is shown in C. The quantification of cells with nuclear Esp1-eGFP before anaphase (pre-anaphase) is depicted in the box plots (D). Representative images of three independent biological experiments are shown (E). Scale bar, 5 μm. See also Video 4. Statistical significance: ****P < 0.0001.

To test this possibility, we examined Esp1-eGFP localization using time-lapse microscopy during the metaphase-to-anaphase transition by synchronizing cells via Cdc20 depletion and re-induction. In control cells, the Esp1-eGFP signal accumulated in the nucleus during metaphase and early anaphase and diminished during late anaphase (Fig. 6 A; and Videos 1, 2, and 3). Interestingly, in *SAK1-E50K* mutant cells, separase accumulation in the nucleus was increased during metaphase. Conversely, in *sak1*Δ mutant cells, the Esp1-eGFP nuclear signal was reduced compared with control cells, resembling the defect observed in *pds1*Δ mutant cells (Agarwal and Cohen-Fix, 2002; Baskerville et al., 2008; Hornig et al., 2002; Jensen et al., 2001). Quantification of nuclear Esp1-eGFP confirmed that separase nuclear accumulation was similarly impaired in *sak1*Δ and *pds1*Δ mutants (Fig. 6 B and Table S2). Notably, Esp1-eGFP protein levels remained unchanged in the absence of Sak1 or securin (Fig. S4 C), indicating that the reduced nuclear localization was not due to decreased protein levels.

**Video 1.** **Localization of separase in the absence of Sak1.** The *HTB2-mCherry ESP1-eGFP MET3-CDC20* (Y2095) and *sak1Δ ESP1-eGFP MET3-CDC20* (Y2117) strains were arrested in metaphase and released into anaphase by Cdc20 depletion and re-addition. The cells were combined and placed in chambers coated with concanavalin, and time-lapse images were captured using Airyscan2 at 2-min intervals. The videos are presented at a rate of 2 frames/sec. Representative images from three independent biological experiments are shown. Video related to Fig. 6.

**Video 2.** **Premature nuclear localization of separase in *SAK1-E50K* cells.** The strains *HTB2-mCherry ESP1-eGFP MET3-CDC20* (Y2095) and *SAK1-E50K ESP1-eGFP MET3-CDC20* (Y2105) were arrested in metaphase and released into anaphase by Cdc20 depletion and re-addition. The cells were mixed and deposited in concanavalin-coated chambers, and time-lapse images were acquired every 2 min using Airyscan2. The videos are displayed at 2 frames/sec. Representative images from three independent biological experiments are shown. Video related to Fig. 6.

**Video 3.** **Impaired nuclear localization of separase in the absence of securin or Sak1.** The strains *pds1Δ HTB2-mCherry ESP1-eGFP MET3-CDC20* (Y2162) and *sak1Δ ESP1-eGFP MET3-CDC20* (Y2117) were arrested in metaphase and released into anaphase by Cdc20 depletion and re-addition. The cells were combined and deposited in concanavalin-coated chambers, with time-lapse images captured every 2 min using Airyscan2. The videos are presented at 2 frames/sec. Representative images of three independent biological experiments are included. Video related to Fig. 6.

Next, we investigated separase localization in cells synchronized in G1 phase using α-factor. In control cells, separase nuclear accumulation was observed on average 12 min before anaphase onset (Fig. 6, C−E and Video 4). In contrast, in *SAK1-E50K* mutant cells, separase entered the nucleus prematurely, on average 24 min before anaphase onset, compared with control cells. However, Esp1-eGFP nuclear levels declined similarly in late anaphase in both wild-type and *SAK1-E50K* cells (Fig. 6 C, dark green). These findings indicate that timely nuclear localization of separase depends on Sak1 activity.

**Video 4.** **Premature separase nuclear localization in *SAK1-E50K* cells.** The strains *HTB2-mCherry ESP1-eGFP MET3-CDC20* (Y2095) and *SAK1-E50K ESP1-eGFP MET3-CDC20* (Y2105) were arrested in metaphase and released into anaphase by Cdc20 depletion and re-addition. The cells were mixed and placed in concanavalin-coated chambers, and time-lapse images were captured using Airyscan2 at 2-min intervals. The videos are displayed at 2 frames/sec. Representative images of three independent biological experiments are shown. Video related to Fig. 6.

**Figure 7.**
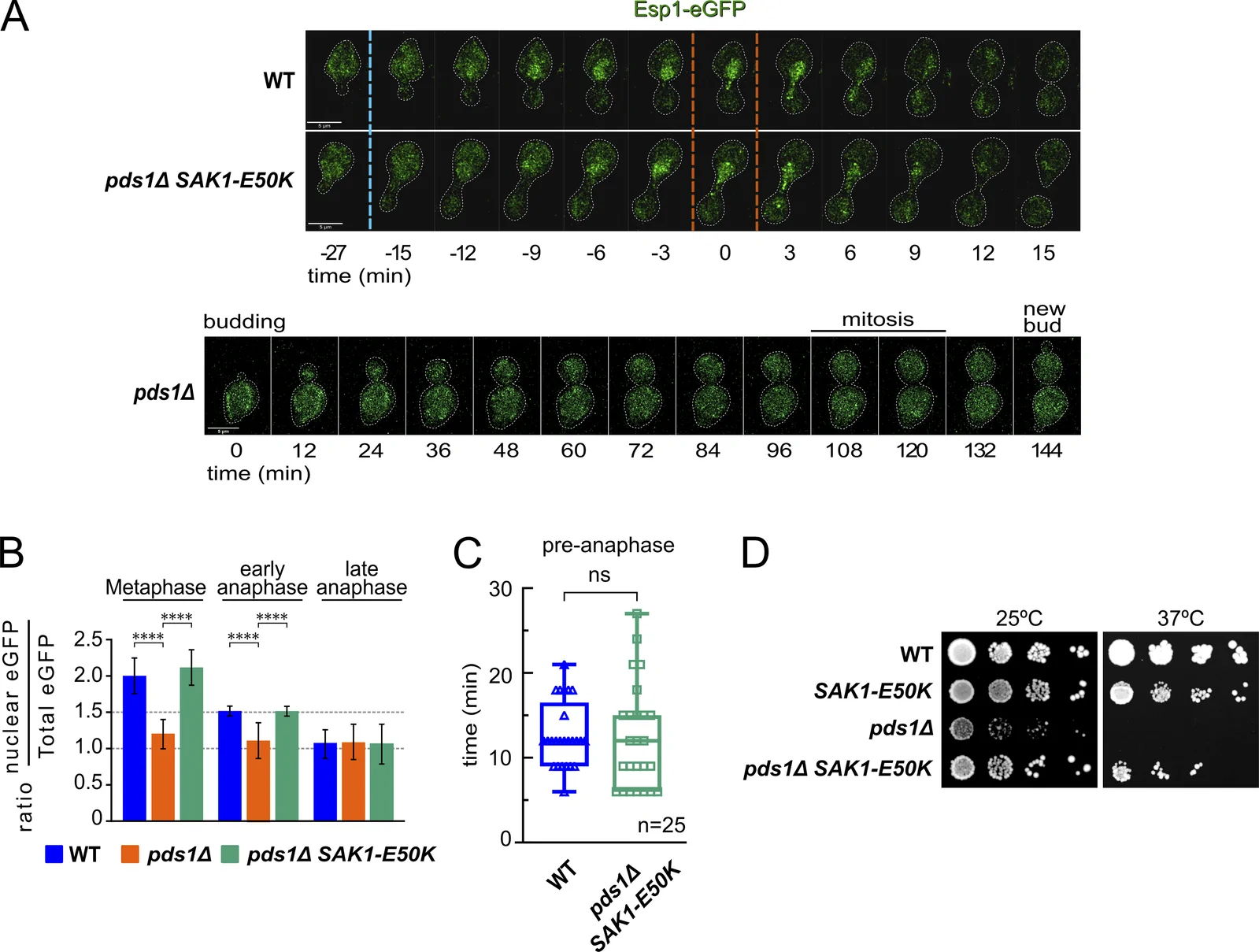
***SAK1-E50K* rescues separase nuclear localization in securin-deficient cells. (A–C)** *SAK1-E50K* mutation restores separase nuclear localization in the absence of securin. Strains *HTB2-mCherry ESP1-eGFP* (Y2188), *pds1Δ SAK1-E50K ESP1-eGFP* (Y2363), and *pds1Δ HTB2-mCherry ESP1-eGFP* (Y2362) were arrested in G1 with α-factor and released into a synchronous cell cycle. Representative images (A) and quantification of the Esp1-eGFP nuclear-to-cytoplasmic ratio (B) are shown. Means and SEMs from three independent biological experiments are shown. Box plot quantification of nuclear Esp1-eGFP signal before anaphase (C) is depicted. Scale bar, 5 μm. See also Video 5. **(D)** The *SAK1-E50K* mutation rescues the growth defects of *pds1Δ* cells. Serial dilutions of the indicated cells were spotted on YPD plates and grown at 25 and 37°C for 2–3 days (lower right panel). Images are representative of at least three independent biological experiments. Statistical significance: ns, nonsignificant; ****P < 0.0001. Source data are available for this figure: SourceData F7.

Strikingly, in the *sak1*Δ mutant cells, separase failed to accumulate in the nucleus throughout the entire cell cycle, mimicking the *pds1*Δ phenotype (Fig. S4 D). Together, our results demonstrate that both securin and Sak1 are required for separase nuclear localization. The *SAK1-E50K* mutation enhances separase binding, increases separase phosphorylation, and promotes greater nuclear retention. Additionally, *SAK1-E50K* accelerates separase nuclear entry, prolonging its presence in the nucleus during the cell cycle (Fig. 6 C). While these findings suggest that Sak1 actively contributes to separase nuclear localization, we cannot rule out the possibility that separase enters the nucleus solely via securin, with Sak1 phosphorylation functioning to retain separase within the nucleus. Notably, Esp1-eGFP still accumulated in the nucleus during mitosis in *pds1*Δ *SAK1-E50K* double mutants, similar to WT cells and in contrast to *pds1*Δ mutant cells (Fig. 7, A−C, Table S2, and Video 5). Moreover, the *SAK1-E50K* mutation rescued the growth defects observed in the *pds1*Δ mutant as well as its viability at 37°C (Fig. 7 D). These results suggest that *SAK1-E50K* promotes nuclear localization of separase independently of securin.

**Video 5.** **The *SAK1-E50K* mutation restores separase nuclear localization in the absence of securin**. The strains *HTB2-mCherry ESP1-eGFP* (Y2188) and *pds1Δ SAK1-E50K ESP1-eGFP* (Y2363) were arrested in G1 with α-factor and released into a synchronous cell cycle. The cells were combined and deposited in concanavalin-coated chambers, with time-lapse images acquired every 2 min using Airyscan2. The videos are presented at 2 frames/sec. Representative images of three independent biological experiments are shown. Video related to Fig. 7.

### Separase interactions with securin and Sak1

**Figure 8.**
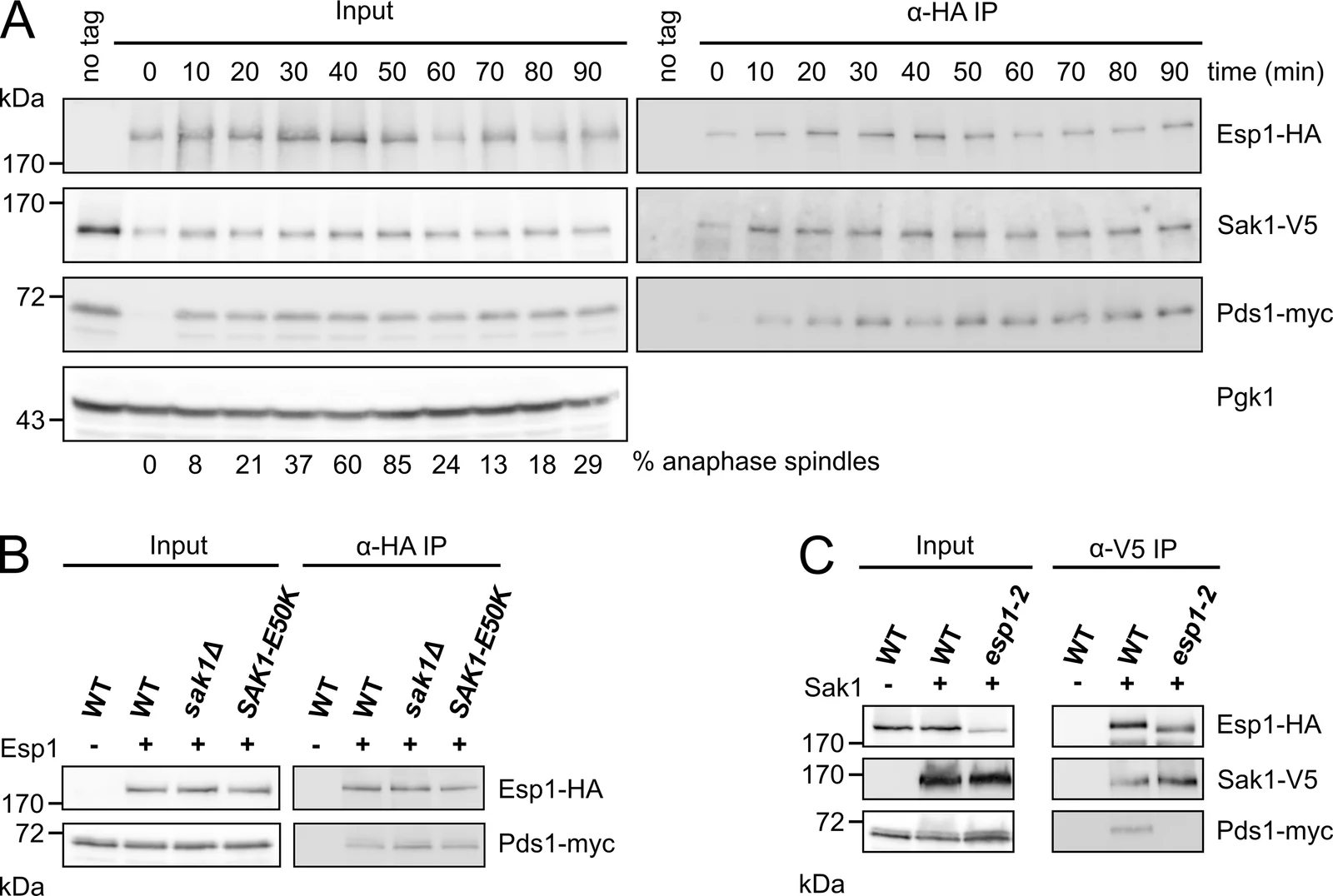
**Interactions between separase, securin, and Sak1. (A)** Separase and Sak1 interact independently of securin. Co-immunoprecipitation of separase and Sak1 was analyzed in protein extracts from the *ESP1-6HA SAK1-6V5 PDS1-9myc* (Y2295) strain. Strain *ESP1-6HA PDS1-9myc* (Y2127) without the V5 epitope was used as a negative control. Blots are representative of at least three independent biological experiments. **(B)** Separase and securin interact independently of Sak1. Securin co-immunoprecipitation with separase from extracts of strains *ESP1-6HA PDS1-9myc* (Y2127), Y2126 (as Y2127, but *SAK1-E50K*), and Y2408 (as Y2127, but *sak1Δ*) was analyzed. Strain *PDS1-9myc* (Y2294) without the HA epitope was used as a negative control. Images are representative of at least three independent biological experiments. **(C)** The interaction between securin and Sak1 depends on separase. Co-immunoprecipitation between securin and Sak1 was analyzed in protein extracts from strains *ESP1-6HA SAK1-6V5 PDS1-9myc* (Y2295) and *esp1-2-6HA SAK1-E50K PDS1-9myc* (Y2290) that were incubated at 32°C to inactivate the *esp1-2* allele for 3 h before protein extract preparation. Strain *ESP1-6HA PDS1-9myc* (Y2127), without the V5 epitope, was used as a negative control. Blots are representative of at least three independent biological experiments. Source data are available for this figure: SourceData F8.

Securin binds to and inhibits separase, acting as both a direct inhibitor and a pseudo-substrate that blocks the caspase-like domain of separase until anaphase onset (Luo and Tong, 2017; Yu et al., 2021). Sak1 interacts with separase and regulates its activity via phosphorylation. This raises the question of whether the Sak1-separase interaction is mediated by securin. To explore this, we performed co-immunoprecipitation experiments. First, we investigated whether the interaction between separase and Sak1 requires securin. We performed Sak1 co-immunoprecipitations in protein extracts obtained from cells synchronized in G1 phase using α-factor. Separase co-purified with Sak1 throughout the time course, both in the absence (0 min) and presence of securin (Fig. 8 A). These results indicate that Sak1 binds to separase independently of securin.

Next, we tested whether the interaction between securin and separase depends on Sak1 by performing co-immunoprecipitation analysis in wild-type and *sak1*Δ mutant strains. The absence of Sak1 did not affect the co-purification of securin and separase (Fig. 8 B), suggesting that securin binds to separase independently of Sak1.

Given the interactions among separase, securin, and Sak1, we hypothesized that Sak1 interacts with securin and that this interaction may be mediated by separase. To address this, we performed co-immunoprecipitation experiments between securin and Sak1 in protein extracts obtained from wild-type and *esp1-2* mutant strains, in which separase was inactivated at the restrictive temperature for the *esp1-2* allele. In WT cells, securin co-purified with Sak1 (Fig. 8 C). However, in *esp1-2* mutant cells, the co-purification of securin with Sak1 was significantly impaired, suggesting that Sak1 associates with securin in a separase-dependent manner. Notably, separase still co-purified with Sak1 in *esp1-2* mutant cells, indicating that the separase-Sak1 interaction remained unaffected despite separase inactivation.

**Figure 9.**
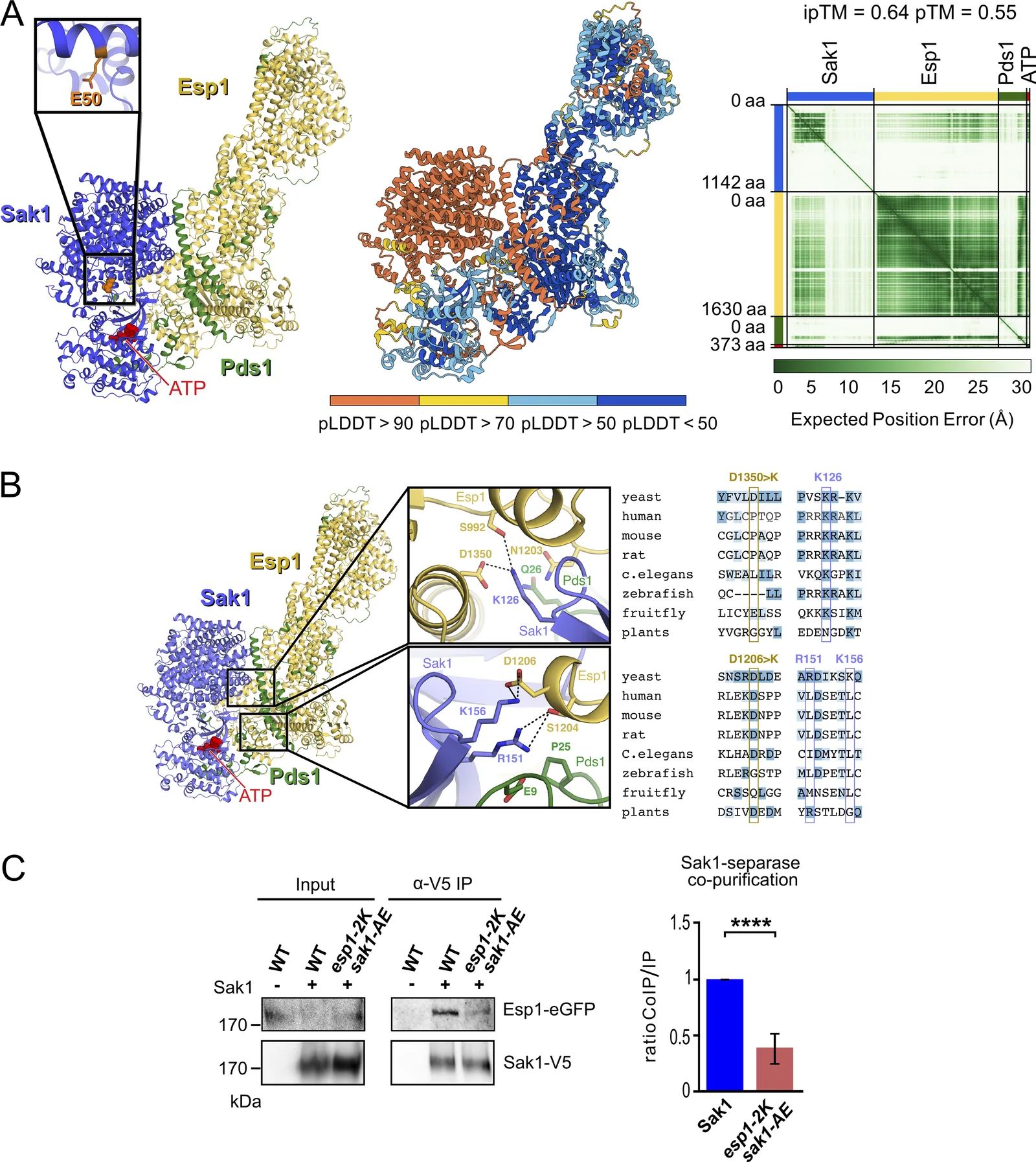
**Analysis of the Sak1–Esp1 interaction interface. (A)** Structural model of the predicted interaction between separase-securin and Sak1, generated by AlphaFold3. Yellow: separase; green: securin; blue: Sak1; red: ATP; orange: Sak1 residue E50. The predicted model is additionally colored according to the pLDDT score, and the predicted interaction between Sak1, separase, and securin was evaluated using the ipTM and pTM scores. **(B)** Detailed view of the Sak1–Esp1 interaction surface. Conservation of the residues involved in protein–protein interactions is shown across model organisms (right). Identical amino acids are indicated by dark blue shading and conserved residues by light blue shading. **(C)** Reduced Sak1–Esp1 interaction in *esp1-D1206K-D1350K sak1-R151A-K156E* (*esp1-2K sak1-AE*) mutant cells. Co-immunoprecipitation between separase and Sak1 was analyzed in protein extracts from strains *ESP1-eGFP SAK1-6V5* (Y2564) and *esp1-D1206K-D1350K*-*eGFP sak1-R151A-K156E*-*6V5* (Y2565). Strain *ESP1-eGFP* (Y2559), lacking the V5 epitope, was used as a negative control. Blots are representative of at least three independent biological experiments. Statistical significance: ****P < 0.0001. Source data are available for this figure: SourceData F9.

AlphaFold3 (Abramson et al., 2024) predicted a high-confidence interaction between separase and Sak1 (Fig. 9 A). Although Sak1-E50 is predicted to be solvent-exposed, structural analysis using PyMOL identified key residues at the predicted interaction surface (Fig. 9 B), specifically involving residues D1206 and D1350 in Esp1 and residues K126, R151, and K156 in Sak1. Sequence alignment of homologous proteins revealed partial conservation of these residues across evolution, suggesting a potential contribution to the yeast Esp1-Sak1 interaction. Charge-reversal substitutions in separase (D1206K and D1350K) and Sak1 (R151A and K156E) greatly reduced Sak1 co-purification in Esp1 pull-down assays (Fig. 9 C), supporting their contribution to the Esp1-Sak1 interaction.

**Figure 10.**
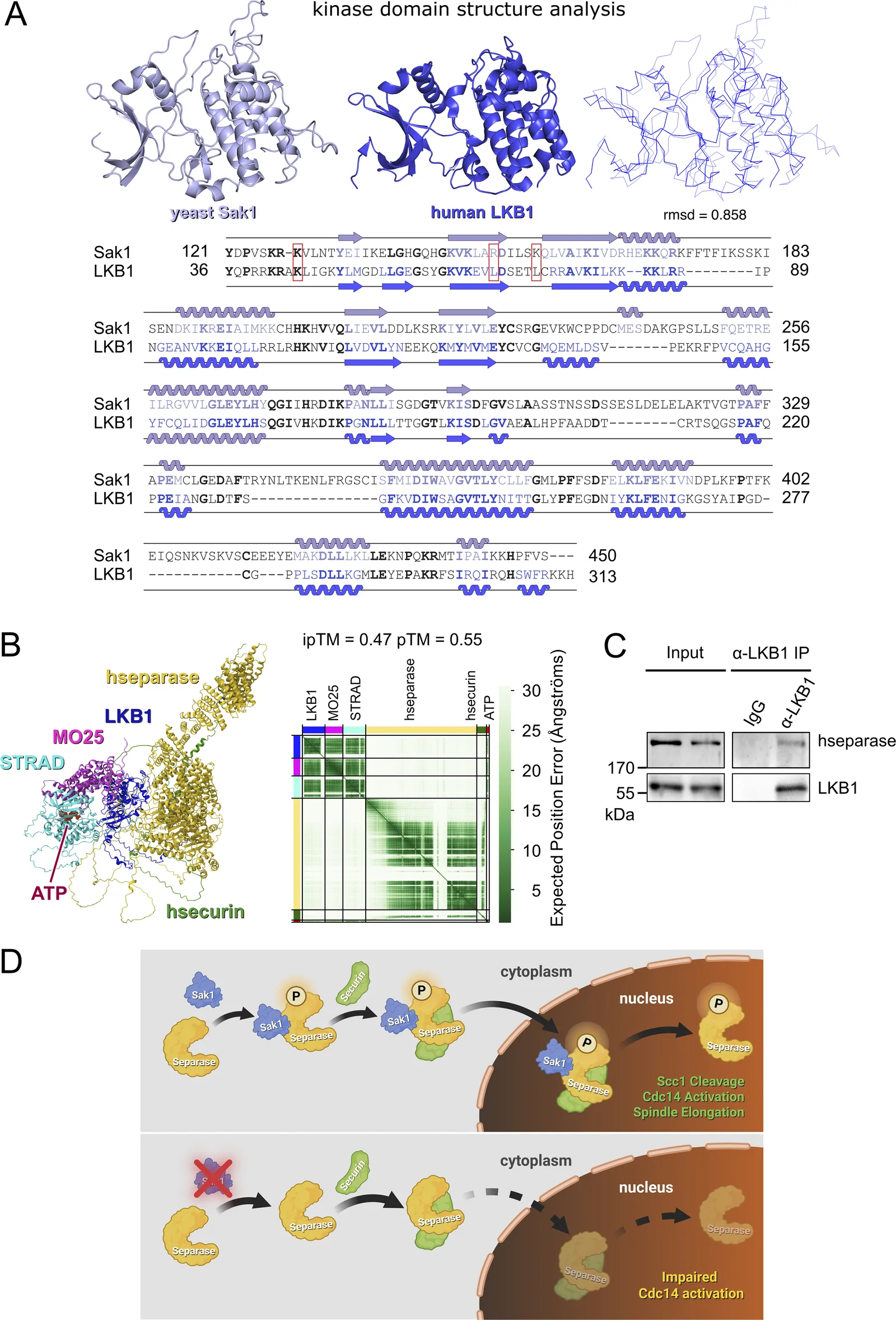
**The Sak1–Esp1 interaction is conserved in human cells. (A)** Structural superposition and sequence alignment of the kinase domains of Sak1 and LKB1, indicating the root-mean-square deviation (rmsd), secondary structural elements, and conserved residues (highlighted in bold). Red squares indicate the Sak1 residues mutated in Fig. 9 C. **(B)** Predicted interaction model between human separase, securin, and the LKB1–STRAD–MO25 complex generated by AlphaFold3, indicating the ipTM and pTM scores. **(C)** Interaction between LKB1 and human separase. LKB1 was immunopurified with an α-LKB1 antibody from the protein extract of HEK293T cells, and co-purification of human separase was analyzed. Blots are representative of three independent biological replicates. **(D)** Proposed model for Sak1 regulation of separase. Sak1 phosphorylates separase, promoting its nuclear accumulation and activation. In the absence of Sak1, separase translocation to the nucleus is reduced, impairing Cdc14 activation. Sak1-dependent phosphorylation also activates separase, providing an additional activation mechanism. Source data are available for this figure: SourceData F10.

Although the kinase domains of Sak1 and its human ortholog LKB1 share only 36.7% sequence identity, they exhibit highly similar structural features (Fig. 10 A). LKB1 is regulated through the formation of the LKB1–STRAD–MO25 complex, which modulates its activity, subcellular localization, and substrate specificity (Boudeau et al., 2004). AlphaFold3 additionally predicted a potential interaction between human separase and the LKB1–STRAD–MO25 complex (Fig. 10 B). Although the predicted interaction score falls below the threshold typically considered reliable for robust structural inference, it nonetheless suggests the possibility of a conserved association. Consistent with this prediction, we observed co-purification of human separase upon LKB1 immunoprecipitation (Fig. 10 C), confirming a physical association between these two proteins. Taken together, these findings support the hypothesis that the separase-kinase regulatory interaction identified in yeast may also be conserved in humans.

In summary, these results suggest that Sak1 plays a crucial role in regulating separase localization and activity, independent of securin. Furthermore, the separase-dependent interaction between Sak1 and securin highlights a potential mechanism by which Sak1 modulates separase function during mitotic progression.

## Discussion

A crucial process during mitosis is the correct segregation of genetic material into two daughter cells. This highly regulated process is initiated in eukaryotic cells by separase, a caspase-like protease that cleaves the Scc1 subunit of the cohesin complex, thereby enabling sister chromatid separation at anaphase onset. Beyond its proteolytic role, separase activates Cdc14 phosphatase, promotes mitotic spindle elongation, and regulates rDNA condensation and resolution. The identification of the *SAK1-E50K* mutation as a suppressor of the temperature-sensitive separase allele revealed relevant genetic interactions between *SAK1* and *ESP1*. Our data suggest that Sak1 may act as part of the FEAR pathway, phosphorylating separase and regulating its nuclear localization, thereby providing new insights into the intricate control of mitotic progression. Importantly, the observation that human separase physically associates with LKB1 supports the evolutionary conservation of this regulatory axis. Given the link between separase overexpression and tumorigenesis, investigating the conservation and functional relevance of this pathway in higher organisms, particularly its relationship with the LKB1 tumor suppressor, may reveal novel therapeutic targets for cancer.

### Regulation of separase localization and activation by Sak1

Our model integrates well-established aspects of separase regulation by securin while addressing an unresolved question: How does separase localize to the nucleus during mitosis when securin is absent? In *pds1Δ* mutants, separase remains cell cycle–regulated and localizes to the nucleus, albeit at lower levels, which may explain why *pds1Δ* cells remain viable in *S. cerevisiae*. We propose that Sak1 regulates separase localization independently of securin, contributing to separase accumulation in the nucleus during mitosis (Fig. 10 D). Sak1 phosphorylation not only promotes separase nuclear localization but also activates separase, providing an alternative mechanism of separase activation.

Although it remains unclear whether Sak1 phosphorylates separase before or after its nuclear entry, our data suggest that phosphorylation occurs in the cytoplasm, thereby facilitating both nuclear import and activation. Supporting this hypothesis, *SAK1-E50K* restores separase nuclear localization and rescues the growth defects of the *pds1Δ* mutant, suggesting that the mutation enhances separase stabilization or its interaction with securin.

Even though the E50 lies outside Sak1’s predicted kinase domain and the mapped Esp1-Sak1 interface, gain-of-function mutations frequently arise from allosteric or regulatory effects rather than from changes in catalytic or binding residues. Charge inversion at residue E50 may modulate protein conformation or dynamics, indirectly enhancing Sak1 activity toward separase.

### Sak1 as a positive regulator of separase and Cdc14

Despite being a gain-of-function allele, *SAK1-E50K* alone does not accelerate mitotic progression, likely due to stringent regulatory mechanisms controlling separase activity. Although *SAK1-E50K* may enhance separase function, additional inhibitory factors such as securin prevent excessive or premature activation. A phenotype only emerges when this regulation is disrupted, as observed in the *esp1-2* mutant. This is consistent with the observation that neither Cdc14 overexpression (Visintin et al., 1998; Higuchi and Uhlmann, 2005) nor PP2A-Cdc55 inactivation (Queralt et al., 2006; Baro et al., 2013) accelerates mitosis under unperturbed conditions in budding yeast.

In contrast, combining *SAK1-E50K* with ectopic separase expression (*GAL1-ESP1 SAK1-E50K*, Fig. 4 B) accelerated anaphase and mitotic exit. This suggests that increased separase activity, when released from securin inhibition and activated by Sak1 phosphorylation, is sufficient to drive mitotic exit.

### Sak1 functions as a component of the FEAR pathway

Multiple lines of evidence support the function of Sak1 within the FEAR pathway. Although delayed Cdc14 release in the absence of Sak1 suggests direct activation of Cdc14, ectopic *SAK1* expression in metaphase-arrested cells did not trigger Cdc14 nucleolar release (Fig. S4 E). Moreover, although overexpression of FEAR components, such as *SPO12* or *ZDS1*, rescues *cdc15-2* lethality (Queralt and Uhlmann, 2008; Rossio et al., 2013), *GAL1-SAK1 cdc15-2* cells are not viable at semi-restrictive temperatures (Fig. S4 F). This suggests that while Sak1 is involved in the FEAR pathway, it does not regulate Cdc14 activation independently of separase.

### Sak1’s role in mitosis is independent of the Snf1/AMP pathway

Sak1 regulates separase independently of its known role in Snf1 activation (Fig. 1 D and Fig. S2). This finding is consistent with that of its mammalian ortholog, LKB1, which has additional roles beyond AMPK activation, including lipid metabolism regulation during exercise (Jeppesen et al., 2013) and maintenance of genomic integrity during liver regeneration (Maillet et al., 2018).

While Sak1’s phosphorylation of Snf1 has been well studied, with T210 as the primary phosphorylation site (Nath et al., 2003; Elbing et al., 2006), the phosphorylation sites on Pso2 remain unidentified (Munari et al., 2014). A consensus phosphorylation sequence for Sak1 has not been established, limiting the prediction of potential phosphorylation sites in separase. To address this, we employed mass spectrometry to identify the residues phosphorylated by Sak1. However, the residues identified as phosphorylated by Sak1 based on Phos-tag gel analysis have so far eluded detection by mass spectrometry.

Given that separase overexpression is linked to aneuploidy, tumorigenesis, and poor prognosis in various cancers (Zhang et al., 2008; Meyer et al., 2009; Finetti et al., 2014; Mukherjee et al., 2014), investigating whether this regulation is conserved in human cells, particularly its relationship with LKB1, may yield valuable insights into mitotic control and provide promising targets for cancer therapy.

## Materials and methods

### Plasmid construction and yeast culture conditions

The budding yeast strains used in this study were derived from the W303 genetic background, and their descriptions are presented in Table S3. Cells were cultured in rich YP medium (1% yeast extract, 2% peptone) supplemented with the appropriate carbon source: YPD (2% glucose), YPRAF (2% raffinose), YPGAL (2% galactose and 2% raffinose), or minimal medium (0.67% yeast nitrogen base, 40 μg/ml of required amino acids and nucleotides for auxotrophy selection, and 2% of the corresponding carbon source).

Cell synchronization in the G1 phase using the mating pheromone α-factor and mitotic synchronization via Cdc20 depletion were performed as previously described (Monje-Casas and Queralt, 2017). For G1 arrest, α-factor was added to a final concentration of 7.5 µg/ml. The cells were examined using phase-contrast microscopy until at least 90% were unbudded. To release the cells from G1 arrest, the cells were filtered, washed, and transferred to fresh medium.

For synchronization at the metaphase-to-anaphase transition by depleting Cdc20, the endogenous *CDC20* promoter was replaced with either the galactose-inducible *GAL1* promoter or the methionine-repressible *MET3* promoter. In *GAL1-CDC20* experiments, cells were cultured in YPGAL medium, filtered under sterile conditions, washed with YP, resuspended in YPRAF, and incubated with agitation for 3–4 h. Metaphase arrest was monitored until at least 90% of the cells exhibited large buds (similar size between mother and daughter cells). Synchronous anaphase release was achieved by adding 2% galactose directly to the culture medium. In *MET3-CDC20*-experiments, cells were grown in minimal medium lacking methionine, pelleted by centrifugation, and resuspended in medium containing methionine at a final concentration of 2 mM. Cells were incubated with agitation for 3–4 h, and metaphase arrest was confirmed when > 90% of the cells displayed large buds. To release the cells synchronously into mitosis, the cells were filtered, washed with sterile distilled water, and resuspended in a minimal medium lacking methionine.

Inactivation of the temperature-sensitive separase allele *esp1-2* in exponential phase cultures was performed at 32°C with agitation for 4 h. Inactivation in metaphase-arrested cells was achieved by depleting Cdc20 for 2 h, followed by incubation at 32°C for an additional 3 h before release from arrest, also at 32°C.

Ectopic expression of *SAK1* and *ESP1* was performed using a promoter under the control of the galactose-inducible *GAL1* promoter. Cells were grown in minimal medium containing 2% raffinose, arrested in metaphase by Cdc20 depletion, and 2% galactose was directly added to the culture after metaphase arrest.

Endogenous genes were tagged or deleted using homologous recombination by transformation with PCR products (Knop et al., 1999; Wach et al., 1994). The p*SAK1-TAP* 2-µm plasmid was kindly provided by Dr. Martin C. Schmidt, University of Pittsburgh School of Medicine, Pittsburgh, PA, USA (Elbing et al., 2006). To generate the p*GAL1-SAK1-TAP* plasmid, the amplified DNA fragment corresponding to the *SAK1-TAP* sequence was introduced into the YIp204*GAL1* vector linearized with BamHI via DNA assembly. The resulting plasmid was linearized with BstXI before yeast transformation for integration into the *TRP1* locus.

### HEK293T cell culture conditions

HEK293T (Merck) cells were maintained in Dulbecco’s Modified Eagle Medium (DMEM; Corning) supplemented with 10% fetal bovine serum (FBS; Capricorn) and 1% penicillin/streptomycin (Biowest) at 37°C, 95% humidity, and 5% CO_2_.

### Isolation of the suppressor mutants of the *esp1-2* mutant allele

One million cells of the *esp1-2* mutants (Y500 and Y582) were spread onto YPD plates and incubated at 30°C, a restrictive temperature. None of the *esp1-2* strains with suppressor mutations obtained a 30°C, grew at 35°C, or 37°C. Under these conditions, only spontaneous suppressor mutants formed colonies. After 4–5 days, distinct colonies appeared (24 for the *esp1-2* Mat a mutant and 30 for the *esp1-2* Mat alpha mutant). These colonies were re-streaked, and the suppressor phenotype was analyzed by spotting serial dilutions (Fig. S1 A).

### Whole-cell protein extraction and immunoblotting

Protein extracts were prepared using the TCA method. Cells were harvested by centrifugation, immediately resuspended in cold 20% TCA, and fixed overnight at 4°C. After fixation, cells were washed with 1 M Tris base and resuspended in loading buffer (100 mM Tris-HCl, pH 6.8, 0.2 M DTT, 4% glycerol, and 0.002% bromophenol blue) preheated to 95°C. One volume of glass beads (425–600 µm in diameter) was added, and cells were mechanically lysed using a Precellys 24 homogenizer (Bertin technologies) for 6 cycles of 30 s at 5,000 rpm.

For western blotting analysis, samples were boiled at 95°C for 5 min and clarified by centrifugation at 17,000 rcf for 5 min before loading onto protein gels. Protein samples were resolved by SDS-PAGE gels using 6, 8, 10, or 12% acrylamide gels, depending on the molecular weight of the proteins of interest, and transferred to nitrocellulose membranes. The membranes were blocked with PBS, 0.02% Tween-20 (PBS-Tween) containing 5% milk. Membranes were incubated with the primary antibody for 2 h or overnight, followed by incubation with the secondary antibody for 60 min. After each antibody incubation, the membranes were washed three times with PBS-Tween. For chemiluminescent detection, membranes were treated with the SuperSignal Pico Plus detection reagent (Thermo Fisher Scientific) or ECL Select (Cytiva), according to the manufacturer’s instructions, and proteins were visualized using the Amersham Imager LAS 4000 (Cytiva) or Imager 600 (Cytiva).

To analyze the phosphorylated isoforms of separase, Phos-tag gels (6% acrylamide) containing 100 μM Phos-tag (Wako) and 200 μM MnCl_2_ were used. The protein electrophoresis was conducted at 20 mA for 5 h. Before protein transfer, the gels were washed twice with 1 mM EDTA for 10 min to remove manganese. Proteins were then transferred at 400 mA for 2 h under cold conditions. Protein quantification was performed using the ImageJ software (RRID:SCR_003070). For the phosphorylation analysis of Esp1, lanes were selected using the Gel Analyzer tool. Equal-width rectangular selections were drawn around each lane and added sequentially using the Analyze > Gels > Select First Lane and Select Next Lane commands. Densitometric profiles were then generated using Analyze > Gels > Plot Lanes. Background correction was performed using the standard ImageJ Gel Analyzer procedure. For each lane profile, the baseline was manually defined by drawing straight lines between the edges of the peaks corresponding to the Esp1 signals, thereby subtracting the local background signal. The area of each peak above the baseline was measured using the Gel Analyzer wand tool. The proportion of phosphorylated Esp1 was calculated as the area of the phosphorylated isoform signal (low electrophoretic mobility band) divided by the sum of the areas of the phosphorylated and dephosphorylated isoform signals (low- and high-mobility bands, respectively).

The following antibodies were used for western blotting: α-HA clone 12CA5 (Cat# 11666606001; Roche, RRID:AB_514506), α-myc clone 9E10 (Cat# MMS-150P-1000; Covance, RRID:AB_291322), α-FLAG clone M2 (Cat# F1804; Sigma-Aldrich, RRID:AB_262044), α-Pk clone SV5-Pk1 (Cat# MCA1360; Bio-Rad, RRID:AB_322378), α-Clb2 (y-180) sc-9071 (Cat# sc-9071; Santa Cruz Biotechnology, RRID:AB_667962), α-tubulin clone YOL1/34 (Cat# MCA78G; Bio-Rad, RRID: AB_325005), α-Pgk1 (Cat# 459250; Thermo Fisher Scientific, RRID: AB_2532235), α-HA clone F7 (Cat# sc-7392 HRP; Santa Cruz Biotechnology, RRID: AB_2894930), α-Snf1 40H9 (Cat# 2535; Cell Signaling Technology, RRID: AB_331250), α-PAP (Cat# P1291; Sigma-Aldrich, RRID: AB_1079562), α-LKB1 (Cat# 10746-1-AP; Proteintech, RRID:AB_2271311), and α-hSeparase (Cat#NB100439SS; Novus Biological, RRID:AB_3740116). The secondary antibodies used were α-mouse-HRP (Cat# NXA931; Cytiva, RRID: AB_772209), α-rabbit-HRP (Cat# NA934, Cytiva, RRID:AB_772206), and α-goat-HRP (Cat# sc-2020, Santa Cruz Biotechnology, RRID:AB_631728).

### Co-immunoprecipitation of proteins

For co-immunoprecipitation assays, 10^8^ exponentially growing cells were harvested, washed with 1x PBS, and resuspended in lysis buffer (50 mM Hepes-KOH, pH 7.5, 70 mM KOAc, 5 mM Mg(OAc)_2_, 10% glycerol, 0.1% Triton X-100, 1 mM DTT, 8 μg/ml protease inhibitors [leupeptin, pepstatin, and aprotinin], 1 mM PMSF, 1 tablet of complete protease inhibitor without EDTA [Roche], 5 mM β-glycerophosphate and 4 μg/ml NaF phosphatase inhibitors, and 1 tablet of PhosStop [Roche]). Glass beads (425–600 μm) were added at a 1:1 volume ratio, and the cells were mechanically lysed using a Precellys 24 homogenizer (Bertin Technologies) for 6 cycles of 10 s at 5,000 rpm. Protein extracts were clarified by centrifugation at 17,000 rcf for 10 min and incubated on ice with the primary antibody (α-HA clone F7 (Cat# sc-7392 HRP; Santa Cruz Biotechnology, RRID: AB_2894930) or α-Pk clone SV5-Pk1 (Cat# MCA1360, Bio-Rad, RRID: AB_322378) for at least 1 h.

Immunocomplexes were captured using Dynabeads conjugated with protein A (Cat# 10002D; Life Technologies) and incubated for 1 h at 4°C with rotation. The immunoprecipitated proteins were washed with lysis buffer containing increasing concentrations of KOAc (100, 120, and 150 mM), followed by a final wash with lysis buffer containing 60 mM NaOAc. Proteins were eluted with 2X SDS-PAGE loading buffer and frozen at −20°C until further analysis by western blotting.

For the co-immunoprecipitation of the Sak1-TAP protein, clarified protein extracts were incubated directly with IgG-Sepharose resin (Cat# 17096901; Life Technologies) for 1 h, washed with lysis buffer, and eluted with 2X SDS-PAGE loading buffer.

For co-immunoprecipitation in human cells, HEK293T cells were seeded in 150-mm dishes and grown to ∼70% confluence. For each experimental replicate, three plates were processed for LKB1 immunoprecipitation and three plates for rabbit IgG negative control (Cat# 026102; Life Technologies). Cells were washed twice with ice-cold 1x PBS and lysed directly on the plate using 1.5 ml of HEK lysis buffer (50 mM Tris-HCl, pH 7.5, 150 mM NaCl, 0.3% NP-40, 1 mM EDTA, and 10% glycerol) supplemented with protease and phosphatase inhibitors (1 mM PMSF, 1x Complete protease inhibitor without EDTA [Roche], and 1x PhosStop [Roche]). The plates were gently tilted and incubated on ice for 15 min. Lysates were transferred to pre-chilled tubes, incubated on ice for an additional 10 min, and clarified by centrifugation at 15,000 rcf for 15 min at 4°C. Clarified lysates were precleared by incubation with 60 μl of Protein A-Dynabeads (Life Technologies) for 45 min at 4°C. After preclearing, the protein extracts were incubated with 4 μl of anti-LKB1 antibody (Cat# 10746-1-AP; Proteintech, RRID:AB_2271311) or rabbit IgG as a negative control for 2 h at 4°C with rotation. Immunocomplexes were then captured by adding 80 μl Protein A-Dynabeads (Life Technologies) and incubating for 1 h at 4°C with rotation. The immunoprecipitated proteins were washed three times with lysis buffer. For elution, 1x SDS-PAGE loading buffer was added, and the samples were incubated at 70°C for 10 min. Eluates were transferred to fresh tubes and stored at −80°C until further analysis by western blot.

### Purification of Esp1-TEV

The purification of Esp1 protein was carried out by overexpressing the *GAL1-FLAG-ESP1-CBP-TEV-CBD* construct (CBP, chitin-binding protein, CBD: chitin-binding domain) in yeast cultures. The cells were grown in rich media supplemented with raffinose, and galactose was added to induce expression of *FLAG-ESP1-CBP-TEV-CBD* for 4 h. Cells were then harvested and resuspended in cold EBX buffer (50 mM Hepes/NaOH, pH 7.5, 100 mM KCl, 1% Triton X-100, 2.5 mM MgCl_2_, and 1 mM DTT) containing phosphatase and protease inhibitors: 8 μg/ml Aprotinin, 8 μg/ml Pepstatin, 8 μg/ml Leupeptin, 4 mM PMSF, and 1X Complete EDTA free (Roche).

For mechanical lysis, an equal volume of glass beads (425–600 μm) was added, and the cells were lysed using a Pulverisette 6 homogenizer (Fritsch) with 12 pulses of 1 min at 500 rpm. The lysate was then centrifuged at 1,449 rcf for 1 min to remove the glass beads, followed by clarification of the supernatant at 17,000 rcf for 1 h at 4°C.

The clarified extract was incubated with chitin resin (New England Biolabs) for 2 h with rotation at 4°C. Following binding, the chitin resin was washed with cold EBX buffer using a Mobitec purification column. To elute the bound protein, TEV protease was added and incubated for 4 h with rotation at 4°C. After incubation, the eluate was collected, glycerol was added to a final concentration of 10%, and the sample was frozen at −80°C. Protein purity and process yield were assessed by protein electrophoresis with Coomassie blue staining and western blotting, respectively.

### Immunoprecipitation of Sak1 and kinase assay

Sak1 protein was immunoprecipitated from protein extracts of cells endogenously expressing *SAK1* tagged with the V5 epitope, following our established protocol for co-immunoprecipitation. To prevent endogenous separase or any other interacting proteins from co-purifying with Sak1, the detergent concentration was increased to 1% Triton X-100. Under these conditions, 15–30 pg of Sak1 protein was immunopurified. An additional wash step was performed using kinase buffer (20 mM HEPES pH 7, 0.5 mM EDTA, 5 mM MgCl_2_, and 0.5 mM DTT).

For the radioactive kinase assay, the immunoprecipitated Sak1 protein was incubated with 20 μl of reaction mixture containing kinase buffer, 6–15 ng of FLAG-Esp1-CBP purified protein, 50 µM cold ATP, and 1 Ci/μl γ-ATP-^32^P. The reactions were incubated at 30°C for 20 min and then terminated by adding 2x SDS-PAGE loading buffer. The incorporation of ^32^P by separase was detected using a PhosphoImager FLA-500 (Fujifilm) after resolving the proteins by electrophoresis and transferring them to a nitrocellulose membrane. Quantification of the ^32^P signal was performed using ImageJ (RRID:SCR_003070) software. Esp1 and Sak1 proteins were detected by Western blotting and quantified using ImageJ (RRID:SCR_003070). The kinase activity of Sak1 on separase was calculated by normalizing the phosphorylated separase signal relative to the amounts of Sak1 and Esp1.

For the non-radioactive kinase assay, immunoprecipitated Sak1 was used as the kinase source and incubated in a 30 μl reaction mixture containing kinase buffer, 6–15 ng of purified FLAG-Esp1-CBP protein, and 400 µM ATP. Reactions were incubated at 30°C for 20 min and terminated by the addition of 2x SDS-PAGE loading buffer. Phosphoproteins were detected by ProQ diamond staining (Life Technologies) after electrophoretic separation, and images were acquired using an iBright FL1500 imaging system (Thermo Fisher Scientific). Signal quantification was performed using the ImageJ (RRID:SCR_003070) software. Total Esp1 protein was detected by whole-protein staining with colloidal Coomassie (Merck), whereas Sak1 was detected by western blotting. Both proteins were quantified using ImageJ software (RRID:SCR_003070). Sak1 kinase activity toward separase was calculated by normalizing the phosphorylated Esp1 signal to the amounts of Sak1 and Esp1.

### *In situ* immunofluorescence

Approximately 10^8^ cells were fixed with 3.7% formaldehyde in phosphate buffer, pH 6.4 (100 mM potassium phosphate, pH 6.4, and 0.5 mM MgCl_2_) for 1 h at 25°C or overnight at 4°C. The fixed cells were washed with phosphate buffer (pH 6.4; without formaldehyde), followed by washing with phosphate buffer (pH 7.4; 120 mM potassium phosphate, pH 7.4, 0.5 mM MgCl_2_, and 1.2 M sorbitol).

To obtain spheroplasts, cells were incubated with phosphate buffer (pH 7.4) containing 0.1 mg/ml zymolyase 100T and 0.2% β-mercaptoethanol for 30–60 min at 30°C. The spheroplasts were then washed with phosphate buffer (pH 7.4) to remove residual Zymolyase and mounted onto multi-well slides coated with polylysine.

Spheroplasts were blocked with 1x PBS containing 1% BSA (PBS-BSA) for 30 min, and then incubated with the primary and secondary antibodies diluted in the blocking solution (PBS-BSA) for 1 h. After each antibody incubation, the samples were washed five times with PBS-BSA. A mounting medium with DAPI (VECTASHIELD) was added for nuclear visualization. Cells were visualized, and images were acquired using an inverted epifluorescence microscope, Zeiss Axio Observer Z1, with an HXP 12 C fluorescence lamp and a Plan-Apo 63× N.A 1.4 objective controlled by ZEN 2011 Software (RRID:SCR_013672), or a Leica DM6 B Thunder vertical microscope with an LED 5 fluorescence lamp and a Plan-APO CS 63× N.A 1.3 objective controlled by LAS X (RRID:SCR_013673). The length of the mitotic spindle was measured using the quantitative analysis tools of ZEN 2011 software (Zeiss).

The following antibodies were used: primary antibodies: α-HA clone 16B12 (Cat# MMS-101R; Covance, RRID: AB_291262), α-tubulin YOL1/34 (Cat# MCA78G, Bio-Rad, RRID: AB_325005), and α-Cdc14 (Cat# sc-12045, Santa Cruz Biotechnology, RRID: AB_671801); secondary antibodies included anti-goat conjugated with Cy3 (Cat# 705-166-147, Jackson ImmunoResearch Labs, RRID: AB_2340413), anti-mouse conjugated with Cy3 (Cat# PA43002, Cytiva, RRID: AB_772235), and anti-rabbit conjugated with FITC (Cat# AP189F, Millipore, RRID: AB_11212459).

### *In vivo* fluorescence microscopy

To quantify the subcellular localization of Cdc14 *in vivo*, synchronized cells were prepared for time-lapse microscopy by depositing them in microscopy chambers (Labtek) coated with 1 mg/ml concanavalin A in 1x PBS and incubated in minimal media at 25°C. Images were captured every 3 min using an inverted epifluorescence microscope (Leica DMI 8) with a 100× oil objective (HC PL APO 100×/1.40 OIL CS2) equipped with a DFC9000 GT VSC-07924 camera, controlled by LAS X Life Science Microscope software (version 3.5.5, RRID:SCR_013673). The fluorescence signals of Cdc14-eGFP and Net1-mCherry were quantified using ImageJ (RRID:SCR_003070) software. The CV between Cdc14 and Net1 (CVCdc14/Net1) was calculated as described previously (Calabria et al., 2012; Neurohr and Mendoza, 2017).

To determine the subcellular localization of separase, cells were incubated in minimal medium at 25°C and imaged using a ZEISS LSM 980 confocal microscope equipped with an Airyscan 2 detector, operated in Multiplex SR-4Y mode controlled by Zen Blue software (RRID:SCR_013672). Images were acquired with a 63× oil objective (PL APO 63×/1.40 oil) using the manufacturer’s recommended settings for high-sensitivity detection of low-abundance fluorescent signals: Fast Airyscan Sheppard Sum SR-4Y: 3.8 scaling (per pixel) 0.0043 μm × 0.0043 μm × 0.500 μm, image size (pixels) 3,152 × 3,152, bit depth 16 bit, scan mode frame, scan zoom 1.0, pixel time 0.33 μs, frame time 1.95 s, LSM scan speed 7, scan direction bidirectional. Track1 (Htb2-mCherry): Pinhole 5.06 AU/313 μm, laser wavelength 561 nm: 3% (equivalent 1% in confocal mode), excitation wavelength 587, detection wavelength 300–735, detector type GaAsP-PMT, detector Gain 850 V. Track2 (Esp1-GFP): Pinhole 5.50 AU/272 μm, laser wavelength 488 nm: 9% (equivalent 3% in confocal mode), excitation wavelength 488, detection wavelength 495–550, detector type GaAsP-PMT, detector Gain 890 V.

**Figure S5.**
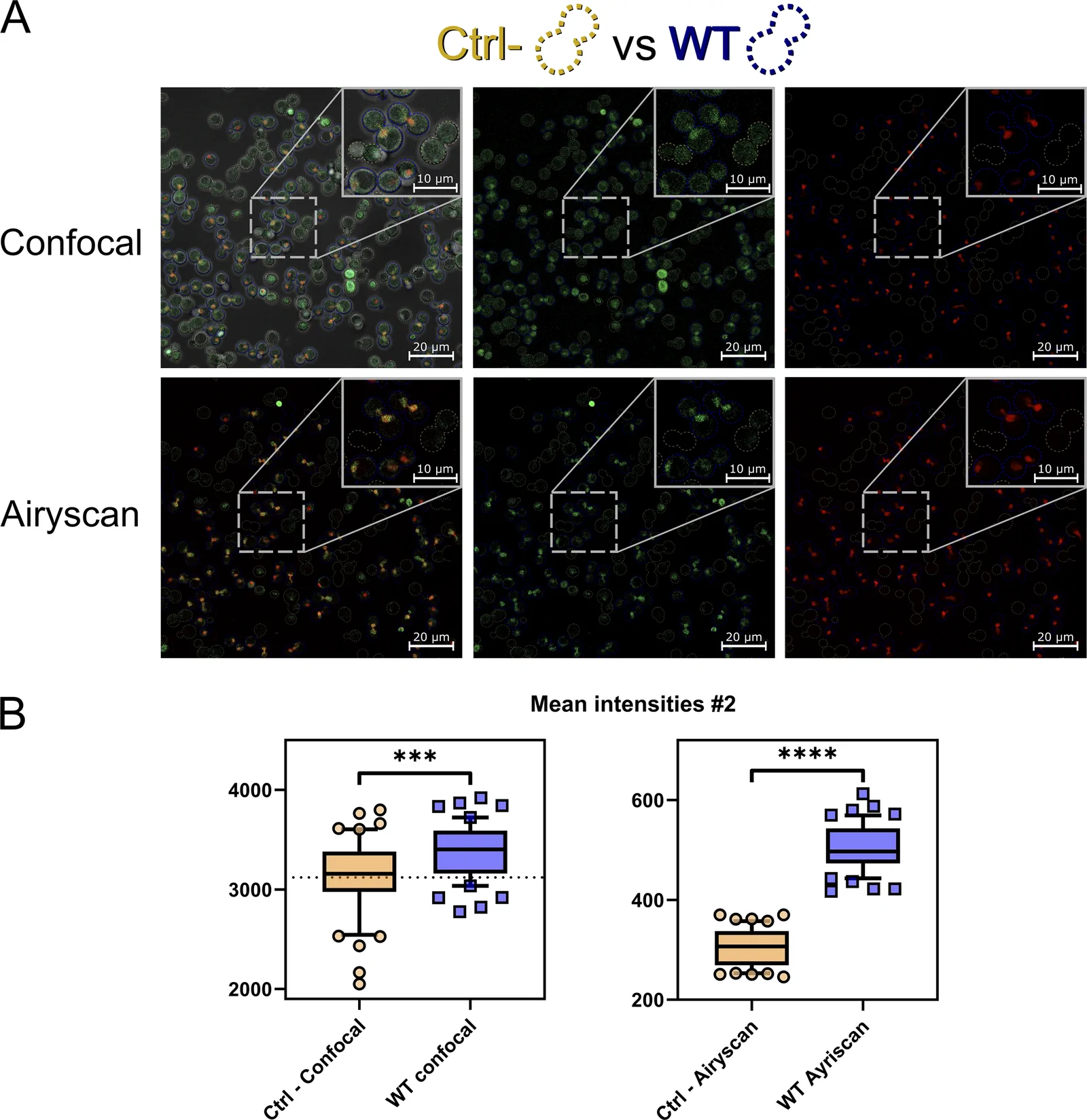
**Comparison of Esp1-eGFP and Htb2-mCherry signals in confocal and Airyscan2 imaging modes. (A)** Fluorescent signals were compared between a negative control strain, *MET3-CDC20* (Y1056), and the experimental strain *HTB2-mCherry ESP1-eGFP MET3-CDC20* (Y2095), which were arrested in metaphase by Cdc20 depletion. The same cells were imaged using both confocal and Airyscan2 modes. Representative images are shown. Scale bar, 10 μm. An enhanced signal-to-noise ratio in the Airyscan2 mode was evident. See also Video 6. **(B)** Quantification of Esp1-eGFP mean fluorescence intensity from A is depicted. Airyscan2 imaging revealed a greater distinction in signal intensity compared with the negative control, highlighting improved sensitivity. Statistical significance: ***P < 0.001; ****P < 0.0001.

All imaging parameters were kept constant between the experimental and control conditions. Negative controls (non-eGFP–expressing samples) were acquired under identical settings to demonstrate that the signals observed in the Esp1-eGFP samples were specific and above the background (Fig. S5, Table S4, and Video 6). The cytoplasmic and nuclear signals of Esp1-eGFP were quantified to calculate the nuclear-to-cytosolic signal ratio using the IA-based software, Arivis Pro. A deep-learning instance model (object-based) was trained using the Htb2-mCherry (nuclear) and Esp1-eGFP (cellular) fluorescence signals to segment nuclei and whole cells, respectively. The resulting “nuclei” and “cell” objects were associated with the Object Compartments operator. To track cells over time, the “cell compartments” were linked across time points using the tracking operator with the Brownian motion model. Subsequently, the cytoplasmic regions were defined by subtracting nuclear segmentation from whole-cell segmentation using the Object Math operator. Quantitative measurements, including maximum intensity, mean intensity, sum intensity, SD of intensity, and signal-to-noise ratio (calculated as mean/SD), were extracted from the Object Table. For time-lapse experiments involving mixed strains labeled with distinct fluorophores, the Object Filter operator was applied using fluorescence intensity thresholds to distinguish and segment the two strains based on the presence or absence of Htb2-mCherry signal intensities.

**Video 6.** **Comparative analysis of Esp1-eGFP and Htb2-mCherry signals in confocal and Airyscan2 imaging modes.** Fluorescent signals were compared between the negative control strain, *MET3-CDC20* (Y1056), and the experimental strain *HTB2-mCherry ESP1-eGFP MET3-CDC20* (Y2095), which were arrested in metaphase by Cdc20 depletion. The cells were mixed and placed in concanavalin-coated chambers, and the same cells were imaged using both confocal and Airyscan2 mode. The videos are displayed at 2 frames/sec. Video related to Fig. S5.

### Flow cytometry

DNA content in cells was assessed by flow cytometry using cells fixed with 70% ethanol and stained with propidium iodide. Flow cytometers used in this study included the Gallios (Beckman Coulter) and MACSQuant Analyzer (Miltenyi Biotec). Data analysis was performed using FlowJo (RRID:SCR_008520) (BD Biosciences).

### Whole-genome sequencing and analysis

Genomic DNA was prepared with the Nucleospin Tissue kit (Macherey-Nagel). Genomic reads were aligned to the *Saccharomyces* Genome Database reference assembly using BWA (RRID:SCR_010910) (Li, 2013, *Preprint*), then realigned with GATK (RRID:SCR_001876) (McKenna et al., 2010) to detect SNPs. In brief, SNPs were called at sites with different genotypes in a given strain than in the WT strain. Only SNPs with high coverage (≥10×) and low ambiguity (≥90% of aligned reads call the same nucleotide at that position) at both the mapped reads of the given mutant strain and the reference were accepted. This filtering procedure was able to discard likely false-positive SNPs.

### Structure analysis

Multiple sequence alignments were performed using Clustal Omega implemented in UniProt with the amino acid sequences of SAK1 (UniProt ID: P38990) and LKB1 (UniProt ID: Q15831). Structural model predictions were generated using the AlphaFold server (RRID:SCR:025885) (https://alphafoldserver.com) (Abramson et al., 2024). The models and protein interactions were analyzed using ChimeraX (RRID:SCR:015872) and PyMOL (RRID:SCR_000305) (Schrödinger).

### Statistical analysis

All experiments were performed at least three times. One representative experiment is shown for time-course experiments. Statistical analyses were conducted using GraphPad Prism (RRID: SCR_002798), using an independent two-tailed *t* test or one-way ANOVA with Tukey’s for multiple comparisons (Fig. 5 B, Fig.6 B, and Fig.7 B) to determine P values for comparisons between different assays. Data distribution was assumed to be normal, but this was not formally tested. Each cell/sample was randomly selected from a cell population. Statistical significance was set at P < 0.05. Significance levels were defined as follows: P < 0.0001 ****, P < 0.001 ***, P < 0.01 **; P < 0.05 *.

### Online supplemental material

Fig. S1 shows the suppressor genetic screen in the *esp1-2* mutant, and Table S1 lists the genetic variants identified in five suppressor mutants. Fig. S2 shows that *SAK1-E150K* did not affect Snf1 phosphorylation. Fig. S3 shows that ectopic expression of *SAK1* rescued *esp1-2* lethality. Fig. S4 shows that Sak1 catalytic activity is necessary for separase phosphorylation. Videos 1, 2, 3, 4, and 5 show separase localization in the indicated strains. Table S2 provides the quantification of cells shown in Fig. 6, B and D and Fig. 7, B and C. Table S3 lists the yeast strains used in this study. Fig. S5 compares the confocal and Airyscan2 imaging modes of Esp1-EGFP; Video 6 shows the corresponding images, and Table S4 provides quantification of these experiments. Source data include uncropped gels and plates corresponding to the Figs. 1, 2, 3, 4, 5, 7, 8, 9, 10, S2, S3, and S4.

## Supplementary Material

Review History

Table S1 lists the genetic variants identified in five suppressor mutants.

Table S2 provides the quantification of cells shown in Fig. 6, B and D and Fig. 7, B and C.

Table S3 shows the list of strains used in this study.

Table S4 provides quantification of texperiments.

SourceData F1 is the source file for Fig. 1.

SourceData F2 is the source file for Fig. 2.

SourceData F3 is the source file for Fig. 3.

SourceData F4 is the source file for Fig. 4.

SourceData F5 is the source file for Fig. 5.

SourceData F7 is the source file for Fig. 7.

SourceData F8 is the source file for Fig. 8.

SourceData F9 is the source file for Fig. 9.

SourceData F10 is the source file for Fig. 10.

SourceData FS2 is the source file for Fig. S2.

SourceData FS3 is the source file for Fig. S3.

SourceData FS4 is the source file for Fig. S4.

## Data Availability

All data are available in the article itself and its online supplemental material.
