# Supplementary material for: The AMPK kinase Sak1 activates separase and promotes its nuclear localization during mitosis
Source: J Cell Biol. 2026 Oct 20;225(11):e202505056. doi: 10.1083/jcb.202505056 (PMC13682446; doi:10.1083/jcb.202505056)
Supplement: Supplementary file 1 [file jcb_202505056_review_history.pdf]

JCB

The AMPK kinase Sak1 activates separase and promotes its nuclear localization during mitosis

David Vaquero, Jose-Antonio Rodriguez-Rodriguez, Malina Carmina Ilinca, Esther Casas-Fernandez, Amparo Picard, Francisco Del Caño-Ochoa, Isabel Vernia, Toni Gabaldon, Mari Angeles Juanes, and Ethel Queralt

Corresponding Author(s): Ethel Queralt, Consejo Superior de Investigaciones Científicas

Review Timeline:

Submission Date:	2025-05-12
Editorial Decision:	2025-06-02
Revision Received:	2025-09-05
Editorial Decision:	2025-12-02
Revision Received:	2026-04-01
Editorial Decision:	2026-05-07
Revision Received:	2026-06-25
Accepted:	2026-08-19

Monitoring Editor: Hironori Funabiki

Scientific Editor: Dan Simon

Transaction Report:

(Note: With the exception of the correction of typographical or spelling errors that could be a source of ambiguity, letters and reports are not edited. The original formatting of letters and referee reports may not be reflected in this compilation.)

DOI: https://doi.org/10.1083/jcb.202505056

June 2, 2025

Re: JCB manuscript #202505056

Ethel Queralt
Consejo Superior de Investigaciones Científicas

Dear Dr. Queralt,

Thank you for submitting your Article manuscript entitled "The yeast AMPK kinase Sak1 activates separase and promotes its nuclear localization during mitosis" to Journal of Cell Biology. As part of our normal reviewing procedure, your paper has been evaluated by at least two editors and an editorial statement is provided below. You will see that, in the consensus opinion of our editors, the manuscript is not a good fit for Journal of Cell Biology. We have thus decided not to subject your manuscript to a lengthy external review process. Because Journal of Cell Biology addresses a wide and diverse audience of cell biologists, we must give priority to manuscripts that provide a substantial advance of broad appeal to the cell biology community, even though many others also present interesting and important advances for researchers in a particular field.

I am sorry that our answer on this occasion is not more positive, and I hope that this outcome will not dissuade you from submitting other manuscripts to us in the future.

Thank you for your interest in Journal of Cell Biology.

With kind regards,

Jodi Nunnari
Editor-in-Chief
Journal of Cell Biology

Editorial Statement:

In this study the authors report that Sak1 enhances separase activity through phosphorylation and also regulates the nuclear localization of separase to promote mitotic progression independently of Snf1. The authors propose that Sak1 regulates separase through the FEAR pathway and independently of Snf1. These intriguing observations will likely be appreciated by other experts in the field. However, while the authors provide data that Sak1 may interact with and phosphorylate separase, no evidence is presented that the interaction is direct and the putative binding interface predicted by AlphaFold is not validated experimentally. Furthermore, the key experiment shown in Figure 6a is impossible to evaluate due to the poor descriptions in the figure legends and methods. The quantitation in Figure 6b is also concerning since the apparently normalized data is based on the ratio of weak signals. Again, figure legends and methods lack detail of the analysis and justification. In addition, Figure 7a also lacks a negative control to show the specificity of the co-immunoprecipitation. Due to these concerns, which relate to the major conclusions of the manuscript, this manuscript is unlikely to fare well in review at JCB.

December 2, 2025

Re: JCB manuscript #202505056R-A

Ethel Queralt
Consejo Superior de Investigaciones Científicas

Dear Dr. Queralt,

Thank you for submitting your manuscript entitled "The yeast AMPK kinase Sak1 activates separase and promotes its nuclear localization during mitosis" to the Journal of Cell Biology. The manuscript was assessed by two expert reviewers, whose comments are pasted below. We are happy to learn that both reviewers are enthusiastic to the major findings and are supportive to publication of the study, but they made several constructive criticisms that should be addressed before publication. We invite you to submit a revision if you can address the reviewers' key concerns, as outlined here.

First, Reviewer #1 requests a few additional experiments to provide stronger evidence that Sak1 directly phosphorylates Esp1 (pts #2,3) and we agree that addressing this issue is important as this is related to one of the major conclusions of the manuscript. We agree that repeating the in vitro kinase assay (Figure 5a) using a catalytic-dead mutant is a good idea. We also noticed that experimental conditions of the presented kinase assay are not fully described in the method and figure legends. Please disclose the concentration and amount of the substrate and the kinase. We assume that Esp1-FLAG and Sak1-V5 shown in Figure 5a were detected by western blotting, but we are a bit concerned if the kinase assay was conducted with limited amount of the substrate. This information is also important since pull-down of Sak1-V5 is supposed to enrich endogenous Esp1 on the beads, but you did not detect phosphorylation of the bound endogenous Esp1. Please present an entire image of the gel showing both whole protein staining and autoradiography. Related to this, JCB policy requires labeling of the molecular weight markers' positions to each gel slices presented in the manuscript. This will be very helpful for data reproducibility attempts in the future.

Second, both reviewers (particularly Reviewer #1) raise questions about the credibility of AF-based structural prediction of Sak1-Esp1 interaction. We agree that without proper annotations and analyses, it is difficult to validate this claim and to assess if the proposed conservation in human proteins is compelling. To support the major conclusion of the manuscript, the AF model can be further tested by mutation analysis. More detailed evolutionary analysis of interaction interfaces would be helpful.

Both reviewers have various other suggestions for text and figure changes to improve data presentation and provide clarifications and more detailed explanations. All of these should be addressed as well.

While you are revising your manuscript, please also attend to the following editorial points to help expedite the publication of your manuscript. Please direct any editorial questions to the journal office.

GENERAL GUIDELINES:

Text limits: Character count for an Article is < 40,000, not including spaces. Count includes title page, abstract, introduction, results, discussion, and acknowledgments. Count does not include materials and methods, figure legends, references, tables, or supplemental legends.

Figures: Articles may have up to 10 main text figures. Figures must be prepared according to the policies outlined in our Instructions to Authors, under Data Presentation, https://jcb.rupress.org/site/misc/ifora.xhtml. All figures in accepted manuscripts will be screened prior to publication.

IMPORTANT: It is JCB policy that if requested, original data images must be made available. Failure to provide original images upon request will result in unavoidable delays in publication. Please ensure that you have access to all original microscopy and blot data images before submitting your revision.

Supplemental information: There are strict limits on the allowable amount of supplemental data. Articles may have up to 5 supplemental figures. Up to 10 supplemental videos or flash animations are allowed. A summary of all supplemental material should appear at the end of the Materials and methods section.

Please note that JCB now requires authors to submit Source Data used to generate figures containing gels and Western blots with all revised manuscripts. This Source Data consists of fully uncropped and unprocessed images for each gel/blot displayed in the main and supplemental figures. For assays performed using capillary electrophoresis and/or immunoassay-based detection, authors should instead provide the electropherogram graph(s) for each experiment, plotting fluorescence/chemiluminescence intensity vs. molecular weight/size. Please be sure to provide one Source Data file for each figure gels, blots, and/or capillary electrophoresis assays along with your revised manuscript files. File names for Source Data

figures should be alphanumeric without any spaces or special characters (i.e., SourceDataF#, where F# refers to the associated main figure number or SourceDataFS# for those associated with Supplementary figures). For traditional gels and blots, the lanes of the gels/blots should be labeled as they are in the associated figure, the place where cropping was applied should be marked (with a box), and molecular weight/size standards should be labeled wherever possible. For capillary electrophoresis assays, each trace in the graph should be color-coded and labeled to indicate which protein, gene, or sample is being measured (please try to avoid red/green combinations to accommodate our color-blind readers).

Source Data files will be made available to reviewers during evaluation of revised manuscripts and, if your paper is eventually published in JCB, the files will be directly linked to specific figures in the published article.

Source Data Figures should be provided as individual PDF files (one file per figure). Authors should endeavor to retain a minimum resolution of 300 dpi or pixels per inch. Please review our instructions for export from Photoshop, Illustrator, and PowerPoint here: https://rupress.org/jcb/pages/submission-guidelines#revised

The typical timeframe for revisions is three to four months. If you anticipate any difficulties in meeting this aforementioned revision time limit, please contact us and we can work with you to find an appropriate time frame for resubmission. Please note that papers are generally considered through only one revision cycle, so any revised manuscript will likely be either accepted or rejected.

When submitting the revision, please include a cover letter addressing the reviewers' comments point by point. Please also highlight all changes in the text of the manuscript.

We hope that the comments below will prove constructive as your work progresses. We would be happy to discuss them further once you've had a chance to consider the points raised in this letter.

Thank you for this interesting contribution to Journal of Cell Biology. You can contact us at the journal office with any questions at cellbio@rockefeller.edu.

Sincerely,

Hironori Funabiki, PhD
Monitoring Editor
Journal of Cell Biology

Dan Simon, PhD
Scientific Editor
Journal of Cell Biology

Reviewer #1 (Comments to the Authors (Required)):

In this paper, Vaquero-Pelaz and colleagues report the identification of the kinase Sak1 as a new regulator of S. cerevisiae separase (Esp1).

The authors identified a hyperactive allele of SAK1 (E50K) in an esp1 mutant suppressor screen. They provide evidence that Sak1 binds and phosphorylates Esp1 and show that it promotes nuclear enrichment of Esp1. The influence of Sak1 on separase is independent of another known Sak1 substrate, Snf1.

Overall, the study is well conducted with well-controlled and well-presented experiments. Given the central importance of Esp1 for cell division, the identification of a new regulator is interesting. The role of Sak1 in promoting nuclear enrichment of Esp1 is well supported by the data.

Weaknesses are:
(1) The relevant phosphorylation site(s) on Esp1 have not been mapped, and the role of Sak1-mediated Esp1 phosphorylation therefore has not been formally established. This seems difficult to fix in the short term, since the authors mention that they have been unable to identify the residues phosphorylated by Sak1 using mass spectrometry.

(2) The identity of the kinase that phosphorylates Esp1 in the in vitro kinase assay (Fig. 5a/b) is not yet established beyond doubt. It remains possible that the phosphorylation is due to a kinase that co-precipitates with Sak1. This could be addressed by using a catalytic-dead Sak1 mutant in the assay.

(3) The authors convincingly observe a Sak1-Esp1 interaction (Fig. 5, 7) and suggest this is a direct interaction based on an AlphaFold 3 model. However, the model confidence scores are low. This part could be considerably strengthened by predicting

residues critical for the Sak1/separase interaction and mutating them.

(4) The evidence for evolutionary conservation is weak. The authors present an AlphaFold 3 prediction for an interaction between human separase and LKB1 (the Sak1 ortholog) along with two LKB1 binding partners. However, the confidence scores are even worse than for the Sak1/Esp1 interaction, and I don't think these data can be considered solid evidence of interaction. To strengthen this, and if antibodies are available, a relatively easy experiment would be to immunoprecipitate either Sak1 or Esp1 from human cell lysates and test for co-immunoprecipitation of the respective other protein.

Other minor comments:

(1) The immunoblots of Pds1 in Fig. 2b and 4a are striking. It looks like there is a huge mobility shift (cut off at the top) right around the time when Pds1 is supposedly degraded. This is missing in the esp1-2 mutant and rescued by SAK1-E50K. What is the identity of this upper band?

(2) Is the double-deletion of PDS1 and SAK1 synthetic lethal, i.e. are they likely to act in the same or parallel pathways?

(3) In Figure 3c, I find it hard to reconcile the images of WT and sak1Δ, which look highly similar, with the quantification shown on the right side. Would it be possible to provide example images from the 9-minute time point where the difference is the most pronounced?

(4) The phosphorylation assay in Figure 5B could be improved by a PPase treatment, which would allow for a more solid quantification of the total amount of Esp1 and would confirm that shifts are due to phosphorylation.

Other stylistic comments:
- Fig. 2a: While there is a clear rescue in the esp1-2 SAK1-E50K double mutant, the cells don't look entirely normal. I would therefore suggest removing "normal" in the figure legend.
- When quantifying nuclear Esp1-GFP, the authors seem to have generated tracks over time (Methods section, lines 894-896). Could the full tracks be shown instead of binning into categories (Fig. 6c/d)?
- In Figure 7, the schematic (and particularly the font size on the schematic) is tiny relative to the rest of the figure.
- In Figure S8a, the size of cells relative to the font sizes is tiny. Maybe provide zoomed-in images instead or in addition to the current overview images.
- Please label the individual protein chains in the PAE plots (Fig. 7e,f).
- Line 207: "genetic interaction" instead of just "interaction" would be more precise.
- Line 403/404: "separase still localizes to the nucleus in the absence of securin" is a little misleading - separase's localization to the nucleus is greatly reduced. This is clear from the pictures, but not clearly stated in the text.
- Line 548: sak1-E50K does not rescue the viability of esp1Δ. Maybe I overlooked it, but I don't think this is shown in the paper. Please either refer to the relevant figure, add "data not shown," or add the data.
- Line 837 "containing" 1 mg/mL concanavalin A: Maybe "coated with" would be more appropriate.
- Line 846 - 874: This reads a bit like an advertising text for the Airyscan. I think it's sufficient to mention the specific settings used in this study.

Reviewer #2 (Comments to the Authors (Required)):

In this interesting study, Vaquero-Pelaz et al. identify an intriguing new regulator of the key cell cycle protease separase. Separase orchestrates mitotic progression during eukaryotic cell divisions, including triggering anaphase by cohesin cleavage, as well as using a non-proteolytic role to promote cell cycle progression. Despite its importance, the regulation of separase remains incompletely understood. In this study, the authors identify the Sak1 kinase as a previously unknown interactor and regulator of separase function. The experiments are overall well executed, but see below, and the results support the authors' conclusions. The study culminates in an AlphaFold 3 model of the Sak1-separase complex. I can recommend this study for publication in the Journal of Cell Biology, after the authors have given consideration to the following points.

1. The authors provide a comprehensive introduction to the field. While an introduction need not review all of the available literature, there are two noteworthy omissions
a) Line 88: "...spindle elongation and stabilization (Nagao and Yanagida, 2006)" should probably include a reference to Higuchi et al. 2005, who made these observations the year before.
b) It might be worth referring the reader to the new structural study on the mechanism of separase substrate recognition by Yu et al. 2025.

2. The description of the screen that identified Sak1 remains unclear. Line 165: "The esp1-2 mutant was inoculated at 30C (a semi-restrictive temperature), and the resulting colonies were presumed to contain suppressor mutants". How were cells

"inoculated", a term that refers to starting a liquid culture? Were cells streaked out on a plate? Further, at semi-restrictive temperature a ts strain grows slowly, so how were suppressors identified? Were colonies observed that markedly outgrew cells in the surrounding? Please explain.

3. Line 200: "Sak1 is one of the three kinases responsible for phosphorylating and activating Snf1" it would be nice to introduce Snf1 in a few words.

4. Figure S2a, b, please indicate what experimental conditions are compared between these two panels, as the labels look similar but report different outcomes. Furthermore, what does the time progression indicate?

5. Figure 2c, the authors conclude that separase levels remain unaffected by the esp1-2 mutation and by Sak1. In contrast, visual inspection of the blot suggests that separase levels are reduced by the esp1-2 mutation, and that levels are restored by the Sak1-E50K allele. Have the authors normalised separase levels to that of their loading control? Can the gel be loaded more evenly?

6. Lines 294 and 373: "To test this, ...". "This" is a demonstrative adjective that should not replace a subject, the authors probably mean "To test this possibility, ..." or similar.

7. Line 375: "Immunopurified Sak1 and SAK1-E50K", please provide a brief description in the main text of how purification was achieved.

8. Figure 6d would be most meaningful if a comparison of pds1del and pds1del SAK1-E50K cells was presented.

9. Figure 7e,f concludes the authors' genetic work by revealing an AlphaFold3-predicted, conserved Sak1-separase complex. These panels represent important results that should be covered in the Results section, not the Discussion.
a) Can the securin colour be made more easily distinguishable from the separase colour?
b) The prediction confidence plots are missing protein annotations.
c) Can the position of the E50K mutation be indicated, and does its location suggest a possible mechanism for the gain of function effect? This question would be important to discuss.

10. The discussion is unduly lengthy, containing unnecessary repetition of the results.

Reviewer #1 (Comments to the Authors (Required)):

In this paper, Vaquero-Pelaz and colleagues report the identification of the kinase Sak1 as a new regulator of S. cerevisiae separase (Esp1).

The authors identified a hyperactive allele of SAK1 (E50K) in an esp1 mutant suppressor screen. They provide evidence that Sak1 binds and phosphorylates Esp1 and show that it promotes nuclear enrichment of Esp1. The influence of Sak1 on separase is independent of another known Sak1 substrate, Snf1.

Overall, the study is well conducted with well-controlled and well-presented experiments. Given the central importance of Esp1 for cell division, the identification of a new regulator is interesting. The role of Sak1 in promoting nuclear enrichment of Esp1 is well supported by the data.

We thank Reviewer 1 for their positive and constructive assessment of our work and helpful comments on the manuscript.

Weaknesses are:
(1) The relevant phosphorylation site(s) on Esp1 have not been mapped, and the role of Sak1-mediated Esp1 phosphorylation therefore has not been formally established. This seems difficult to fix in the short term, since the authors mention that they have been unable to identify the residues phosphorylated by Sak1 using mass spectrometry.

We have recently intensified our efforts to identify separase phosphorylation sites using mass spectrometry. This analysis revealed two putative phosphorylation sites, Ser1202 and Ser1204. However, mutation of these residues to alanine did not alter separase nuclear localization, suggesting that these sites are not critical for its subcellular translocation.

Despite employing double digestion with trypsin and Glu-C to reduce peptide size, the overall sequence coverage for separase remained below 30%, limiting our ability to detect additional phosphorylation sites. Improving peptide preparation strategies and enhancing ionization efficiency in the mass spectrometer will be necessary to increase the likelihood of identifying further regulatory phosphorylation. At this stage, we are unable to advance this point further.

(2) The identity of the kinase that phosphorylates Esp1 in the in vitro kinase assay (Fig. 5a/b) is not yet established beyond doubt. It remains possible that the phosphorylation is due to a kinase that co-precipitates with Sak1. This could be addressed by using a catalytic-dead Sak1 mutant in the assay.

We thank the reviewer for highlighting this issue. We have performed a new kinase assay using a catalytically inactive mutant of Sak1, now included in Fig. S4A.

As described in the revised manuscript, Sak1 was immunopurified under high-detergent conditions (1% TritonX-100) to prevent co-purification of endogenous Esp1 or any other kinase.

To further improve clarity and reproducibility, we have added additional methodological details to the Materials and Methods section.

(3) The authors convincingly observe a Sak1-Esp1 interaction (Fig. 5, 7) and suggest this is a direct interaction based on an AlphaFold 3 model. However, the model confidence scores are low. This part could be considerably strengthened by predicting residues critical for the Sak1/separase interaction and mutating them.

We appreciate the reviewer's concern about the AF-prediction and the suggestion to mutate key residues involved in the Sak1-Esp1 interaction, which we agree would strengthen our conclusions. We have refined the structural prediction, and the model confidence scores improved notably after incorporating cofactors $MgCl_2$, and ATP.

In addition, we have analyzed the predicted interaction interface and identified two residues within separase and Sak1 that appeared critical for the interaction. Mutating these residues resulted in a reduction in their association by co-immunoprecipitation, supporting the existence of a direct interaction. All these new results are now included in figure 9.

(4) The evidence for evolutionary conservation is weak. The authors present an AlphaFold 3 prediction for an interaction between human separase and LKB1 (the Sak1 ortholog) along with two LKB1 binding partners. However, the confidence scores are even worse than for the Sak1/Esp1 interaction, and I don't think these data can be considered solid evidence of interaction. To strengthen this, and if antibodies are available, a relatively easy experiment would be to immunoprecipitate either Sak1 or Esp1 from human cell lysates and test for co-immunoprecipitation of the respective other protein.

We greatly appreciate this reviewer's suggestion, as we agree that this analysis substantially strengthens our conclusions. We performed co-immunoprecipitation experiments in human HEK293T cells, and we detected a clear physical association of human separase and Lkb1. These new results are now included in Fig. 10.

Other minor comments:

(1) The immunoblots of Pds1 in Fig. 2b and 4a are striking. It looks like there is a huge mobility shift (cut off at the top) right around the time when Pds1 is supposedly degraded. This is missing in the esp1-2 mutant and rescued by SAK1-E50K. What is the identity of this upper band?

This upper band correspond to Cdc20-HA, which is detected by the anti-HA antibody used in these experiments. Because we synchronize cells in metaphase by Cdc20 depletion, the Cdc20-HA band is absent during the arrest and reappears upon release, when new Cdc20 protein is synthesized. In the *esp1-2* mutant, Cdc20 levels are markedly reduced, and the band is only visible in highly exposed blots, explaining why it appears faint or absent in those samples.

(2) Is the double-deletion of PDS1 and SAK1 synthetic lethal, i.e. are they likely to act in the same or parallel pathways?

We attempted to address this point using several complementary approaches. Despite numerous attempts, we were unable to generate a *pds1*Δ *sak1*Δ double deletion strain by direct transformation. Since securin is essential during meiosis, standard genetic crossing with a *pds1*Δ mutant was also not feasible.

We next generated conditional double mutants: *GAL1-SAK1 pds1*Δ and *pds1*-aid *sak1*Δ. Although these strains grew on glucose or auxin plates, western blot analysis revealed presence of Sak1 after shut-off of the galactose promoter and incomplete degradation of *pds1-aid* upon auxin addition. These partial depletions prevented us from reliably assessing the phenotype of the double mutant.

Taken together, although we think that the *pds1*Δ *sak1*Δ double mutant may be lethal, we are unable to definitively demonstrate lethality at this time.

(3) In Figure 3c, I find it hard to reconcile the images of WT and sak1Δ, which look highly similar, with the quantification shown on the right side. Would it be possible to provide example images from the 9-minute time point where the difference is the most pronounced?

We thank the reviewer for pointing this out. We have replaced the previously shown WT cells with an image that more accurately reflects the quantification results.

(4) The phosphorylation assay in Figure 5B could be improved by a PPase treatment, which would allow for a more solid quantification of the total amount of Esp1 and would confirm that shifts are due to phosphorylation.

The experiment shown in Fig. 5B was performed using denaturing TCA protein extracts, and under these conditions, PPase treatment cannot be applied. We attempted to detect Esp1 phosphorylation using native protein extracts, but the phosphorylation-dependent band shift was not detectable, which prevented us from performing the phosphatase assay as suggested. Nevertheless, the fact that the separase band resolved in a phostag gels strongly suggests that the protein is phosphorylated.

Other stylistic comments:
- Fig. 2a: While there is a clear rescue in the esp1-2 SAK1-E50K double mutant, the cells don't look entirely normal. I would therefore suggest removing "normal" in the figure legend.

We have incorporated the suggested text change and removed “normal” in the figure legend.

- When quantifying nuclear Esp1-GFP, the authors seem to have generated tracks over time (Methods section, lines 894-896). Could the full tracks be shown instead of binning into categories (Fig. 6c/d)?

We thank the reviewer for this comment. The Cell tracking functionality in the Arivis Pro AI-based software is used to follow each individual cell over time for automatic quantification. The “population plot” shown in Fig. 6C already displays the quantified nuclear Esp1-GFP signal across all time points for multiple tracked cells, thereby reflecting the full trajectories obtained from the tracking workflow.

In contrast, Fig. 6D focuses specifically on the pre-anaphase cells, where the differences between strains are observed. Taken together, these two representations already provide the full temporal information requested by the reviewer.

- In Figure 7, the schematic (and particularly the font size on the schematic) is tiny relative to the rest of the figure.

We have increased the size of the schematic representation as suggested.

- In Figure S8a, the size of cells relative to the font sizes is tiny. Maybe provide zoomed-in images instead or in addition to the current overview images.

We have provided zoomed-in cells in the images as suggested.

- Please label the individual protein chains in the PAE plots (Fig. 7e,f).

We have labelled the individual protein chains as suggested to improve clarity.

- Line 548: sak1-E50K does not rescue the viability of esp1Δ. Maybe I overlooked it, but I don't think this is shown in the paper. Please either refer to the relevant figure, add "data not shown," or add the data.

JCB policy does not allow for “data not shown”. Therefore, we have removed it from the text.

- Line 207: "genetic interaction" instead of just "interaction" would be more precise.

- Line 403/404: "separase still localizes to the nucleus in the absence of securin" is a little misleading - separase's localization to the nucleus is greatly reduced. This is clear from the pictures, but not clearly stated in the text.
- Line 837 "containing" 1 mg/mL concanavalin A: Maybe "coated with" would be more appropriate.
- Line 846 - 874: This reads a bit like an advertising text for the Airyscan. I think it's sufficient to mention the specific settings used in this study.

All these suggested text changes have been incorporated.

Reviewer #2 (Comments to the Authors (Required)):

In this interesting study, Vaquero-Pelaz et al. identify an intriguing new regulator of the key cell cycle protease separase. Separase orchestrates mitotic progression during eukaryotic cell divisions, including triggering anaphase by cohesin cleavage, as well as using a non-proteolytic role to promote cell cycle progression. Despite its importance, the regulation of separase remains incompletely understood. In this study, the authors identify the Sak1 kinase as a previously unknown interactor and regulator of separase function. The experiments are overall well executed, but see below, and the results support the authors' conclusions. The study culminates in an AlphaFold 3 model of the Sak1-separase complex. I can recommend this study for publication in the Journal of Cell Biology, after the authors have given consideration to the following points.

We are grateful to the reviewer for her/his positive assessment of the work and helpful comments on the manuscript.

1. The authors provide a comprehensive introduction to the field. While an introduction need not review all of the available literature, there are two noteworthy omissions
a) Line 88: "...spindle elongation and stabilization (Nagao and Yanagida, 2006)" should probably include a reference to Higuchi et al. 2005, who made these observations the year before.
b) It might be worth referring the reader to the new structural study on the mechanism of separase substrate recognition by Yu et al. 2025.

We apologize about it. The Higuchi reference was included in earlier versions of the manuscript and removed by mistake. It has now been restored in the revised manuscript. In addition, the structural study of Yu et al., which was a preprint at the time of our original submission, has since been published and has also been incorporated into the updated version of the manuscript.

2. The description of the screen that identified Sak1 remains unclear. Line 165: "The esp1-2 mutant was inoculated at 30C (a semi-restrictive temperature), and the resulting colonies were presumed to contain suppressor mutants". How were cells "inoculated", a term that refers to starting a liquid culture? Were cells streaked out on a plate? Further, at semi-restrictive temperature a ts strain grows slowly, so how were suppressors identified? Were colonies observed that markedly outgrew cells in the surrounding? Please explain.

Thank you for pointing out the need for clarification. To identify suppressors of *esp1-2*, a liquid culture of the *esp1-2* strain was prepared, and approximately 1 million cells were spread onto YPD plates and incubated at 30ºC, a restrictive temperature for this allele. Under these conditions, the *esp1-2* mutant is unable to form colonies and dies, allowing only spontaneous suppressor mutants to grow. As a result, distinct, well-defined colonies appeared on the plate, each corresponding to an independent suppressor. However, at 35ºC and 37ºC, no colonies were grown; for this reason, we stated a semi-restrictive temperature.

We have changed the text accordingly to clarify this in Material and Methods.

3. Line 200: "Sak1 is one of the three kinases responsible for phosphorylating and activating Snf1" it would be nice to introduce Snf1 in a few words.

We have included the sentence "The Snf1/AMPK pathway regulates cellular adaptation to alternative carbon sources and serves as a key sensor of cellular stress (Nath et al., 2003; Hong and Carlson, 2007; McCartney et al., 2005; Elbing et al., 2006)" to introduce Snf1 as suggested.

4. Figure S2a, b, please indicate what experimental conditions are compared between these two panels, as the labels look similar but report different outcomes. Furthermore, what does the time progression indicate?

We thank the reviewer for this comment. For clarity, we have now indicated in the figure that the experiment was performed in glucose (Fig. S2A) or galactose (Fig. S2B). In addition, we added the label "metaphase release" to the time points to make explicit that these samples correspond to cells synchronyzed in mitosis by Cdc20 depletion (as described in the figure legends).

5. Figure 2c, the authors conclude that separase levels remain unaffected by the esp1-2 mutation and by Sak1. In contrast, visual inspection of the blot suggests that separase levels are reduced by the esp1-2 mutation, and that levels are restored by the Sak1-E50K allele. Have the authors normalised separase levels to that of their loading control? Can the gel be loaded more evenly?

We thank the reviewer for this observation. We agree that the *esp1-2* mutant appears to show reduced separase levels by visual inspection of the blot. However, this effect is frequently observed for certain unstable or cell-cycle-regulated proteins in *esp1-2* cultures, where a fraction of cells is non-viable at the semi-restrictive temperature. This phenomenon affects some proteins (e.g. Pds1 or Sic1) but not others (e.g. Clb2 or Scc1), resulting in an apparent reduction in band intensity that does not reflect true differences in protein abundance.

To address this quantitatively, we normalized separase levels to the loading control across all biological replicates. After quantification and normalization, no significant differences in Esp1 levels were detected between the conditions.

Finally, Esp1 is highly unstable in protein extracts, and despite multiple attempts, the blot shown represents the most evenly loaded and reproducible result we were able to obtain.

6. Lines 294 and 373: "To test this, ...". "This" is a demonstrative adjective that should not replace a subject, the authors probably mean "To test this possibility, ..." or similar.

We thank the reviewer for this comment and have implemented the suggested correction. The phrasing "To test this" has been replaced with "to test this possibility" in the revised manuscript.

7. Line 375: "Immunopurified Sak1 and SAK1-E50K", please provide a brief description in the main text of how purification was achieved.

The sentence "Sak1 and Sak1-E50K were immunopurified by pulling down V5-tagged proteins using anti-V5 antibody and Protein A-Dynabeads," has been introduced in the manuscript to clarify this point.

8. Figure 6d would be most meaningful if a comparison of pds1del and pds1del SAK1-E50K cells was presented.

We thank the reviewer for highlighting this point. We have rearranged the figures so that the images from *pds1Δ*, which were previously included in the supplementary figures, now appear alongside the *pds1Δ SAK1-E50K* strain in new Fig. 7, allowing for the direct comparison.

9. Figure 7e,f concludes the authors' genetic work by revealing an AlphaFold3-predicted, conserved Sak1-separase complex. These panels represent important results that should be covered in the Results section, not the Discussion.
a) Can the securin colour be made more easily distinguishable from the separase colour?
b) The prediction confidence plots are missing protein annotations.
c) Can the position of the E50K mutation be indicated, and does its location suggest a possible mechanism for the gain of function effect? This question would be important to discuss.

We are grateful to the reviewer for these suggestions. In accordance with the comments, we have moved the AlphaFold3-based analysis from the Discussion to the Results section. We have also modified the figures as requested: the securin colour has been adjusted to make it clearly distinguishable from separase, protein annotations have been added to the prediction confidence plots, and the position of the E50K mutation is now indicated.

In addition, we have analyzed the predicted interaction interface and identified two residues within separase and Sak1 that appeared critical for the interaction. Mutating these residues resulted in a measurable reduction in their association by co-immunoprecipitation, supporting the existence of a direct interaction. All these new results are now included in figure 9.

Moreover, we have generated an improved AF prediction with higher confidence scores by incorporating the appropriate cofactors (MgCl2 and ATP). We also include new experimental data demonstrating a physical association between human separase and Lkb1, further supporting the evolutionary conservation of the Sak1-Esp1 interaction (Fig. 10).

Although the E50K substitution is not located within the predicted kinase domain nor directly at the Esp1-Sak1 interaction surface, gain-of-function mutations frequently arise from allosteric or regulatory effects rather than from changes in catalytic or binding residues. A charge-changing substitution, such as Glu to Lys can alter electrostatic properties, local folding, protein dynamics, or intra-molecular contacts, thereby shifting the conformational equilibrium of Sak1 toward a more active or more interaction-competent state. Thus, the gain-of-function phenotype of Sak1-E50K likely reflects an indirect structural or regulatory effect, rather than a direct modification of the active site or the Esp1-binding interface. This idea has been introduced in the Discussion.

10. The discussion is unduly lengthy, containing unnecessary repetition of the results.

The Discussion section has been reduced as suggested by the reviewer.

May 7, 2026

RE: JCB Manuscript #202505056RR

Ethel Queralt
Consejo Superior de Investigaciones Científicas

Dear Dr. Queralt,

Thank you for submitting your revised manuscript entitled "The yeast AMPK kinase Sak1 activates separase and promotes its nuclear localization during mitosis." We would be happy to publish your paper in JCB pending final revisions necessary to address the remaining minor reviewer comments and to meet our formatting guidelines (see details below).

To avoid unnecessary delays in the acceptance and publication of your paper, please read the following information carefully.

A. MANUSCRIPT ORGANIZATION AND FORMATTING:

Full guidelines are available on our Instructions for Authors page, https://jcb.rupress.org/submission-guidelines#revised. **Submission of a paper that does not conform to JCB guidelines will delay the acceptance of your manuscript.**

1) Text limits: Character count for Articles is < 40,000, not including spaces. Count includes title page, abstract, introduction, results, discussion, and acknowledgments. Count does not include materials and methods, figure legends, references, tables, or supplemental legends.

2) Figure formatting: Articles may have up to 10 main text figures. Scale bars must be present on all microscopy images, including inset magnifications. Molecular weight or nucleic acid size markers must be included on all gel electrophoresis. Please increase the size of scale bars in Figure S5A and add separate scale bars for inset magnifications in this panel.

Also, please avoid pairing red and green for images and graphs to ensure legibility for color-blind readers. If red and green are paired for images, please ensure that the particular red and green hues used in micrographs are distinctive with any of the colorblind types. If not, please modify colors accordingly or provide separate images of the individual channels.

3) Statistical analysis: Error bars on graphic representations of numerical data must be clearly described in the figure legend. The number of independent data points (n) represented in a graph must be indicated in the legend. Please indicate whether 'n' refers to technical or biological replicates (i.e. number of analyzed cells, samples or animals, number of independent experiments). If independent experiments with multiple biological replicates have been performed, we recommend using distribution-reproducibility SuperPlots (please see Lord et al., JCB 2020) to better display the distribution of the entire dataset, and report statistics (such as means, error bars, and P values) that address the reproducibility of the findings.

Statistical methods should be explained in full in the materials and methods. For figures presenting pooled data the statistical measure should be defined in the figure legends. Please also be sure to indicate the statistical tests used in each of your experiments (both in the figure legend itself and in a separate methods section) as well as the parameters of the test (for example, if you ran a t-test, please indicate if it was one- or two-sided, etc.). Also, if you used parametric tests, please indicate if the data distribution was tested for normality (and if so, how). If not, you must state something to the effect that "Data distribution was assumed to be normal but this was not formally tested."

4) Title: JCB discourages the usage of species or organism names in titles in order to make papers more accessible to a general readership. Please remove the word 'yeast' from your title.

5) Materials and methods: Should be comprehensive and not simply reference a previous publication for details on how an experiment was performed. Please provide full descriptions (at least in brief) in the text for readers who may not have access to referenced manuscripts. The text should not refer to methods "...as previously described." Please also indicate the acquisition and quantification methods for immunoblotting/western blots.

6) For all cell lines, vectors, strains, constructs/cDNAs, etc. - all genetic material: please include database / vendor ID (e.g. Addgene, ATCC, etc.) or if unavailable, please briefly describe their basic genetic features, even if described in other published work or gifted to you by other investigators (and provide references where appropriate). Please be sure to provide the sequences for all of your oligos: primers, si/shRNA, RNAi, gRNAs, etc. in the materials and methods. You must also indicate in the methods the source, species, and catalog numbers/vendor identifiers (where appropriate) for all of your antibodies, including secondary. If antibodies are not commercial, please add a reference citation if possible.

7) Microscope image acquisition: The following information must be provided about the acquisition and processing of images:
a. Make and model of microscope
b. Type, magnification, and numerical aperture of the objective lenses
c. Temperature
d. Imaging medium
e. Fluorochromes
f. Camera make and model
g. Acquisition software
h. Any software used for image processing subsequent to data acquisition. Please include details and types of operations involved (e.g., type of deconvolution, 3D reconstitutions, surface or volume rendering, gamma adjustments, etc.).

8) References: There is no limit to the number of references cited in a manuscript. References should be cited parenthetically in the text by author and year of publication. Abbreviate the names of journals according to PubMed.

9) Supplemental materials: Articles may have up to 5 supplemental figures and 10 videos. Please also note that tables, like figures, should be provided as individual, editable files. A summary of all supplemental material should appear at the end of the Materials and methods section. Please include one brief sentence per item.

10) Video legends: Should describe what is being shown, the cell type or tissue being viewed (including relevant cell treatments, concentration and duration, or transfection), the imaging method (e.g., time-lapse epifluorescence microscopy), what each color represents, how often frames were collected, the frames/second display rate, and the number of any figure that has related video stills or images.

11) eTOC summary: A ~40-50 word summary that describes the context and significance of the findings for a general readership should be included on the title page. The statement should be written in the present tense and refer to the work in the third person. It should begin with "First author name(s) et al..." to match our preferred style.

12) Conflict of interest statement: JCB requires inclusion of a statement in the acknowledgements regarding competing financial interests. If no competing financial interests exist, please include the following statement: "The authors declare no competing financial interests." If competing interests are declared, please follow your statement of these competing interests with the following statement: "The authors declare no further competing financial interests."

13) A separate author contribution section is required following the Acknowledgments in all research manuscripts. All authors should be mentioned and designated by their first and middle initials and full surnames. We encourage use of the CRediT nomenclature (https://casrai.org/credit/).

14) ORCID IDs: ORCID IDs are unique identifiers allowing researchers to create a record of their various scholarly contributions in a single place. Please note that ORCID IDs are required for all authors. At resubmission of your final files, please be sure to provide your ORCID ID and those of all co-authors.

15) JCB requires authors to submit Source Data used to generate figures containing gels and Western blots with all revised manuscripts. This Source Data consists of fully uncropped and unprocessed images for each gel/blot displayed in the main and supplemental figures. For assays performed using capillary electrophoresis and/or immunoassay-based detection, authors should instead provide the electropherogram graph(s) for each experiment, plotting fluorescence/chemiluminescence intensity vs. molecular weight/size. Since your paper includes cropped gel and/or blot images, please be sure to provide one Source Data file for each figure gels, blots, and/or capillary electrophoresis assays along with your revised manuscript files. File names for Source Data figures should be alphanumeric without any spaces or special characters (i.e., SourceDataF#, where F# refers to the associated main figure number or SourceDataFS# for those associated with Supplementary figures). For traditional gels and blots, the lanes of the gels/blots should be labeled as they are in the associated figure, the place where cropping was applied should be marked (with a box), and molecular weight/size standards should be labeled wherever possible. For capillary electrophoresis assays, each trace in the graph should be color-coded and labeled to indicate which protein, gene, or sample is being measured (please try to avoid red/green combinations to accommodate our color-blind readers).

Source Data files will be directly linked to specific figures in the published article. Source Data Figures should be provided as individual PDF files (one file per figure). Authors should endeavor to retain a minimum resolution of 300 dpi or pixels per inch. Please review our instructions for export from Photoshop, Illustrator, and PowerPoint here: https://rupress.org/jcb/pages/submission-guidelines#revised.

16) Journal of Cell Biology now requires a data availability statement for all research article submissions. These statements will be published in the article directly above the Acknowledgments. The statement should address all data underlying the research presented in the manuscript. Please visit the JCB instructions for authors for guidelines and examples of statements at (https://rupress.org/jcb/pages/editorial-policies#data-availability-statement).

B. FINAL FILES:

Please upload the following materials to our online submission system. These items are required prior to acceptance. If you have any questions, contact JCB's Managing Editor, Lindsey Hollander (lhollander@rockefeller.edu).

-- An editable version of the final text (.DOC or .DOCX) is needed for copyediting (no PDFs).

-- A response to reviewers document addressing the remaining comments point by point. Please also highlight all changes in the text of the manuscript.

-- High-resolution figure and MP4 video files: See our detailed guidelines for preparing your production-ready images, https://jcb.rupress.org/fig-vid-guidelines.

-- Cover images: If you have any striking images related to this story, we would be happy to consider them for inclusion on the journal cover. Submitted images may also be chosen for highlighting on the journal table of contents or JCB homepage carousel. Images should be uploaded as TIFF or EPS files and must be at least 300 dpi resolution.

It is JCB policy that if requested, original data images must be made available to the editors. Failure to provide original images upon request will result in unavoidable delays in publication. Please ensure that you have access to all original data images prior to final submission.

The license to publish form must be signed before your manuscript can be sent to production. A link to the license to publish form will be sent to the corresponding author only. Please take a moment to check your funder requirements before choosing the appropriate license.

Additionally, JCB encourages authors to submit a short video summary of their work. These videos are intended to convey the main messages of the study to a non-specialist, scientific audience. Think of them as an extended version of your abstract, or a short poster presentation. We encourage first authors to present the results to increase their visibility. The videos will be shared on social media to promote your work. For more detailed guidelines and tips on preparing your video, please visit https://rupress.org/jcb/pages/submission-guidelines#videoSummaries.

Thank you for your attention to these final processing requirements. Please revise and format the manuscript and upload materials within 14 days. If you need an extension for whatever reason, please let us know and we can work with you to determine a suitable revision period.

Please contact the journal office with any questions at cellbio@rockefeller.edu.

Thank you for this interesting contribution, we look forward to publishing your paper in Journal of Cell Biology.

Sincerely,

Hironori Funabiki, PhD
Monitoring Editor
Journal of Cell Biology

Dan Simon, PhD
Scientific Editor
Journal of Cell Biology

Reviewer #1 (Comments to the Authors (Required)):

The authors have adequately addressed my previous comments.

The addition of the in vitro kinase assay with catalytically dead Sak1 (Fig. S4A), mutation of residues at the interface (Fig. 9C), and the co-immunoprecipitation of human separase with LKB1 (Fig. 10C) have strengthened the paper.

I have a few remaining suggestions for text changes:

(1) Figure 2D and 4A: The authors explained in the reply to the reviewers that the additional, slower migrating band visible in the Pds1 immunoblot is Cdc20-HA. To avoid confusion, I think it would be useful to indicate this with an asterisk and a mention in the figure legend.

(2) In Figure 5B, it is unclear to me how the quantification is done. (I felt it's not clear enough in the legend, and I didn't see it in the Methods section, either.) If this quantifies the ratio of slower and faster migrating band in the Phos-tag gel, I don't see how

the ratio can be higher in SAK1-E50K than in WT. SAK1-E50K has a more prominent slower migrating band, but also seems to have a faster migrating band of considerable intensity, whereas I don't see this in the WT lane. I would appreciate it if the authors provided a little more detail on the quantification in the legend.

(3) The text editing in line 418 - 423 made this hard to understand.
The "However, ..." doesn't seem to fit anymore, and the logic isn't clear.
I suggest:
"... tightly regulated by securin to ensure accurate chromosome segregation during mitosis. Nuclear localization of separase is greatly reduced, but not abolished, in the absence of securin. This suggests an additional mechanism controlling the nuclear localization of separase, and we hypothesized that Sak1-mediated phosphorylation of separase promotes its nuclear localization."

(4) Line 451 on securin/separase could additionally cite Yu et al., 2021 - but at the authors' discretion.

(5) Line 481 - 483: the phrasing is a little odd, and conservation for some of the residues doesn't seem high.
Maybe color-code residues to make it easier to see conservation and show the larger sequence context in the supplement?

(6) Typos introduced when editing the discussion:
Line 556 should be "mitosis" rather than "mitotic."
Line 568 "nucleolar..." should be "nucleolar release."
Line 576: Even though some text has been removed, it could be useful to still refer to the figure showing the independence from Snf1.

(7) Line 654: Isn't it a "restrictive" temperature if esp1-2 mutants are unable to grow at this temperature?
It could be called "restrictive," and then adding a sentence: "None of the esp1-2 strains with suppressor mutation obtained at 30 ºC grew at 35 ºC or 37 ºC." I think this would be useful as it provides additional information.

(8) Figure 10A: The legend mentions rmsd, but I don't see it in the figure. (Entirely possible that I'm overlooking it.)

Reviewer #1 (Comments to the Authors (Required)):

The authors have adequately addressed my previous comments.

We sincerely thank the reviewer for the constructive comments.

The addition of the in vitro kinase assay with catalytically dead Sak1 (Fig. S4A), mutation of residues at the interface (Fig. 9C), and the co-immunoprecipitation of human separase with LKB1 (Fig. 10C) have strengthened the paper.

I have a few remaining suggestions for text changes:

(1) Figure 2D and 4A: The authors explained in the reply to the reviewers that the additional, slower migrating band visible in the Pds1 immunoblot is Cdc20-HA. To avoid confusion, I think it would be useful to indicate this with an asterisk and a mention in the figure legend.

We greatly appreciate this reviewer's suggestion and incorporated the * in the blots and the text "(*) Marks Cdc20-HA band detected by the anti-HA antibody" in the figure legends 2D and 4A.

(2) In Figure 5B, it is unclear to me how the quantification is done. (I felt it's not clear enough in the legend, and I didn't see it in the Methods section, either.) If this quantifies the ratio of slower and faster migrating band in the Phos-tag gel, I don't see how the ratio can be higher in SAK1-E50K than in WT. SAK1-E50K has a more prominent slower migrating band, but also seems to have a faster migrating band of considerable intensity, whereas I don't see this in the WT lane. I would appreciate it if the authors provided a little more detail on the quantification in the legend.

As indicated in the y-axis legend, we represent the phospho/dephospho ratio. This ratio was calculated by quantifying the intensities of the slower- and faster-migrating bands, as described in the Materials and Methods section. For clarification, we have added the following sentence to the figure legend 5B: "Means and SEMs of the ratio of slower and faster migrating bands from three independent biological experiments are shown".

Regarding the visibility of the faster-migrating band in the WT sample, we acknowledge that it is not readily distinguishable by eye in the representative image. However, it becomes visible in longer-exposure images and has been quantified. We provide below the quantified values used to calculate the ratios in one representative replicate. As can be observed, the faster migrating band was quantified in all the strains.

WT	Up (Esp1-P)	23,762	1.52791887	1
	Down (Esp1)	15,552		
SAK1-E50K	Up (Esp1-P)	22,995	2.31114802	1.512611738
	Down (Esp1)	9,950		
DSAK1	Up (Esp1-P)	19,547	0.645194	0.422269806
	Down (Esp1)	30,296		

Importantly, the quantification reflects the results from three independent biological replicates, ensuring the robustness of the measurement.

During the revision process, we also identified an error in the representation of the p-values in the figure, although they were correctly reported in the main text. In Fig. 5B the differences between WT and *sak1-E50K* are statistically significant.

Following editorial recommendation, we have recalculated the phosphorylation values using the phosphorylated Esp1 signal relative to the total Esp1 signal (phosphorylated + dephosphorylated Esp1). The revised analysis still shows a reduction in Esp1 phosphorylation in *the* ***sak1Δ*** mutant. In contrast, the increase observed in the ***sak1-E50K*** strain did not reach statistical significance.

In addition, we have expanded the Materials and Methods section to provide a detailed description of the densitometric quantification procedure as follows:

"For the phosphorylation analysis of Esp1, lanes were selected using the Gel Analyzer tool in Fiji/ImageJ. Equal-width rectangular selections were drawn around each lane and added sequentially using the Analyze > Gels > Select First Lane *and*Analyze > Gels > Select Next Lane *commands. Densitometric profiles were then generated using* Analyze > Gels > Plot Lanes. *Background correction was performed using the standard ImageJ Gel Analyzer procedure. For each lane profile, the baseline was manually defined by drawing straight lines between the edges of the peaks corresponding to the Esp1 signals, thereby subtracting the local background signal. The area of each peak above the baseline was measured using the Gel Analyzer Wand tool. The proportion of phosphorylated Esp1 was calculated as the area of the phosphorylated isoform signal (low-electrophoretic-mobility band) divided by the sum of the areas of the phosphorylated and dephosphorylated isoform signals (low- and high-mobility bands, respectively)."*

(3) The text editing in line 418 - 423 made this hard to understand. The "However, ..." doesn't seem to fit anymore, and the logic isn't clear. I suggest: "... tightly regulated by securin to ensure accurate chromosome segregation during mitosis. Nuclear localization of separase is greatly reduced, but not abolished, in the absence of securin. This suggests an additional mechanism controlling the nuclear localization of separase, and we hypothesized that Sak1-mediated phosphorylation of separase promotes its nuclear localization."

We thank the reviewer for bringing this issue to our attention. We have incorporated the suggested text change.

(4) Line 451 on securin/separase could additionally cite Yu et al., 2021 - but at the authors' discretion.

We thank the reviewer for pointing this out. We agree that this is an important paper demonstrating that securin acts as a direct inhibitor and pseudosubstrate of separase, and we regret the omission. The reference has now been included.

(5) Line 481 - 483: the phrasing is a little odd, and conservation for some of the residues doesn't seem high.
Maybe color-code residues to make it easier to see conservation and show the larger sequence context in the supplement?

We agree with the reviewer that the level of conservation is not uniformly high. We have incorporated dark blue shading to indicate Identical residues and light blue shading to indicate conserved amino acids, and we have explained the color code in the figure legend. In addition, we have revised the sentence in the Results section to: "Sequence alignment of homologous proteins revealed partial conservation of these residues across evolution, suggesting a potential contribution to the yeast Esp1-Sak1 interaction".

(6) Typos introduced when editing the discussion:
Line 556 should be "mitosis" rather than "mitotic."
Line 568 "nucleolar..." should be "nucleolar release."
Line 576: Even though some text has been removed, it could be useful to still refer to the figure showing the independence from Snf1.

We thank the reviewer for pointing this out. We have corrected the typos and incorporated the reference to the figures related to Snf1.

(7) Line 654: Isn't it a "restrictive" temperature if esp1-2 mutants are unable to grow at this temperature?
It could be called "restrictive," and then adding a sentence: "None of the esp1-2 strains with suppressor mutation obtained at 30 ºC grew at 35 ºC or 37 ºC." I think this would be useful as it provides additional information.

We have incorporated the suggested text change.

(8) Figure 10A: The legend mentions rmsd, but I don't see it in the figure. (Entirely possible that I'm overlooking it.)

The rmsd value was lost upon conversion to PDF. We regret not noticing this earlier, as they were present in the original SVG files. The labels have now been placed on an upper layer, ensuring they are preserved in the PDF version
