# Supplementary material for: The AMPK kinase Sak1 activates separase and promotes its nuclear localization during mitosis
Source: J Cell Biol. 2026 Oct 20;225(11):e202505056. doi: 10.1083/jcb.202505056 (PMC13682446; doi:10.1083/jcb.202505056)

SourceDataF1

1A

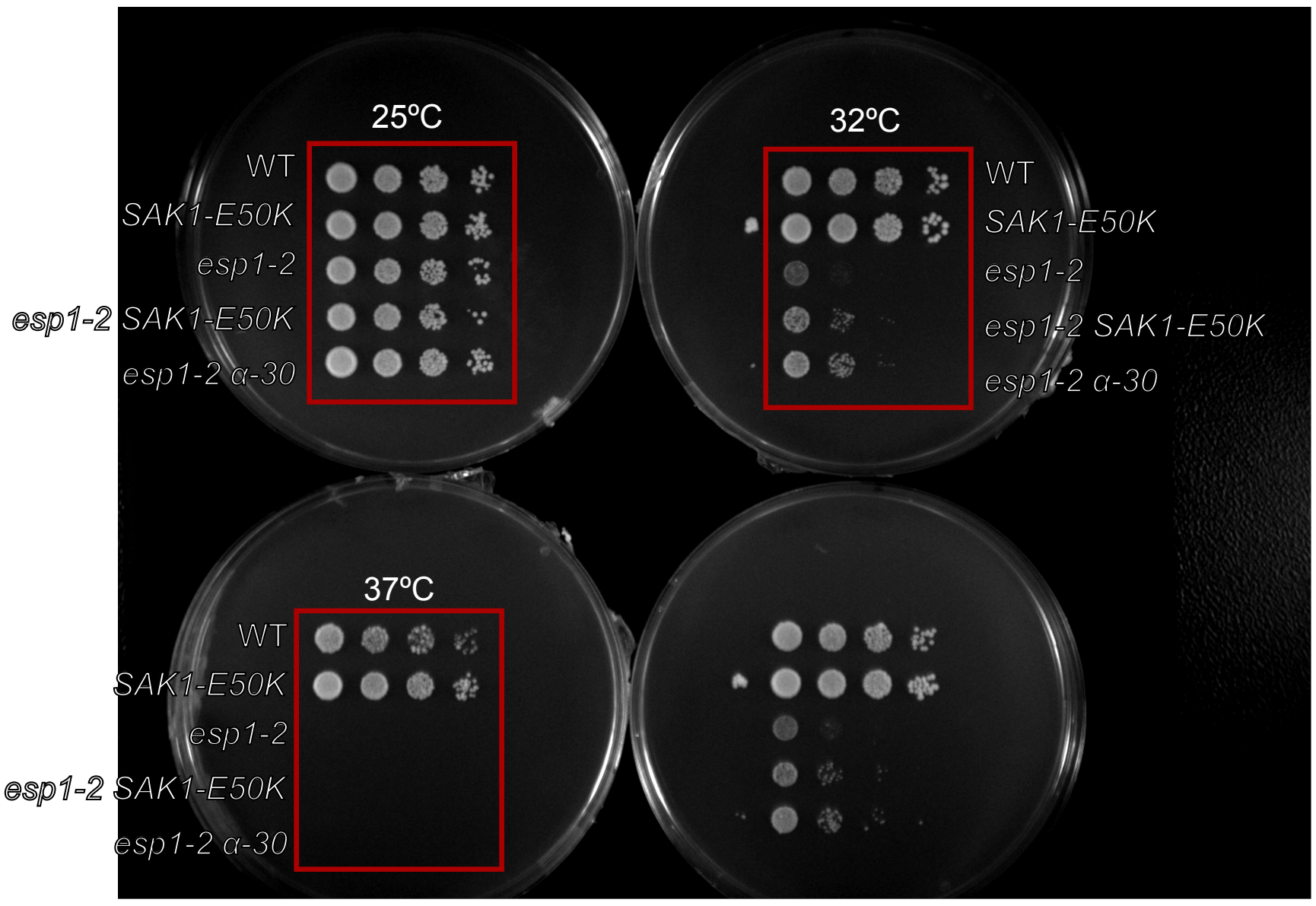

1B

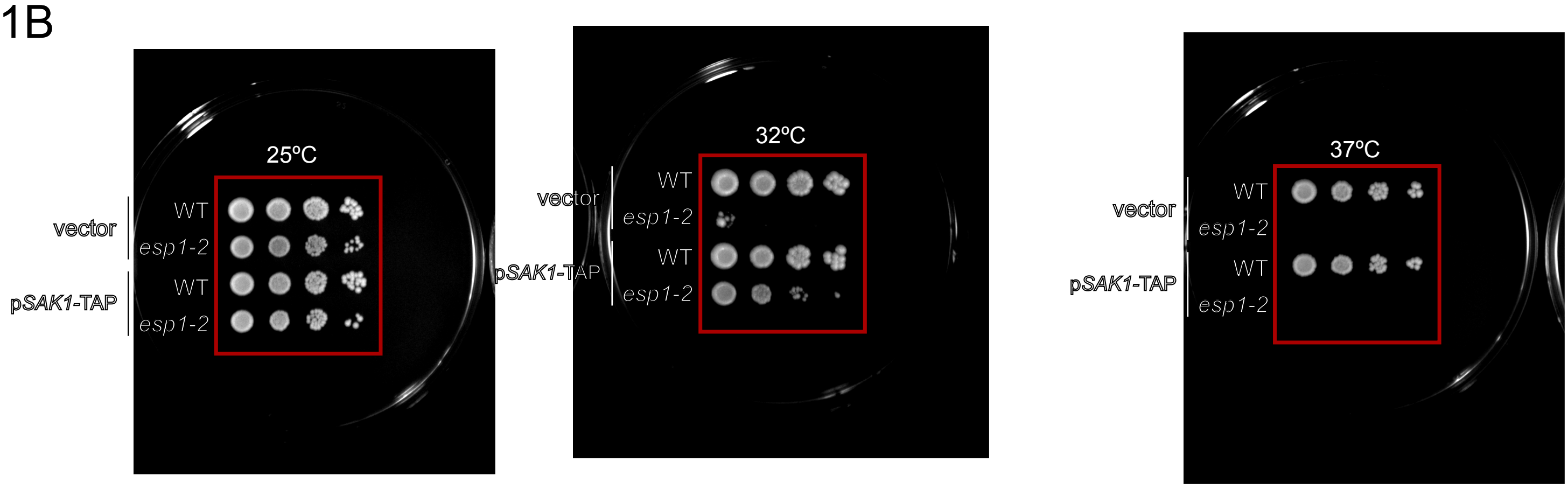

1C

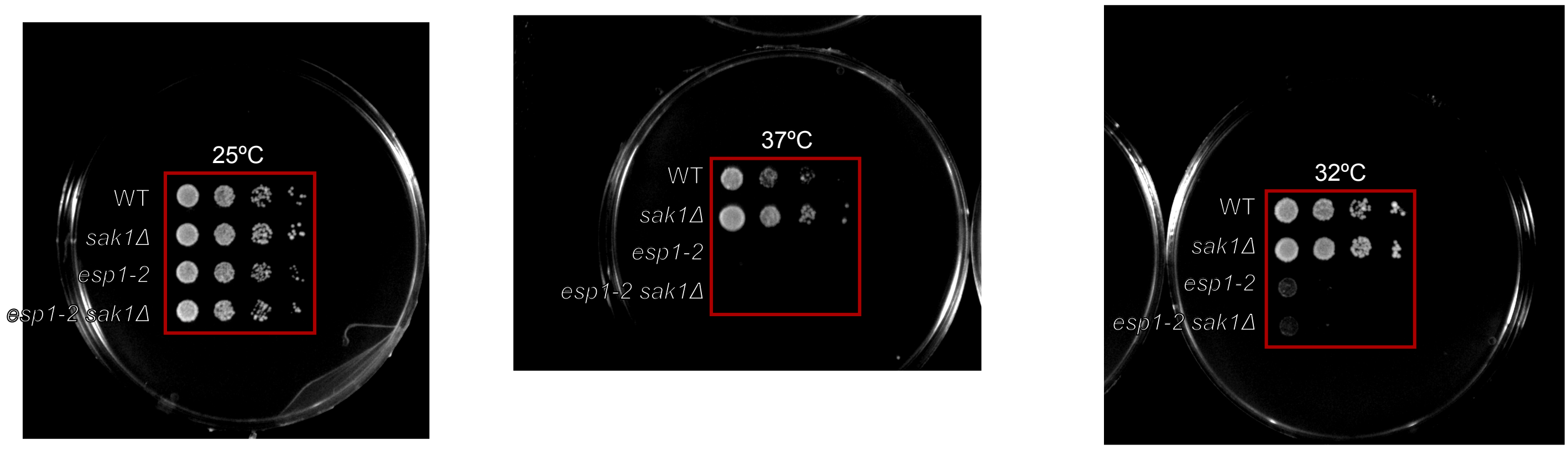

1D

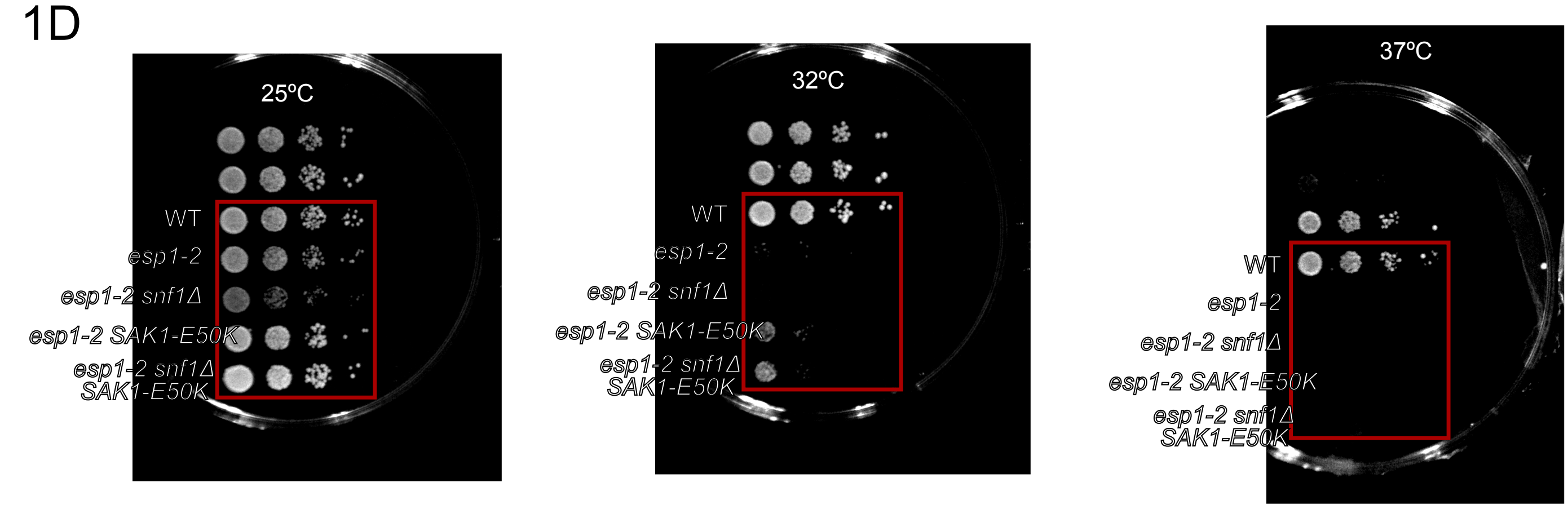

Supplement: Supplementary file 2 [file jcb_202505056_sourcedataf1.pdf]
