# Supplementary material for: The AMPK kinase Sak1 activates separase and promotes its nuclear localization during mitosis
Source: J Cell Biol. 2026 Oct 20;225(11):e202505056. doi: 10.1083/jcb.202505056 (PMC13682446; doi:10.1083/jcb.202505056)

SourceDataF3

3D

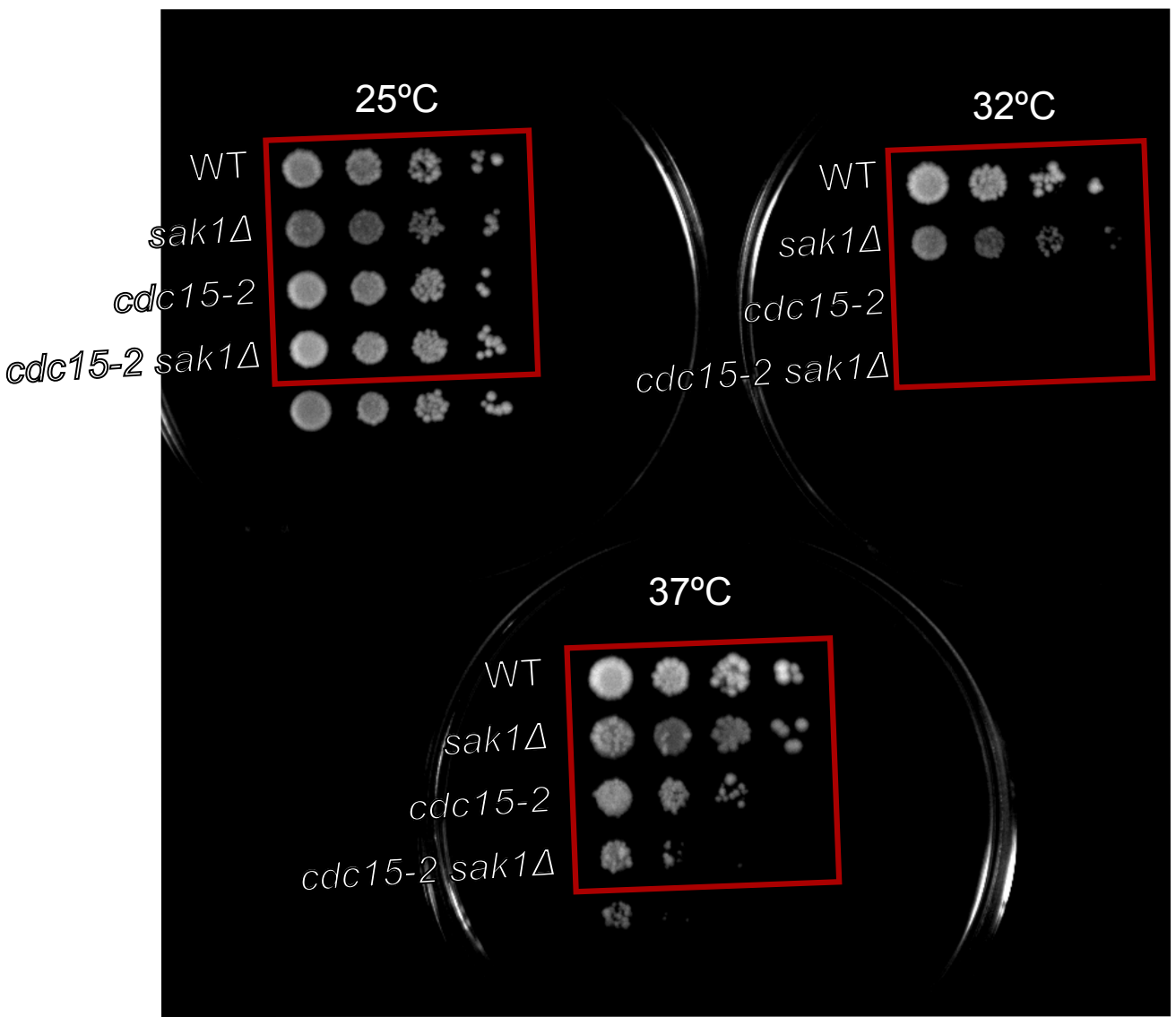

3E

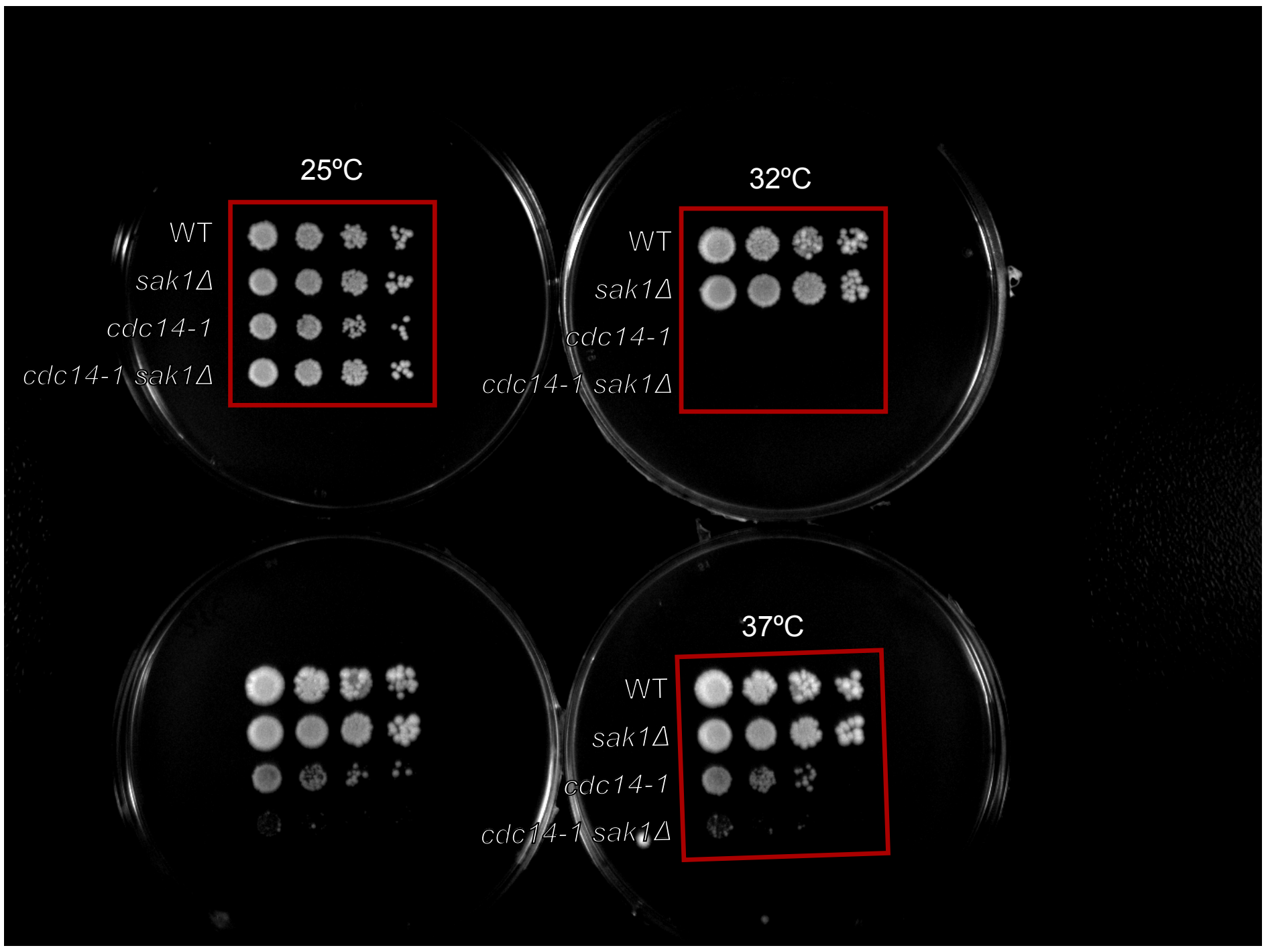

Supplement: Supplementary file 5 [file jcb_202505056_sourcedataf3.pdf]
