# Supplementary material for: The AMPK kinase Sak1 activates separase and promotes its nuclear localization during mitosis
Source: J Cell Biol. 2026 Oct 20;225(11):e202505056. doi: 10.1083/jcb.202505056 (PMC13682446; doi:10.1083/jcb.202505056)
Supplement: Supplementary file 14 [file jcb_202505056_tables3.docx]

| **Strain** | **Genotype** | **Origin** |
| --- | --- | --- |
| W303 | Mat a, *ade2-1*, *trp1-1*, *can1-100*, *leu2-3*,*112*, *his3-11,15*, *ura3-1* | This laboratory |
| Y1698 | Mat alpha, *SAK1-E50K* | This study |
| Y500 | Mat a, *esp1-2* | This laboratory |
| Y1692 | Mat alpha, *esp1-2*, *SAK1-E50K* | This study |
| Y1052 | Mat alpha, *sak1Δ*::*LEU2* | This study |
| Y1393 | Mat a, *esp1-2*, *sak1Δ*::*TRP1* | This study |
| Y2132 | Mat a, *esp1-2*, *snf1Δ*::*URA3* | This study |
| Y2134 | Mat a, *esp1-2*, *SAK1-E50K-6V5::KanMX4, snf1Δ::URA3* | This study |
| Y1656 | Mat a, *GAL1*-*CDC20*::*LEU2* | This laboratory |
| Y1686 | Mat a, *esp1-2*, *GAL1*-*CDC20*::*LEU2* | This laboratory |
| Y1694 | Mat a, *esp1-2*, *SAK1-E50K*, *GAL1*-*CDC20*::*LEU2* | This study |
| Y1955 | Mat a, *ESP1-6HA::HIS3, SAK1-6V5::TRP1, MET3-CDC20::URA3* | This study |
| Y1944 | Mat a, *esp1-2-6HA::HIS3, MET3-CDC20::URA3* | This study |
| Y1947 | Mat a, *esp1-2-6HA::HIS3, SAK1-E50K, MET3-CDC20::URA3* | This study |
| Y2086 | Mat a, *ESP1-6HA::HIS3, MET3-CDC20::URA3* | This study |
| Y1927 | Mat alpha, *esp1-2, SCC1-6HA::HIS3, MET3-CDC20::LEU2* | This study |
| Y1930 | Mat a, *esp1-2, SAK1-E50K, SCC1-6HA::HIS3, MET3-CDC20::URA3* | This study |
| Y1926 | Mat a, *SCC1-6HA::HIS3, MET3-CDC20::URA3* | This study |
| Y1934 | Mat a, *PDS1-6HA::HIS3 MET3-CDC20::LEU2* | This study |
| Y2123 | Mat alpha, *esp1-2, PDS1-6HA::HIS3, MET3-CDC20::LEU2* | This study |
| Y2121 | Mat a, *esp1-2, SAK1-E50K, PDS1-6HA::HIS3, MET3-CDC20::LEU2* | This study |
| Y2049 | Mat a, *SIC1-6HA::HIS3, MET3-CDC20::URA3* | This study |
| Y2113 | Mat alpha, *esp1-2, SIC1-6HA::HIS3, MET3-CDC20::LEU2* | This study |
| Y2116 | Mat alpha, *esp1-2, SAK1-E50K, SIC1-6HA::HIS3, MET3-CDC20::LEU2* | This study |
| Y1916 | Mat a, *esp1-2*, *MET3*-*CDC20*::*LEU2* | This study |
| Y1919 | Mat a, *esp1-2*, *SAK1-E50K*, *MET3*-*CDC20::URA3* | This study |
| Y1056 | Mat alpha, *MET3*-*CDC20*::*LEU2* | This laboratory |
| Y1917 | Mat alpha, *sak1Δ*::*LEU2*, *MET3*-*CDC20::URA3* | This study |
| Y1737 | Mat alpha, *NET1-mCherry::HIS3, CDC14-eGFP::HIS3, SPC42-eYFP::HIS3, GAL1-CDC20::LEU2* | This study |
| Y1712 | Mat a, *NET1-mCherry::HIS3, CDC14-eGFP::HIS3, SPC42-eYFP::HIS3, sak1Δ::TRP1, GAL1-CDC20::LEU2* | This study |
| Y509 | Mat a, *cdc15-2* | This laboratory |
| Y1778 | Mat alpha, *sak1Δ*::*LEU2*, *cdc15-2* | This study |
| Y1262 | Mat a, *cdc14-1* | A. Toh-e lab |
| Y1836 | Mat alpha, *cdc14-1*, *sak1Δ*::*TRP1* | This study |
| Y1926 | Mat a, *SCC1-6HA::HIS3, MET3-CDC20::LEU2* | This study |
| Y1929 | Mat alpha, *SAK1-E50K, SCC1-6HA::HIS3, MET3-CDC20::URA3* | This study |
| Y1937 | MAT alpha, *SAK1-E50K, PDS1-6HA::HIS3, MET3-CDC20::URA3* | This study |
| Y2051 | Mat alpha, *SAK1-E50K, SIC1-6HA::HIS3, MET3-CDC20::URA3* | This study |
| Y2052 | Mat a, *GAL1-FLAG-ESP1-CBD::TRP1, SCC1-6HA::HIS3 MET3- CDC20::URA3* | This study |
| Y2058 | Mat alpha, *GAL1-FLAG-ESP1-CBD::TRP1, sak1Δ::LEU2, SCC1-6HA::HIS3, MET3-CDC20::URA3* | This study |
| Y2059 | Mat alpha, *GAL1-FLAG-ESP1-CBD::TRP1, SAK1-E50K-6V5::TRP1, SCC1-6HA::HIS3, MET3-CDC20::URA3* | This study |
| Y2037 | Mat alpha, *GAL1-FLAG-ESP1-CBD::TRP1, PDS1-6HA::HIS3, MET3-CDC20::URA3* | This study |
| Y2033 | Mat alpha, *GAL1-FLAG-ESP1-CBD::TRP1, sak1Δ::LEU2, PDS1-6HA::HIS3, MET3-CDC20::URA3* | This study |
| Y2032 | Mat alpha, *GAL1-FLAG-ESP1-CBD::TRP1, SAK1-E50K-6V5::TRP1, PDS1-6HA::HIS3, MET3-CDC20::URA3* | This study |
| Y2036 | Mat alpha, *GAL1-FLAG-ESP1-CBD::TRP1, SIC1-6HA::HIS3, MET3-CDC20::URA3* | This study |
| Y2034 | Mat alpha, *GAL1-FLAG-ESP1-CBD::TRP1, sak1Δ::LEU2, SIC1-6HA::HIS3, MET3-CDC20::URA3* | This study |
| Y2027 | Mat alpha, *GAL1-FLAG-ESP1-CBD::TRP1, SAK1-E50K-6V5::TRP1, SIC1-6HA::HIS3, MET3-CDC20::URA3* | This study |
| Y1964 | Mat alpha, *ESP1-6HA::HIS3, SAK1-E50K-6V5::TRP1, MET3-CDC20::URA3* | This study |
| Y2552 | Mat alpha, *sak1Δ*::*LEU2, pYIP-SAK1-6V5::HIS3* | This study |
| Y2553 | Mat alpha, *sak1Δ*::*LEU2, pYIP-SAK1-D277A-6V5::HIS3* | This study |
| Y1922 | Mat a, *ESP1-6HA::HIS3, SAK1-6V5::TRP1* | This study |
| Y1942 | Mat alpha, *ESP1-6HA::HIS3, SAK1-E50K* | This study |
| Y1941 | Mat alpha*, sak1Δ::LEU2, ESP1-6HA::HIS3* | This study |
| Y2095 | Mat alpha, *HTB2-mCherry::URA3, ESP1-eGFP::HIS3, MET3-CDC20::LEU2* | This study |
| Y2105 | Mat alpha, *SAK1-E50K, ESP1-eGFP::HIS3, MET3-CDC20::URA3* | This study |
| Y2117 | Mat alpha, *sak1Δ::LEU2, ESP1-eGFP::HIS3, MET3-CDC20::URA3* | This study |
| Y2162 | Mat alpha, *pds1Δ::TRP1, HTB2-mCherry::URA3, ESP1-eGFP::HIS3, MET3-CDC20::LEU2* | This study |
| Y2188 | Mat a, *HTB2-mCherry::URA3, ESP1-eGFP::HIS3* | This study |
| Y2161 | Mat a, *SAK1-E150K, ESP1-eGFP::HIS3* | This study |
| Y2362 | Mat a, *pds1Δ::TRP1, HTB2-mCherry::URA3, ESP1-eGFP::HIS3* | This study |
| Y2363 | Mat a, *pds1Δ::TRP1, SAK1-E50K, ESP1-eGFP::HIS3* | This study |
| Y2295 | Mat a, *ESP1-6HA::HIS3, SAK1-6V5::TRP1, PDS1-9myc::URA3* | This study |
| Y2127 | Mat a, *ESP1-6HA::HIS3, PDS1-9myc::URA3* | This study |
| Y2126 | Mat alpha, *SAK1-E50K, ESP1-6HA::HIS3, PDS1-9myc::URA3* | This study |
| Y2408 | Mat a, *ESP1-6HA::HIS3, PDS1-9myc::URA3, sak1Δ::LEU2* | This study |
| Y2294 | Mat a, *PDS1-9myc::URA3* | This study |
| Y2290 | Mat a, *esp1-2-6HA::HIS3, SAK1-E50K, PDS1-9myc::URA3* | This study |
| Y2564 | Mat alpha, *pYIP-ESP1-eGFP::URA3, pYIP-SAK1-6V5::HIS3, MET3-CDC20::LEU2* | This study |
| Y2565 | Mat alpha, *pYIP-esp1-D1206A-D1350A-eGFP::URA3, pYIP-sak1-R151A-K156A-6V5::HIS3, MET3-CDC20::LEU2* | This study |
| Y2559 | Mat alpha, *pYIP-ESP1-eGFP::URA3, MET3-CDC20::LEU2* | This study |
| Y1971 | Mat a, *GAL1-FLAG-ESP1-CBD::TRP1 sak1Δ::LEU2, SIC1-6V5::HIS3, MET3-CDC20::TRP1* | This study |
| Y1655 | Mat alpha, *sak1Δ*::*LEU2*, *GAL1*-*CDC20::URA3* | This study |
| Y1686 | Mat a, *esp1-2*, *GAL1*-*CDC20*::*LEU2* | This study |
| Y1779 | Mat a, *GAL1-SAK1-TAP::TRP1* | This study |
| Y1790 | Mat a, *esp1-2, GAL1-SAK1-TAP::TRP1* | This study |
| Y1793 | Mat a, *esp1-2, GAL1-SAK1-TAP::TRP1, MET3-CDC20::LEU2* | This study |
| Y2038 | Mat a, *GAL1-SAK1-TAP::TRP1, ESP1-6HA::HIS MET3-CDC20::LEU2* | This study |
| Y2231 | Mat a, *sak1Δ::LEU2, HTB2-mCherry::URA3, ESP1-eGFP::HIS3* | This study |
| Y1785 | Mat alpha, *GAL1-SAK1-TAP::TRP1, MET3*-*CDC20*::*LEU2* | This study |
| Y1792 | Mat alpha, *GAL1-SAK1-TAP::TRP1* | This study |
| Y1809 | Mat a, *cdc15-2, GAL1-SAK1-TAP::TRP1* | This study |
| Y2085 | Mat alpha, *HTB2-mCherry::URA3, MET3-CDC20::LEU2* | This study |

**Table S3. List of strains used in this study.**
